# Dynamic Modelling of DNA Repair Pathway at the Molecular Level: A New Perspective

**DOI:** 10.3389/fmolb.2022.878148

**Published:** 2022-09-13

**Authors:** Paola Lecca, Adaoha E. C. Ihekwaba-Ndibe

**Affiliations:** ^1^ Faculty of Computer Science, Free University of Bozen-Bolzano, Bolzano, Italy; ^2^ Faculty of Health and Life Sciences, Coventry University, Coventry, United Kingdom

**Keywords:** DNA damage, DNA repair genes, dynamical networks, ODE models, parametric sensitivity analysis, centrality measure analysis

## Abstract

DNA is the genetic repository for all living organisms, and it is subject to constant changes caused by chemical and physical factors. Any change, if not repaired, erodes the genetic information and causes mutations and diseases. To ensure overall survival, robust DNA repair mechanisms and damage-bypass mechanisms have evolved to ensure that the DNA is constantly protected against potentially deleterious damage while maintaining its integrity. Not surprisingly, defects in DNA repair genes affect metabolic processes, and this can be seen in some types of cancer, where DNA repair pathways are disrupted and deregulated, resulting in genome instability. Mathematically modelling the complex network of genes and processes that make up the DNA repair network will not only provide insight into how cells recognise and react to mutations, but it may also reveal whether or not genes involved in the repair process can be controlled. Due to the complexity of this network and the need for a mathematical model and software platform to simulate different investigation scenarios, there must be an automatic way to convert this network into a mathematical model. In this paper, we present a topological analysis of one of the networks in DNA repair, specifically homologous recombination repair (HR). We propose a method for the automatic construction of a system of rate equations to describe network dynamics and present results of a numerical simulation of the model and model sensitivity analysis to the parameters. In the past, dynamic modelling and sensitivity analysis have been used to study the evolution of tumours in response to drugs in cancer medicine. However, automatic generation of a mathematical model and the study of its sensitivity to parameter have not been applied to research on the DNA repair network so far. Therefore, we present this application as an approach for medical research against cancer, since it could give insight into a possible approach with which central nodes of the networks and repair genes could be identified and controlled with the ultimate goal of aiding cancer therapy to fight the onset of cancer and its progression.

## 1 Introduction

DNA molecules packaged in our chromosomes carry our genetic blueprint, and their preservation is essential for the coordination of cellular function and organization of life (Branzei and Foiani, 2008). As DNA is the repository of our genetic information, we would expect its structure to be highly stable. This is not the case for the DNA (Reed and Waters, 2005). Damage to DNA is a constant threat (Lindahl, 1993; Alberts, 2015). The DNA molecule is intrinsically reactive, as it is very susceptible to chemical and physical factors, which can lead to DNA lesions, such as base loss, base modification, and double-strand DNA breaks (Hoeijmakers, 2009; Çağlayan and Wilson, 2015; Ross and Truant, 2016; Yadav et al., 2020). Physiological conditions such as oxygen-rich, aqueous, or pH 7.4 (Lindahl, 1993) as well as chemical events such as hydrolysis and exposure to reactive oxygen species (ROS) or other reactive metabolites can damage DNA. Exogenous chemicals or endogenous metabolic processes trigger chemical reactions. Although exogenous stressors can be extremely powerful, endogenous threats are constant and unabating. It is estimated that a single cell experiences up to 10^5^ spontaneous or induced DNA lesions per day (Lindahl, 1993; Bont, 2004; Kovalchuk, 2016; Chatterjee and Walker, 2017).

DNA damage has far-reaching consequences, such as preventing RNA polymerase from transcribing the correct messenger RNA sequence to produce the correct protein. In the longer term, cellular malfunctions such as cancer initiation, inborn defects, and ageing that result after damaged DNA replicates are examples of unpredictable long-term consequences of DNA damage; as base misincorporation causes mutations which alter the genetic code (Reed and Waters, 2005). Therefore, a coordinated response to DNA damage is necessary in order to ensure cellular viability and prevent diseases. Cells, fortunately, possess a robust system of mechanisms that function together to reduce the adverse consequences of DNA damage and ensure that their genetic information is faithfully replicated, thus maintaining the integrity of their genome (Ganai and Johansson, 2016). This coordinated effort, known as DNA damage response (DDR) operates by sensing and signalling the genotoxic events, and the damage is then resolved either by DNA repair machineries, or cell death if DNA cannot be repaired. DNA repair functions as part of the DNA damage response (DDR) (Liu, 2001; Chatterjee and Walker, 2017; Reed and Waters, 2005; Hoeijmakers, 2001, 2009).

DNA repair has so far been shown to exist in both prokaryotic and eukaryotic organisms, with over 150 proteins directly involved in safeguarding the genome (Sancar et al., 2004; Friedberg et al., 2005; Wood et al., 2005; Yousefzadeh et al., 2021). DNA repair processes restore DNA back to its normal sequence and structure after damage (Friedberg et al., 2006), and are characterised traditionally by the type of damage they repair. There are five major DNA repair pathways available to cells to deal with DNA damage burdens. Each of these processes recognises a particular type of DNA lesions, and together work in preventing mutagenesis. They include 1) direct reversal repairs that repairs lesion induced mainly by alkylating agents, 2) Base excision repair (BER), for small base modifications like single-strand breaks (SSBs) and non-bulky damaged DNA bases, 3) Nucleotide excision repair (NER), that corrects bulky, helix-distorting DNA lesions, 4) mismatch repair (MMR), that repairs base-base mismatch and insertion or deletion loops (IDLs), 5) Recombinational repair, which is divided into non-homologous end joining (NHEJ) and homologous recombination repair (HR), both of which repairs DNA double-strand breaks (DSBs). Other types of DSB repairs include alternative non-homologous end-joining (alt-NHEJ, MMEJ) and translesion synthesis (TLS), which operates as a tolerance mechanism for DNA damage (Jackson and Bartek, 2009; Hosoya and Miyagawa, 2014; Li et al., 2021).

For frequently occurring DNA damage, direct reversal of DNA damage by specialised proteins is the most efficient and most straightforward method of DNA repair. However, this approach is only used by a small proportion of DNA repair types. Most damage to DNA is repaired by the removal of damaged bases and is followed by resynthesis of the removed/excised region (replacement) (Cooper, 2000). The pathways involved in the removal of base damage are base excision repair (BER), nucleotide excision repair (NER) and mismatch repair (MMR) (Cooper, 2000). The rest of the pathways repair damage to DNA structure/backbone. DNA damage can cause breaks in the DNA backbone, single-strand breaks in one strand, or double-strand breaks on both strands. Single-strand breaks are repaired by mechanisms sharing common steps in the BER pathway; however, DSBs are especially harmful as, by definition, no unbroken complementary strand exit which can serve as a template for repair when both strands break (Bennett et al., 1993; Friedberg et al., 2006). For cells with DNA already replicated prior to cell division, the duplicate copy can easily supply the missing information. So in these cells, DSBs can be repaired by HR, involving the exchange of DNA strands.

Even so, very efficient repair mechanisms can sometimes fail to provide a clean template for DNA synthesis. Replication errors can make it past these mechanisms, as DNA repair can also undergo mutations and become dysregulated. DNA repair gene mutations have been known to cause a variety of rare inherited human syndromes. Some of which include premature ageing phenotypes, increased sensitivity to ionising radiation exposure, and increased cancer risk (Friedberg et al., 2006; Lok and Powell, 2012; Carusillo and Mussolino, 2020). It has also been found that inherited defects in each of the DNA repair pathways are associated with distinct genome instability syndromes (Yousefzadeh et al., 2021), syndromes characterised by developmental defects (Bouwman and Jonkers, 2012; Ghosal and Chen, 2013; Wolters and Schumacher, 2013; Wood, 2018).

The dysregulation of DNA repair gene networks underlies many human genetic diseases that affect a wide range of body systems but all share a common trait, predisposition to cancer (Chatterjee and Walker, 2017). Almost all human cancers are spontaneous, not inherited, and are caused by environmental or genetic factors. It is of great public health interest to determine which genetic variations increase cancer risk in normal populations, and DNA repair genes are likely contenders. Therefore, elucidating the molecular mechanism behind DNA repair defects may provide a framework for understanding the complex pattern of genetic variations that contribute to spontaneous human cancers.

In this study, we demonstrate how to translate a network (mathematically definable as a hyper-graph) into a set of first-order differential equations of the mass action law type. Once the model has been established, we present its numerical solution and carry out a sensitivity analysis of its kinetic rates, whose numerical values are mostly unknown. The results of the analysis of network dynamics complement those produced by the calculation of centrality measures, and together they produce a set of genes of similar biological interest, and in perspective also of medical interest, due to their characteristics of topological centrality and vulnerability to stimuli. The paper is organized as follows: in Section 2 we describe the mechanisms of double-strand break repair pathway homologous recombination repair necessary to understand and interpret the results of the computational analysis, in Section 3 we describe the rules on which the automatic translation of the network into a rate equation system is based and the methods of sensitivity analysis of the model. In Section 4 we present the results of the analysis, and, finally, in Section 5, we draw some conclusions.

## 2 Double-Strand Break Repair Pathway Homologous Recombination Repair

DSBs are the most serious DNA damage, as both DNA strands are impaired simultaneously. Therefore, due to the magnitude of differing factors leading to DSBs, the effectiveness of their repair is crucial for cell survival and the functioning and prevention of DNA fragmentation, chromosomal translocation and deletion. DSBs can be repaired in mammalian cells by NHEJ, HR, and single-strand annealing (SSA). Unrepaired SSBs result in much more cytotoxic DSBs formation during the S-phase progression of the cell cycle (Kennedy and D’Andrea, 2006). Homologous recombination is a process by which DSBs are repaired through the alignment of homologous sequences of DNA (Dietlein and Reinhardt, 2014) and occurs primarily during the late S to G2 phase of the cell cycle (Cerbinskaite et al., 2012; Chatterjee and Walker, 2017).

Homologous recombination is the second major DSB repair pathway and requires a second, homologous DNA sequence to function as donor template. There are two phases to this process, the first phase triggered by sensor proteins that belong to the MRN complex, and the second phase by the stimulation of resection steps, initiated in the first phase and subsequently extended. HR generally involves the following stages:

1. DSBs are recognised and sensed by the MRN complex (Kim et al., 1994), which activates ATM kinase, initiating the DSB end resection steps, where CtBP-interacting protein (CtIP) and the MRN complex work together to generate single-strand DNA (ssDNA) at the DSB ends ((Zhao et al., 2020).
2. The exposed ssDNA is recognised by and coated with DNA replication protein A (RPA) complex, which recruits the major homologous recombination regulator RAD52 to the site to facilitate HR repair (Maréchal and Zou, 2014; Rossi et al., 2021).
3. The nucleoprotein filament RAD51, is then assembled, mediated by BReast CAncer type 2 susceptibility protein (BRCA2), to replace RPA on ssDNA to perform homology sequence searching and strand invasion (Kowalczykowski, 2015).
4. DSBs are then restored by branch migration, DNA synthesis, ligation, and resolution of Holliday junctions (Zhao et al., 2020).

Following the recognition and sensing of DSBs, a process known as DNA end resection is activated, a critical function in HR (Liu and Huang, 2016; Zhao et al., 2020). DNA end resection catalyses the nucleolytic degradation of the broken ends of DSBs (by the CtIPMRN complex) in the 5′ to 3′ direction generating 3′ single-stranded DNA (ssDNA). The 3′ ssDNA then provides a platform for the recruitment of HR repair-related proteins (Huertas, 2010; Liu and Huang, 2016; Zhao et al., 2020). Following the generation of ssDNA, downstream nucleases and helicases, such as exonuclease 1 (EXO1) or DNA replication ATP-dependent helicase/nuclease DNA replication helicasenuclease 2 (DNA2) and Bloom syndrome protein (BLM), are conscripted to extend the 3’ ssDNA for HR repair (Huertas and Jackson, 2009; Yun and Hiom, 2009; Zhao et al., 2020). The identities of these DNA helicases and nucleases are yet to be clearly defined in humans (as in yeast), partly because there are many candidate proteins. Although five RecQ helicase homologs have so far been identified in yeast (Bloom helicase [BLM], Werner helicase/nuclease [WRN], RECQ1, RECQ4, and RECQ5) (Chu and Hickson, 2009; Lu and Davis, 2021), convincing evidence point up BLM in resection (Gravel et al., 2008; Nimonkar et al., 2008). Following resection, the exposed single-strand DNA (ssDNA) is recognised and bound by RPA complex for protection.

RPA plays a significant role in coordinating DNA resection processes and simultaneously preserving the integrity of the resultant ssDNA (Sun et al., 2019). RPA is a heterotrimeric ssDNA binding protein essential to nearly all DNA processing events and associates with ssDNA with very high affinity (Kd sim 109–1010 M) (Maréchal and Zou, 2014). It is comprised of three protein subunits, RPA70, RPA32 and RPA14 and contains multiple oligonucleotideoligosaccharide (OB)-folds that interact with both ssDNA and proteins (Kim et al., 1994; Fanning, 2006; Feldkamp et al., 2014; Maréchal and Zou, 2014). RPA is flexible (Brosey et al., 2013). Its versatile nature allows it to coordinate the recruitment, activation and exchange of many proteins whose combined activities allow for the protection and propagation of eukaryotic genomes (Maréchal and Zou, 2014). How multiple RPAs associate on ssDNA and coordinate its vast array of processes remains to be determined (Sun et al., 2019). However, a critical feature of RPA is that, though it can bind nucleic acids with very high affinity, it can easily be displaced by other enzymes for further downstream processing (Sun et al., 2019).

**FIGURE 1:**
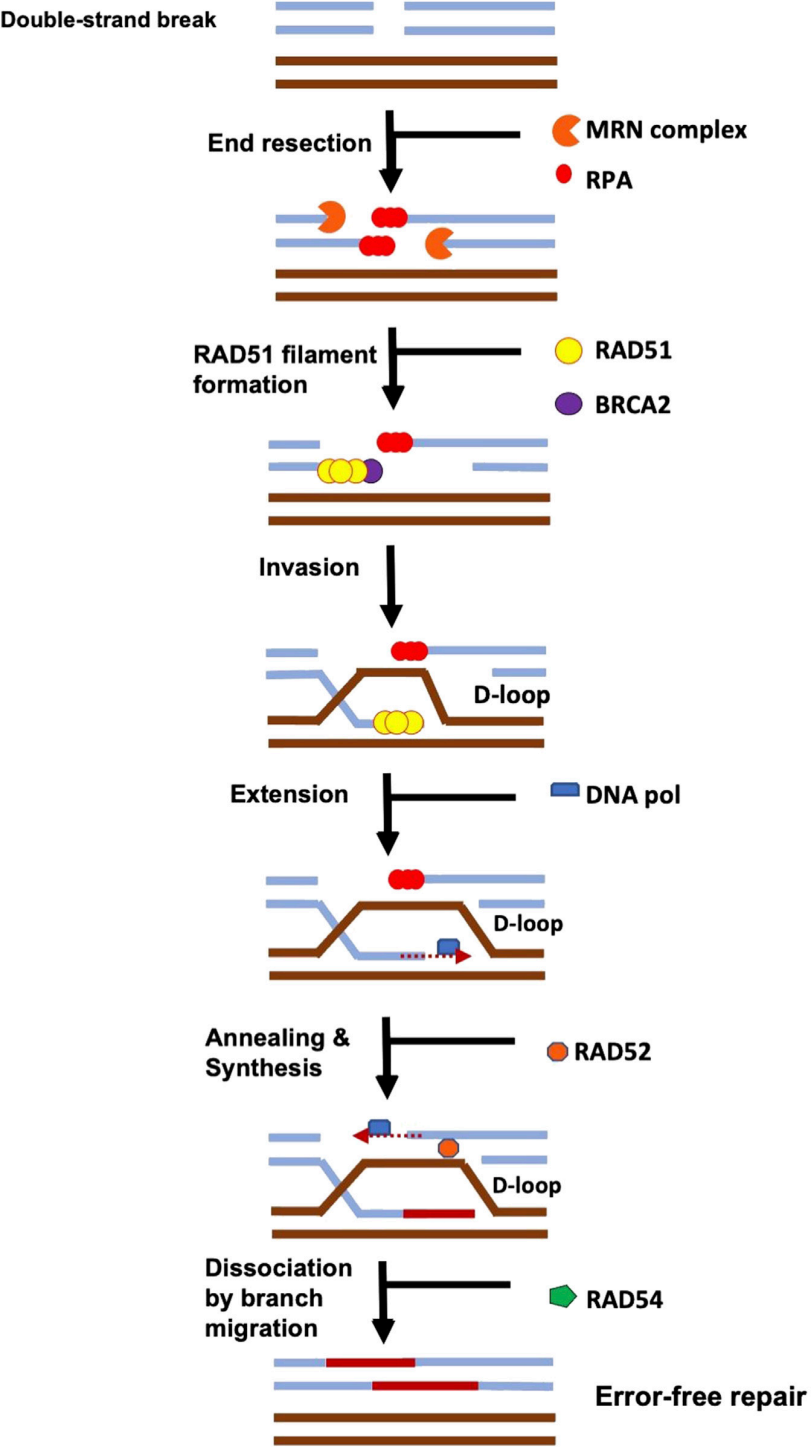
The signalling of a DSB is initiated via the binding of the MRN complex which initiates resection. During HR, the ends of the double-strand break (DSB) are resected by nucleases, exposing single-strand DNA (ssDNA) that becomes bound by RPA. The mediator protein, BRCA2 initiates the loading of RAD51 onto ssDNA, helping to displace RPA. RAD51 oligomerizes, forms a nucleoprotein filament, and then searches for the homologous DNA sequence on the intact chromosome. RAD51 filament invades the intact dsDNA and forms a D-loop structure. It is further processed by DNA polymerases, chromatin remodelers (RAD54), nucleases, and ligases to restore it back to its original sequences. (Adapted from (Rossi et al., 2021).

When ssDNA length is sufficient for HR repair, the end resection process is terminated (Zhao et al., 2015). Although the regulation of DNA end resection termination are not yet clearly understood, several studies suggest that under physiological conditions, end resection is terminated by RAD51-RPA switching (Zhao et al., 2015). This switching is regulated by BRCA2-DSS1. DSS1 - SEM1 in yeast - is a small, highly acidic protein that competes with ssDNA, by mimicking ssDNA in order to remove RPA from the genuine ssDNA (Zhao et al., 2015; Stefanovie et al., 2019; Le et al., 2020; Rossi et al., 2021). The DSS1 then binds to BRCA2 in order to facilitate RAD51 filament formation (Liu et al., 2010; Stefanovie et al., 2019; Rossi et al., 2021). DSS1 does not seem to bind DNA on its own but appears to enhance ssDNA binding activities of BRCA2 and RAD52 to promote DSB repair (Zhao et al., 2015). BRCA2 then recruits RAD51 to complete the switch (Zhao et al., 2015; Rossi et al., 2021). For cells with DNA already replicated prior to cell division, RAD51 will oligomerise and form a nucleoprotein filament on the resected, single-stranded DNA (ssDNA) end of the DSB, and search for the homologous DNA sequence on the undamaged sister chromatid, performs strand exchange (invasion), and produce a joint molecule called a D-loop (Rossi et al., 2021). DNA polymerase will then use the homologous DNA strand as a template from the D-loop, and the 3′-end of the broken DNA strand as a primer to commence DNA repair synthesis (Rossi et al., 2021). The other end of the double-strand break is then apprehended by RAD52, joining it to the D-loop, through the annealing process, causing the displaced strand to act as a template for the second strand synthesis (Rossi et al., 2021). When DNA synthesis is complete, the D-loops are then dissociated by RAD54, a protein that interacts with RAD51 to promote branch migration, or interacts with helicases like BLM (van Brabant et al., 2000; Bugreev et al., 2006; Kawale and Sung, 2020; Rossi et al., 2021). DNA is further extended by DNA polymerase, annealed to the ssDNA part of the second broken DNA, gap filled and finally restored (Rossi et al., 2021). In Figure 1 we summarise what is described in this section about the DSB signalling mechanisms.

Homologous recombination is able to repair DSBs error-free using the undamaged sister chromatid (Dietlein and Reinhardt, 2014). As the accuracy of homologous recombination repair is important for DSBs (Sugiyama and Kantake, 2009), if it is impaired, chemotherapeutic opportunities may arise (Huang and Zhou, 2021).

## 3 Graph Representation and Mathematical Model

We considered HDR through Homologous Recombination (HRR) network as available in Pathways Commons (Cerami et al., 2010) in the SIF (Simple Interaction Format) format at the link in the reference (Orlic-Milacic, 2015). See these data also reported in Supplementary Tables S1–S3.

We implemented an R script, that takes as input the HR network and is able to.

• analyse the topology of the network through the calculation of standard and new centrality measures. The standard node centrality measures considered in this study are the degree (in-, out- and total), the betweenness, the clustering coefficient, the eingenvector centrality, the vibrational centrality, the subgraph centrality, and the information centrality (see (Marsden, 2005; Koschützki and Schreiber, 2008; Ghasemi et al., 2014; Wang et al., 2014; Fornito et al., 2016; Jalili et al., 2016; Ashtiani et al., 2018) for a concise but comprehensive report on the meaning and the use of these measure in molecular biology). We considered also a new centrality measures, such as vibrational centrality, introduced by Estrada in (Estrada and Hatano, 2010) that we will discuss in more detail in the next section (we also refer the reader to (Lecca and Re, 2019) for a review on vibrational centrality); for the reader’s convenience, we list the definition of these centrality indices in Supplementary Table S4, that are also extensively covered in many textbooks on graph theory, and in various articles in the applied sciences. We refer the reader to Estrada’s numerous works, a comprehensive compendium of which can be found in the book (Estrada and Hatano, 2010; Estrada, 2011);
• automatically generate a system of rate equations, specifically first order mass action differential equations, describing the dynamics of the network.

and a R script implementing parametric sensitivity analysis of the dynamics model.

In the dynamics model, by default the kinetic rate constants *k* a well as the initial values of the proteins and molecules concentrations are set equal to random values in fixed ranges. Nevertheless these ranges can be modified by the user as shown by the interactive console output reported in Table 1. However, we note that the interval of definition of the uniform distribution cannot exceed the maximal range of parameter variability within which the system of rate equations has a solution. We refer the reader to a previous work of us (Lecca et al., 2016) for more details on this.

**TABLE 1:** Interactive graphical user interface of NADS software showing the options and the task concerning the generation of ODE equations and their solution.

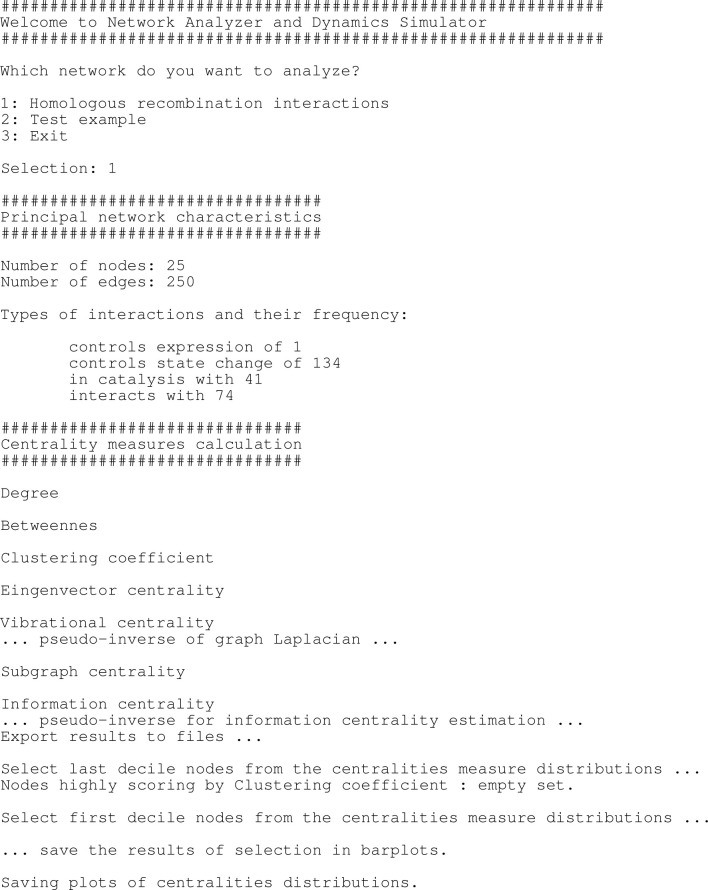

In a first experiment, the initial values of the proteins concentration (not experimentally known) has been drawn randomly in a range [1, 100] a. u., and the simulation time interval was [0, 10] a. u. In a second experiment, the numerical simulation of the model was performed by assigning an initial quantity between 18 and 20 (expressed in arbitrary units a. u.) to each node and for *t* ∈ [0, 1400] (in arbitrary units). The solution of the system of 25 differential equations converges for values of rate constants in the range $\left[\right]0,0.01\left.\right]$ a. u. in the first experiment, and in the range $\left[\right]10^{-6},10^{-5}\left.\right]$ a.u. in the second experiment. To the best of our knowledge, the *in vivo* concentrations of the proteins that are part of the HR network are not known. Combined with the lack of knowledge of the values of rate constants, this means that we cannot assign units to these values that reflect *in vivo* kinetics. However, the initial values of the protein concentrations have been chosen from the order of magnitude at which various *in vitro* experiments operate and express these concentrations in nM and time in seconds. The literature we have referred to includes the works of (Yang et al., 2010; Nguyen et al., 2013; Foertsch et al., 2019).

We changed the parameters one at a time while keeping the values of the others fixed. Since for each parameter *p*_*h*_ (*h* ranges from 1 to the number of parameters in the equations), we sampled *N*_*P*_ values, and consequently we performed *N*_*P*_ model simulations. Let us denote with *x*_*s*_(*t*), (*s* = 1, 2, *…*, *d*) the time series expressing the numerical solutions of the rate equations, where *d* is the number of the proteins in the network. The index of sensitivity of *x*_*s*_(*t*) with respect to the change of *h*-th parameter from the value *p*_*h*_ to the value $p_{h}^{\prime }$ is calculated as in (Lecca et al., 2016) by

$$SI_{ish}=\frac{1}{N}\overset{N}{\underset{k=1}{\sum }}{\left(\frac{1}{N_{P}-1}\overset{N_{P}}{\underset{r=1}{\sum }}{\left(x_{s}^{\left(r\right)}\left(t_{k}|p_{h}\leftarrow p_{h}^{\prime }\right)-{\bar{x_{s}}}^{\left(r\right)}\left(p_{h}\leftarrow p_{h}^{\prime }\right)\right)}^{2}\right)}^{\frac{1}{2}}$$ (1)

where *N* is the length of the time series $x_{s}\left.\right)\mathrm{(t)}\left.\right)$ , and

$$\bar{x_{s}}\left(p_{h}\leftarrow p_{h}^{\prime }\right)=\frac{1}{N_{P}}\overset{N_{P}}{\underset{k=1}{\sum }}x_{s}\left(t_{k}|p_{h}\leftarrow p_{h}^{\prime }\right).$$ (2)

where “ $p_{h}\leftarrow p_{h}^{\prime }$ ” means “*p*_*h*_ replaced by $p_{h}^{\prime }$ ”. The Eq. 1 defines the mean of the standard deviations of the distributions of the simulated values of a protein/gene abundance at time points *t*_*k*_.

In case the user knows the values of the rate constants, he/she can add them as an extra column to the SIF format of the input files. At the moment of writing, for most interactions the values of the kinetic constants are not known and there are no time-resolved data from which it is possible to infer them. It is precisely this context that justifies our choice to study the dynamics of the system in a range of values of the model parameters and more generally to provide a software that can be used as a platform for in silico experiments.

We implemented Network Analyser and Dynamics Simulator (NADS) consists of three modules written in R language:

• network_analysis_functions.R: this module implements the functions that processes the SIF data-frames to make them suitable to their conversion into a graph. This module also implement the functions to rank the nodes according to their centrality measures;
• graph_parser.R: this module converts the SIF format network into an R script that solves the corresponding ordinary differential equations;
• network_analysis.R: this module first calls network_analysis_functions.R and performs the networks analysis, and secondly it calls the module graph_parser.R that generates the script dynamics.R containing the differential equations of the network dynamics.

The user can launch the software simply by running in RStudio the script network_analysis.R, and then by answering to the questions in the interactive interface as shown in Table 2.

**TABLE 2:** Interactive graphical user interface of NADS software showing the options and the task concerning the analysis of network topology.

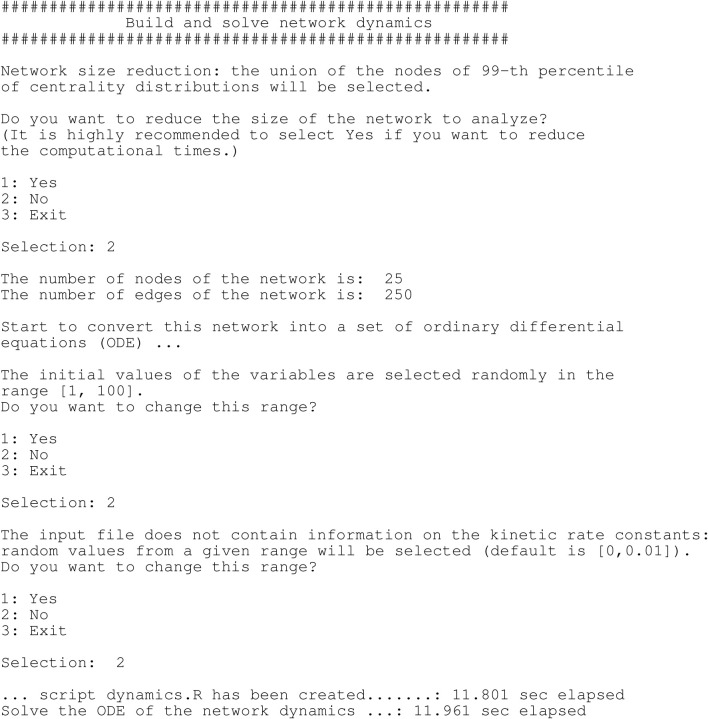

We provide also the fourth module implementing the parametric sensitivity analysis, named sensitivity_analysis.R, which takes as an input the system of equations automatically generated by network_analysis.R.

The main module is the script network_analysis.R. As soon as the user runs it from the R Studio GUI (R Studio, 2022) or from a terminal, an interactive output is displayed as in Tables 2, 3. The program asks the user to select the network to be analysed and then.

**FIGURE 2:**
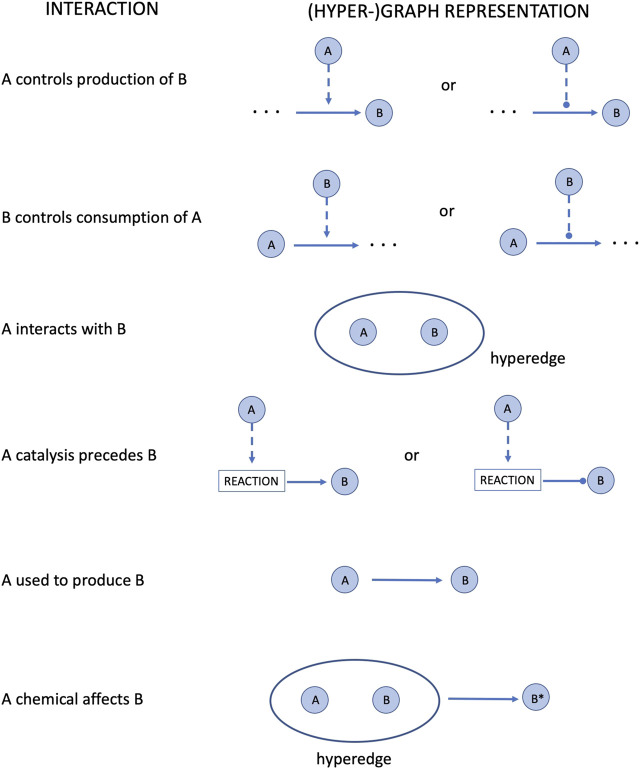
Conversion of the SIF format interactions into a (hyper-)graph structure.

• it calculates the centralities measures
• it translates the SIF network into a hyper-graph structure according to the rules reported in Figure 2
• then, it translates the hyper-graph into a set of ordinary differential equations, according to the rules reported in Table 3,
• and, finally, it solves them.

**TABLE 3:** Translation of BioPAX interactions into simple ordinary differential equations. See in Figure 2 the (hyper-)graph representation of these interactions.

Interaction	Differential equation
A controls production of B	$\frac{dB}{dt}=kA$ , $\frac{dA}{dt}=0$
B controls consumption of A	$\frac{dA}{dt}=-kB$ , $\frac{dB}{dt}=0$
A interacts with B	$\frac{dA}{dt}=\frac{dB}{dt}=-kAB$
A catalysis precedes B	$\frac{dB}{dt}=\pm kA$
A used to produce B	$\frac{dB}{dt}=kA$
A chemical affects B	$\frac{dA}{dt}=0$ , $\frac{dB}{dt}=-kB=-\frac{dB^{*}}{dt}$

The program returns also the execution times for the tasks expected to be the most computationally demanding, such as integrating the equation.

## 4 Results

**FIGURE 3:**
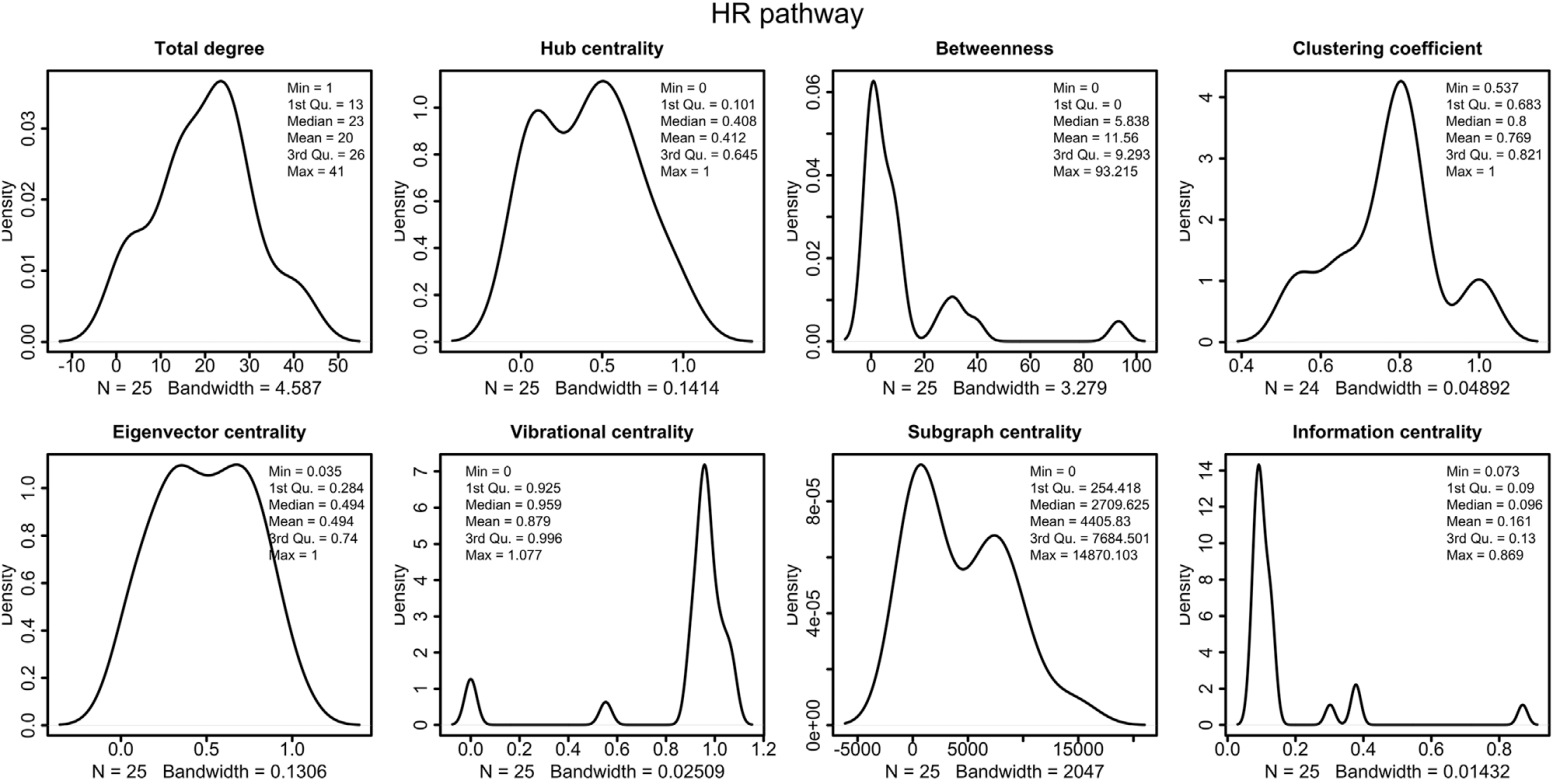
Distributions of the centrality measures of HR pathway (Orlic-Milacic, 2015). We observe that the majority of node have low betweenness, low information centrality and high vibrational centrality.

**FIGURE 4:**
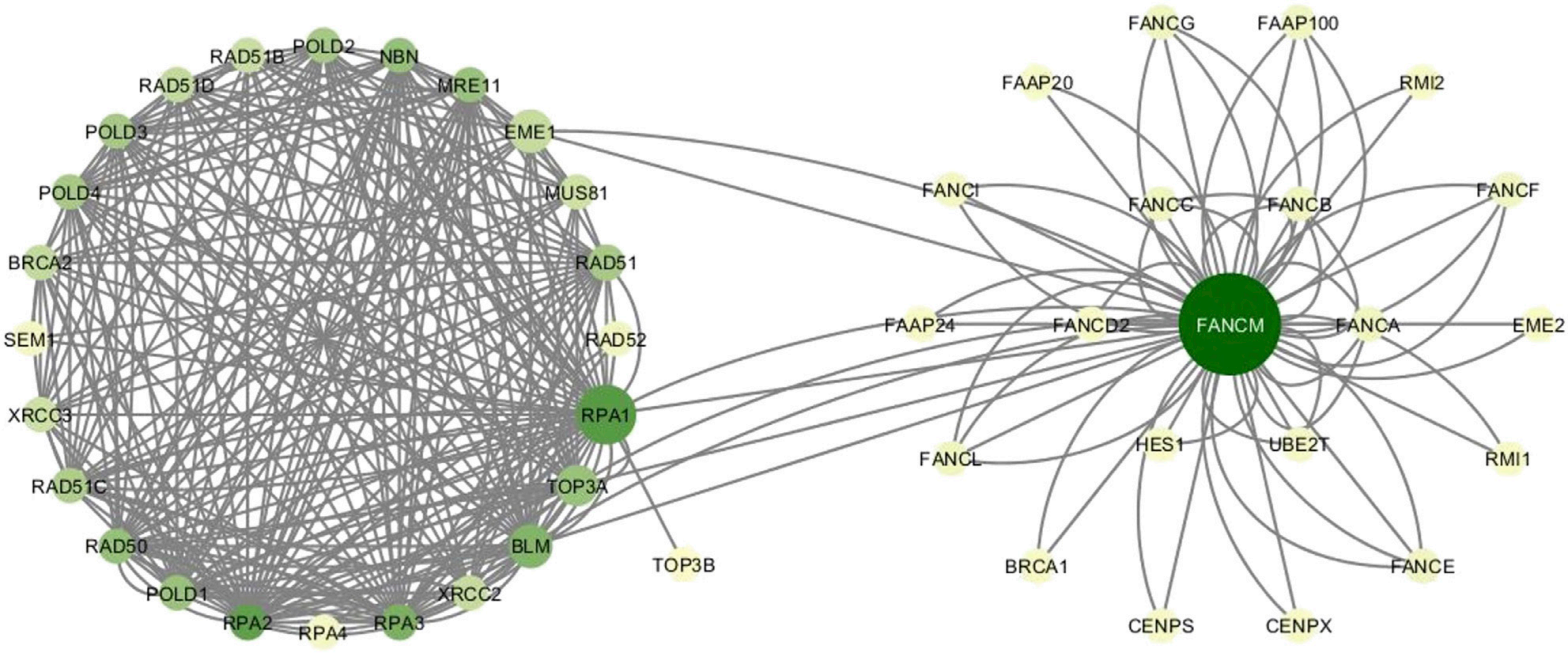
HR network and FANCM network. Colors vary from yellow to green according to increasing degree values. Node sizes grow as the betweenness centrality of nodes.

The HR network considered in this study has 25 nodes and 250 edges, as reported in Supplementary Tables S1–S3. The right part of the Figure 4 shows the HR network in circular layout. The network analysis phase of our study calculated the centrality measures distributions show in Figure 3, and identified six genes, as shown in Table 4:

1. BLM, scoring first for vibrational centrality
2. RAD50 scores first for sub-graph centrality
3. RAD52, scoring first for clustering coefficient
4. RPA1, scoring first for total degree and betweenness
5. RPA2, scoring first for in-degree and eigenvector centrality
6. RPA3, scoring first for out-degree, hub centrality, and sub-graph centrality
7. RPA4, scoring first for clustering coefficient
8. SEM1, scoring first for clustering coefficient.

**TABLE 4:** Values of the centrality measures for the HR pathway in (Orlic-Milacic, 2015). In bold, we marked the genes/proteins with the highest scores.

Protein	Total degree	In-degree	Out-degree	Hub centrality	Betweenness	Clustering coefficient	Eigenvector centrality	Vibrational centrality	Subgraph centrality	Information centrality
BLM	30	19	11	4.08E-01	39.5138622	0.2183908	0.81043641	**1.0771148**	7408.13084	0.08351073
EME1	13	0	13	5.21E-01	0	0.5384615	0.2589557	0.9579369	2709.62496	0.13185164
MRE11	26	16	10	3.69E-01	9.2926175	0.1969231	0.73968044	1.0587524	6020.65076	0.08957011
MUS81	13	1	12	5.18E-01	0	0.5384615	0.2589557	0.9547013	2291.00787	0.13177729
NBN	27	17	10	3.67E-01	9.2926175	0.1823362	0.76703983	1.0603542	6020.65076	0.08786049
POLD1	26	9	17	6.83E-01	7.8121197	0.36	0.67253587	0.9655278	7684.50086	0.08965382
POLD2	23	5	18	7.10E-01	5.8377525	0.4624506	0.57100865	0.9672104	9065.81813	0.09552092
POLD3	23	8	15	6.45E-01	5.8377525	0.4624506	0.57100865	0.961071	5521.89505	0.09524672
POLD4	23	7	16	6.66E-01	5.8377525	0.4624506	0.57100865	0.963361	6513.86378	0.09533952
**RAD50**	27	13	14	5.79E-01	10.2729789	0.2108262	0.75819149	1.0206621	**11427.9652**	0.08811688
RAD51	23	12	11	4.03E-01	30.3997662	0.4426877	0.49447273	0.9038147	1403.03278	0.09426237
RAD51B	14	12	2	8.58E-02	0.3636364	0.7032967	0.31841389	0.9960094	74.47586	0.12453711
RAD51C	21	9	12	4.96E-01	1.6614219	0.4095238	0.47619405	0.9027594	1911.88011	0.09955242
RAD51D	16	11	5	2.15E-01	0.5127787	0.7166667	0.3593928	0.9248884	282.05774	0.11524626
**RAD52**	4	2	2	1.01E-01	0	**1**	0.1247068	0.9567526	254.41809	0.30199153
**RPA1**	**41**	20	21	9.02E-01	**93.2152627**	0.1512195	0.89284007	0.9069973	8617.68902	0.0726257
**RPA2**	**41**	**22**	19	8.46E-01	32.6964097	0.1487805	**1**	0.9404821	8508.98841	0.07306925
**RPA3**	34	11	**23**	**1.00E+00**	25.979693	0.2174688	0.819185	0.9803911	**14870.10272**	0.07928594
**RPA4**	3	0	3	1.27E-01	0	**1**	0.10547581	0.9592952	700.52299	0.37822514
**SEM1**	3	1	2	9.14E-02	0	**1**	0.06803348	0.9398974	63.99639	0.37757664
TOP3A	24	12	12	4.64E-01	9.2926175	0.2318841	0.68062612	1.0251952	8739.44955	0.09349123
XRCC2	15	14	1	5.60E-02	0.8476272	0.7142857	0.34875334	1.0105203	55.0254	0.11916455
XRCC3	13	12	1	4.68E-02	0.3333333	0.8076923	0.2842042	0.5530372	0	0.13016119
BRCA2	16	16	0	1.40E-16	0	0.45	0.36198393	0	0	0.11465992
**TOP3B**	1	1	0	8.77E-18	0	NA	0.03472425	0	0	**0.86898249**

Of particular interest is the fact that BLM has the highest vibration centre. The interpretation of this result is that BLM is the node most sensitive to stresses and/or stimuli, i.e., according to the vibrational centrality measure, it is the most vulnerable node in the network (Estrada and Hatano, 2010). This result is of particular interest in light of the crucial role this gene plays in the HR network. Indeed, the key role of BLM is well know, and alterations in this protein is linked to different diseases including cancer (Kaur et al., 2021). BLM is a 3′-5′ ATP-dependent RecQ DNA helicase. It is a genome stabilizer playing an essential role in the DNA replication regulation, DNA recombination, and both homologous and non-homologous pathways of DSB repair. The high vulnerability of the BLM node to external stimuli and conditions suggests the need to identify which conditions and/or stimuli may be altering it, in order to preserve its proper functioning and/or to understand how it can be restored if it is altered. The high vulnerability of this node could also be explained by a recent study by Kaur et al. (2021). These authors report that BLM has a dual function both as a tumour suppressor and possibly as a proto-oncogene, being probably involved in the mechanisms of its deregulation in tumours.

The analysis also correctly identifies the SEM1 gene as a node with a high clustering coefficient. Indeed, as reported in (Safran et al., 2021; GeneCard, 2022), SEM1 gene encodes for a protein that is part of a 26S proteasome, which is a multiprotein complex with a function in the ATP-dependent degradation of ubiquitinated proteins. This complex contributes to the maintenance of protein homeostasis by removing misfolded or damaged proteins, which could jeopardize the healthy cellular functions, and by removing proteins no longer need. Therefore, 26S proteasome is involved in numerous cellular processes, including cell cycle progression, apoptosis, or DNA damage repair (Sone et al., 2004).

SEM1 was found also as a subunit in experiments of affinity purification of the yeast 19S proteasome, and its human homolog, DSS1, was found to copurify with the human 19S proteasome (Krogan et al., 2004). DSS1 is associated with the tumour suppressor protein BRCA2 involved in DNA DSBs repair. The authors in (Krogan et al., 2004) proved that SEM1 is essential for efficient repair of an HO-generated yeast DSB using both HR and nonhomologous end joining (NHEJ) pathways. Moreover, they showed that deletion of SEM1 contributes to cause defects in (synthetic) growth and hypersensitivity to genotoxins when combined with mutations in certain well-established genes involved in the DNA DSB repair.

Similarly to SEM1, the result of a high clustering coefficient is also expected for RPA4, as RPA4 is also part of a complex (Keshav et al., 1995). RPA4 gene encodes a single-stranded DNA-binding protein that is a subunit of the replication protein A complex (GeneCard, 2022). Replication protein A is essential for DNA DSB repair and plays a crucial role in the activation of cell cycle checkpoints. As regards the RPA complex, we have already seen in the previous sections that the RPA complex controls DNA repair and DNA damage checkpoint activation as well. In particular, the network analysis shows that RPA1 highly scores by total degree and betweenness. These results reflect the fact that RPA1 is an active route of communication exchanges between various nodes in the network. RPA1 is part of the heterotrimeric replication protein A complex (RPA/RP-A). It stabilizes single-stranded DNA intermediates, that form during DNA replication or upon DNA stress In (Bass et al., 2016; Haahr et al., 2016; Human Protein Atlas, 2022). It prevents the reannealing of single-stranded DNA intermediates and recruits and activates different proteins and complexes forming part of DNA metabolism. Thereby, it is a key protein both in DNA replication and in the cellular response to DNA damage (Lin et al., 1998). RPA2 shows high score for in-degree and eigenvevtor centrality, meaning that it is interacting with protein also highly scoring by eigenvector centrality and degree (Hansen et al., 2020), and thence with proteins which have a great influence in the HR network. Indeed RPA 2 gene has been found highly expressed in low grade carcinomas and its expression has a gradual significant decrease from stage I to stage IV carcinomas. All the three subunits RPA1, RPA2, and RPA3, were more abundant (with statistical significance evidence) in lymph node negative and earlier stage (stage I and II) gastric carcinomas (Fourtziala et al., 2020). Finally, of particular interest and the fact that RPA3 ranks first in terms of centrality out-degree, hub-centrality and sub-graph centrality. Since subgraph centrality of a node is the number of subgraphs a node participates in (weighted according to their size) (Estrada and Rodríguez-Velázquez, 2005), it means that RPA3 take part into a number of subgraphs of significant size relatively to the whole network size. From our analysis it results that RPA3 versus RPA1 and RPA2, although its roles are similar to those of RPA2, has thus a great influence on pathways of crucial importance more than on subset of unconnected nodes or single nodes.

The analysis also highlights RAD50 that is a component of the MRN complex The protein complex is involved in numerous enzymatic activities required for nonhomologous joining of DNA ends. It is protein is essential for DNA double-strand break repair, cell cycle checkpoint activation, telomere maintenance, and meiotic recombination. (Carney et al., 1998; de Jager et al., 2001; Estrada and Ross, 2018; Bian et al., 2019; Beikzadeh and Latham, 2021; National library of Medicine, 2022). This role is reflected by th ehigh sungraph centrality that measures the centrality of a node by taking into account the number of subgraphs the node participates in. Specifically, the subgraph centrality of a node is the number of closed loops originating at the node, where longer loops are exponentially downweighted. Consequently, subgraph centrality measures how close a node is to the other nodes in the network.

The results of this analysis reveal a correspondence between the measure of centrality and the role of the protein. On the basis of this, when the role of the protein is known, this information can be used to work out the correctness of the computational analysis. When, on the other hand, the role of the protein is not known, knowledge of its centrality measurements can suggest the type or set of types of possible roles.

In Tables 5, 6 we show the rate equations of the HR network dynamics generated as the automatic translation of the network (see the script dynamics.R in the GitLab repository of NADS software). In file Simulations_of_Dynamics_HR_pathway.pdf provided in the Supplementary Material, we show the time evolution curves of each node of the HR network obtained as a solution of the equations.

**TABLE 5:** This is the PART I of the table of ordinary differential equations of the dynamics of HR network, in R code formalism. The *k* followed by a number denote the kinetic rate constant, and the letter “d” in front of the name of the proteins denote the temporal derivative of it concentration.

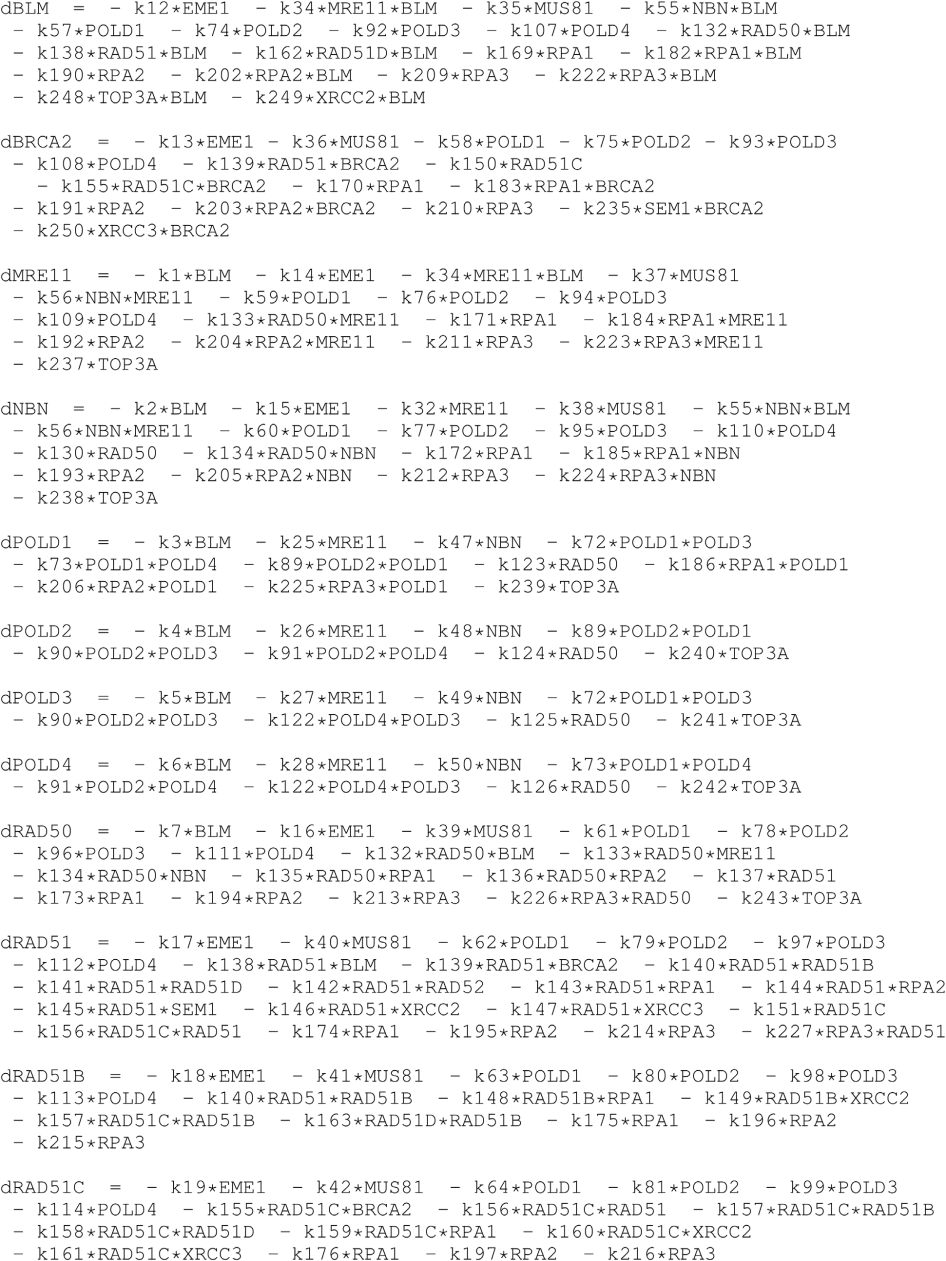

**TABLE 6:** This is the PART II (continuation) of the table of ordinary differential equations of the dynamics of HR network, in R code formalism. The *k* followed by a number denote the kinetic rate constant, and the letter “d” in front of the name of the proteins denote the temporal derivative of it concentration.

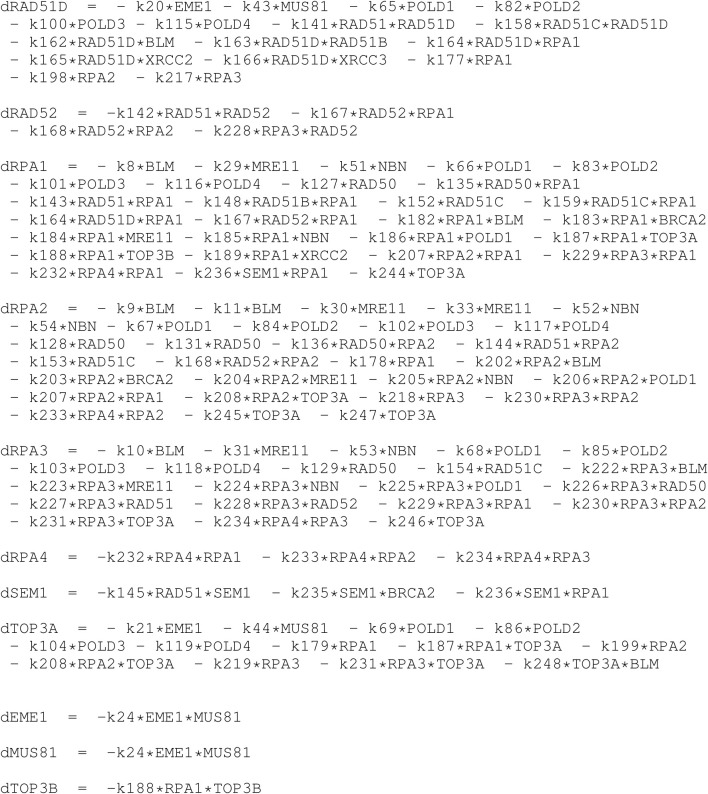

**FIGURE 5:**
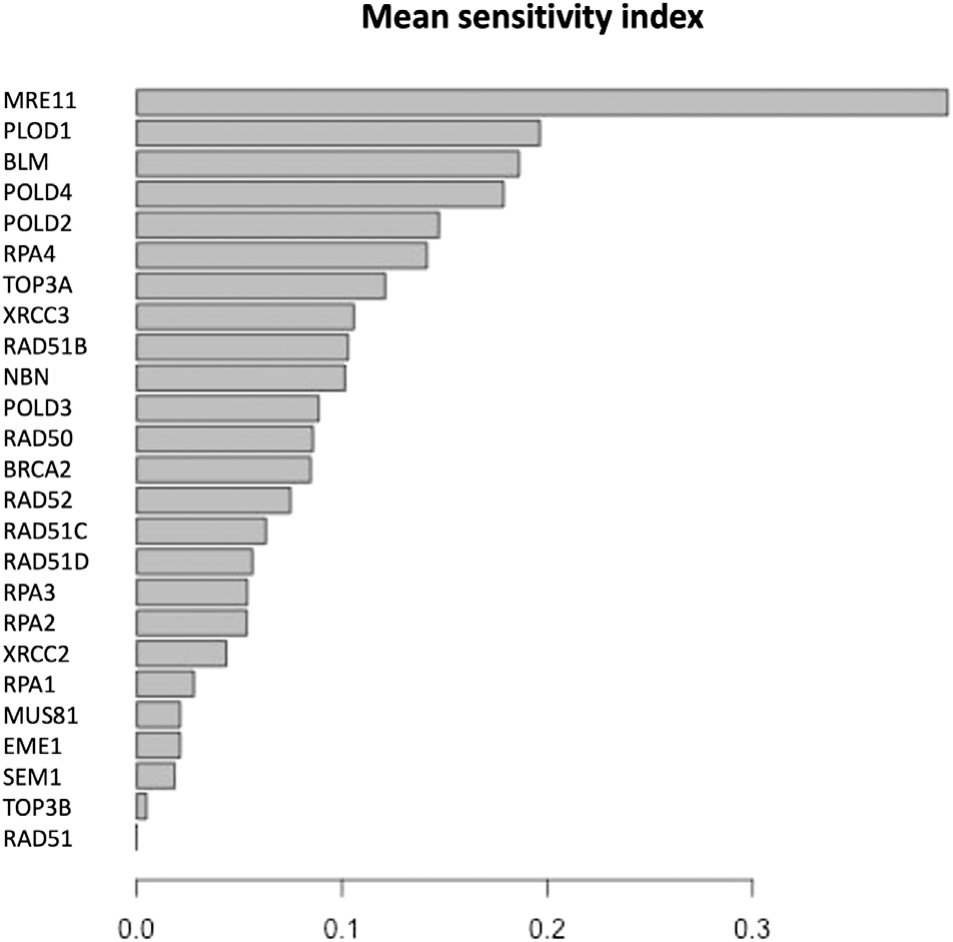
Mean of the sensitivity index distributions for the proteins in HR network (Orlic-Milacic, 2015). These results refer to simulation in the time interval [0, 10] a.u., and initial values of the proteins randomly sampled in the range [1, 100] a. u. and kinetics rates values sampled in the interval [0, 0.01].

**FIGURE 6:**
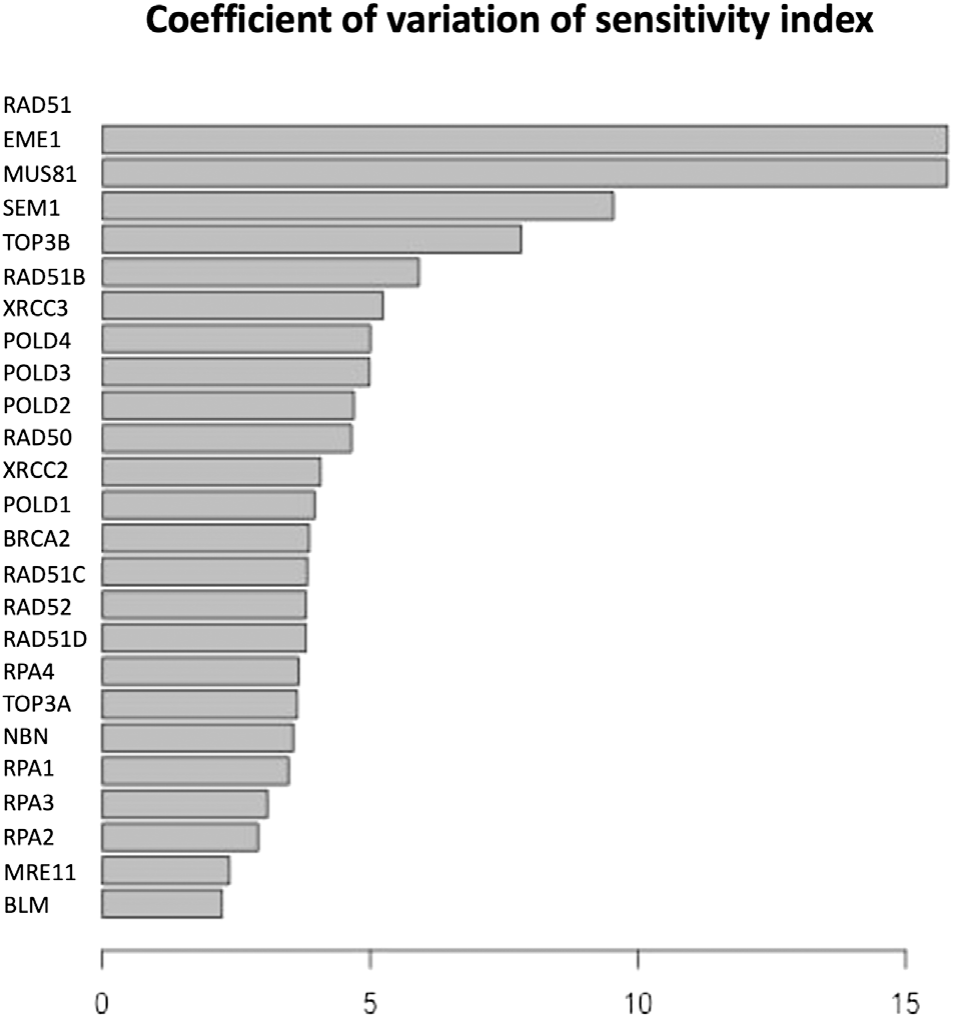
Coefficient of variation of the sensitivity index distributions for the proteins in HR network Orlic-Milacic, (2015). These results refer to simulation in the time interval [0, 10] a.u., and initial values of the proteins randomly sampled in the range [1, 100] a. u. and kinetics rates values sampled in the interval [0, 0.01].

The parameter sensitivity analysis was conducted by perturbing each parameter in the convergence range of the solution and yielded the results shown in Table 7; Figures 5, 6. In Table 7 we report the kinetic rate constants for which the sensitivity index belongs ot the 98th percentile of the sensitivity index distribution. They correspond to the most sensitive parameters, i.e. to the interactions whose alterations can significantly alter the dynamics of the network. To find out which interactions they refer to, the reader can refer to the Supplementary Tables S1–S3. Figure 5 we shows that MRE11, followed by POLD1, BLM has the highest average sensitivity index. In Figure 6 we show the coefficient of variation of each protein in the HR network. The coefficient of variation, being the ratio of the standard deviation to the mean, measures the extent of variability in relation to the mean of the population. The higher the coefficient of variation, the greater the dispersion. BLM, followed by MRE11 and RPA2 exhibits the lowest coefficient of variation of the sensitivity index. This mean that BLM, MRE11 and RPA2 have high sensitivity indices, and that the distribution of the sensitivity indices is well shaped around its mean, i.e. these protein exhibit almost the same sensitivity for all the parameters of the model. In this study we have therefore found that the BLM and RPA2 are sensitive nodes and that their sensitivity has two components: a topological sensitivity expressed by vibrational centrality, eigenvector centrality and clustering coefficient, and a dynamic sensitivity expressed by the parameter sensitivity index.

**TABLE 7:** For each gene/protein in the HR pathway in (Orlic-Milacic, 2015) we selected the kinetic rates whose sensitivity index belongs to the 98th percentile of the sensitivity index distribution. The sensitity index is calculated using the formula (1).

Gene	Kinetic rate to which it is highly sensitive
BLM	k12, k34, k107, k138, k222
BRCA2	k13, k58, k108, k139, k250
EME1	k24, k138, k140, k144, k227
MRE11	k14, k34, k109, k204, k223
MUS81	k24, k138, k140, k144, k227
NBN	k15, k32, k34, k60, k110
POLD1	k72, k73, k89, k90, k91
POLD2	k72, k73, k89, k90, k91
POLD3	k72, k89, k90, k91, k122
POLD4	k73, k89, k90, k91, k122
RAD50	k16, k61, k78, k111, k137
RAD51	–
RAD51B	k18, k63, k113, k140, k163
RAD51C	k19, k64, k81, k114, k157
RAD51D	k20, k65, k115, k141, k162
RAD52	k142, k168, k222, k227, k228
RPA1	k8, k29, k66, k83, k116
RPA2	k30, k33, k117, k144, k206
RPA3	k31, k68, k118, k222, k227
RPA4	k29, k227, k232, k233, k234
SEM1	k139, k145, k235, k236, k250
TOP3A	k21, k69, k86, k119, k248
TOP3B	k29, k66, k83, k116, k188
XRCC2	k22, k70, k120, k146, k149
XRCC3	k23, k121, k147, k166, k250

As with the BLM and RPA2 proteins, sensitivity analysis also highlights the MRE11 protein, which is highly sensitive to kinetic parameters, and its vibrational centrality is 1.0587524, very close to the maximum value exhibited by BLM (see Table 4). Its eigenvector centrality is 0.7397 which, although not the maximum, is very close to it (see Table 4). Indeed, MRE11 is an integral part of the protein complex of RAD50-MRE11A-NBS1 known as the MRN complex (Porras, 2014; Shibata et al., 2014; Mukherjee et al., 2019). It plays a key role in homologous recombination, and it is generally believed that MRE11 initiates double-strand breaks resection. In particular, the authors show that the loss of MRE11 reduces the efficiency of homologous recombination in human TK6 cells without affecting double-strand breaks resection, indicating a role for MRE11 in homologous recombination also at a post-resection step.

The high value of the eigenvector centrality fork BLM, RPA2 and MRE11 confirms the crucial role of these proteins in the network and expresses the fact that they are pointed by nodes that have a high value of the eigenvector centrality too. Indeed, if a node is pointed to by many nodes (which also have high eigenvector centrality) then that node will have high eigenvector centrality (Fletcher and Wennekers, 2018). The high sensitivity to the parameters characterising the dynamics of the interactions between these proteins and the partners pointing to them indicates the great influence that these partner nodes have on these proteins. Interestingly, RAD51 does not result sensitive to any parameter. The RAD51 encodes a protein that is essential for repairing damaged DNA. Recent findings have indicated RAD51 protein is overexpressed in a variety of tumours Chen et al. (2017). The overexpression of RAD51 causes improper and hyper-recombination, and thus contributes to genomic instability and genetic diversity. Genomic instability might, in turn, drive regular cells towards neoplastic transformation or further contributes to cancer metastatic progression (Chen et al., 2007). The RAD51 protein binds to the DNA at the site of a break and encapsulates it in a protein sheath, initiating the repair process MedlinePlus (2022); Uniprot (2022). RAD51 protein interacts with BRCA1 and BRCA2, to fix damaged DNA. The BRCA2 protein regulates the activity of the RAD51 protein by transporting it in the nucleus to sites of DNA damage. Although the interaction between the BRCA1 protein and the RAD51 protein has still to be elucidate, research suggests that BRCA1 may also activate RAD51 in response to DNA damage (Cousineau et al., 2005; Chappell et al., 2016). The result of the sensitivity analysis found seems to contradict the important role of this protein in these interactions. Indeed, for example, one might expect a high sensitivity of RAD51 to the k139 due to its interaction with BRCA2 (see Supplementary Table S2). One explanation for this contradiction could be that since the mechanisms of interaction of RAD51 with these proteins are not fully known, the model used in this study could be an oversimplification of the interaction of RAD51 with its partners. If more accurate models in the future confirm the low sensitivity of RAD51 to the parameters of the rate equations describing the dynamics of the network, it will be necessary to investigate the physical and biological characteristics that make it so stable to perturbations. The fact that RAD51 has a low value of vibrational centrality in this study is a factor in favour of the possible confirmation of this case.

**FIGURE 7:**
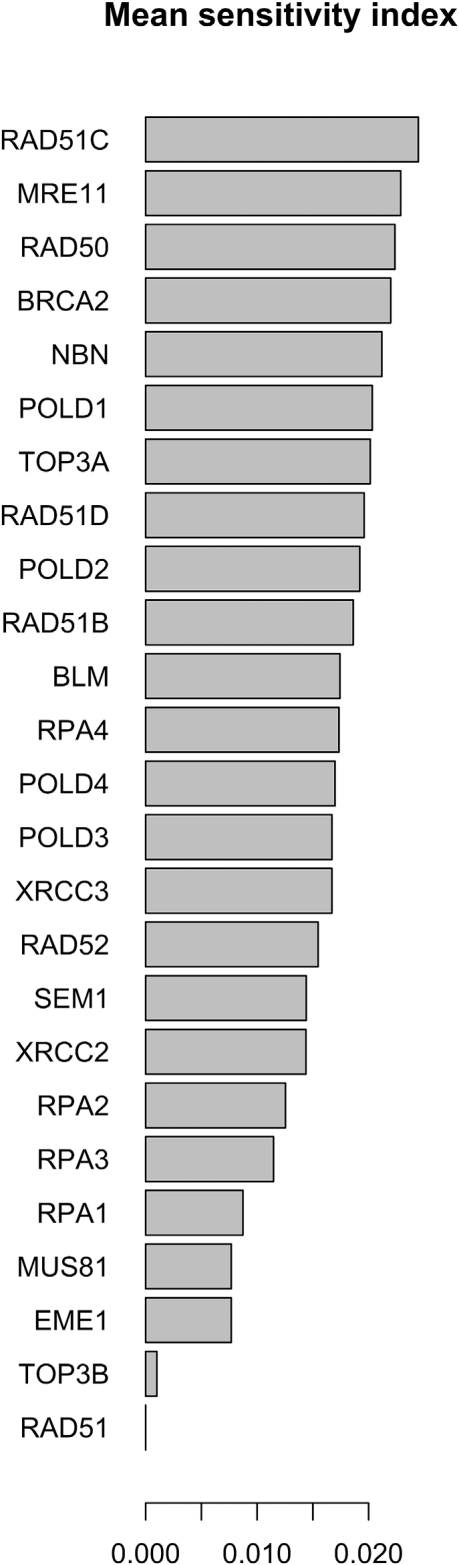
Mean of the sensitivity index distributions for the proteins in HR network Orlic-Milacic, (2015). These results refer to simulation in the time interval [0, 1400] a.u., and initial values of the proteins randomly sampled in the range [18, 20] a. u. and kinetics rate values sample in [10^–5^, 10^–6^] a. u.

**FIGURE 8:**
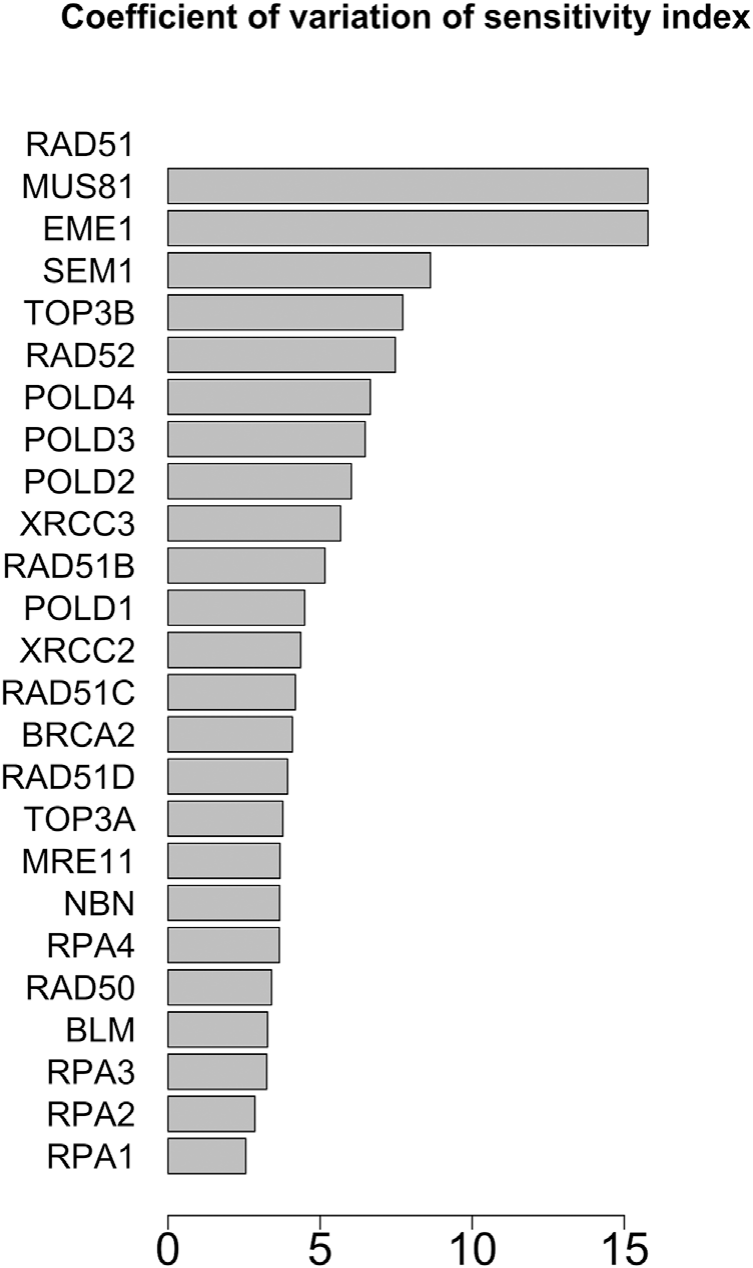
Coefficient of variation of the sensitivity index distributions for the proteins in HR network (Orlic-Milacic, 2015). These results refer to simulation in the time interval [18, 20] a.u., and initial values of the proteins randomly sampled in the range [10^–5^, 10^–6^] a. u.

In Figures 7, 8 we report the results of the sensitivity analysis obtained selecting different ranges of initial conditions and parameters values. The plots highlights RAD51C, MRE11, RAD50 and BRCA2 as the most sensitive nodes to the parameters. We comment in the Section Remarks the expected differences and similarities in the results of sensitivity analysis when we change the intervals of initial conditions and parameters.

### 4.1 Analysis of the HR Pathway Merged With FANCM Pathway

We repeated the analysis on the HR’s network extended by adding the pathways of FANCM gene (Fanconi Anaemia Group M Protein), obtained from Pathways Commons (Pathways Commons, 2022) given its important role in genome duplication, repair mechanisms and its involvement in the development of Fanconi anaemia, which several studies report to be a syndrome related to cancer predisposition (Deans and West, 2009; Xue et al., 2015; Bhattacharjee and Nandi, 2017; Pan et al., 2017; Wang et al., 2018). Finally, a recent study of Panday et al. reports that FANCM regulates repair pathway choice at stalled replication forks (Ling et al., 2016; Panday et al., 2021). FANCM and BLM have a similar role and cooperatively act in the DNA repair mechanisms (Panday et al., 2021), and through this analysis we want to investigate on this similarity.

The new network including HR and FANCM pathways is made up of 46 nodes and 316 edges. The left part of Figure 4 shows the FANCM pathway and its connection with HR network. As reported in Table 8, the calculation of centrality measures led to the following results:

• BLM has the highest score in vibrational centrality
• FANCM has the highest score in total degree, in degree and betweenness
• RAD50 scores first for subgraph centrality
• RAD52 scores first for clustering coefficient
• RPA1 scores first for out-degree
• RPA2 scores first for eigenvector centrality
• RPA3 scores first for hub centrality
• RPA4 scores first for clustering coefficient
• SEM1 scores first for clustering coefficient.

**TABLE 8:** Values of the centrality measures for the HR pathway in (Orlic-Milacic, 2015) merged with the FANCM pathway in (Pathways Commons, 2022). In bold, we marked the genes with the highest scores.

Protein	Total degree	In-degree	Out-degree	Hub centrality	Betweenness	Clustering coefficient	Eigenvector centrality	Vibrational centrality	Subgraph centrality	Information centrality
BLM	32	19	13	4.83E-01	117.0805289	0.72058824	0.83056114	**1.107752**	7408.39241	0.1204394
EME1	15	0	15	5.92E-01	0	0.48351648	0.28101096	9.68E-01	2727.280253	0.1730775
MRE11	26	16	10	3.71E-01	9.2926175	0.82051282	0.73943396	1.08E+00	6025.93013	0.1319644
MUS81	13	1	12	5.18E-01	0	0.53846154	0.25936455	9.56E-01	2292.062046	0.1868928
NBN	27	17	10	3.69E-01	9.2926175	0.82051282	0.76658977	1.08E+00	6025.93013	0.1296602
POLD1	26	9	17	6.82E-01	7.8121197	0.76470588	0.67199489	9.86E-01	7688.630996	0.1322165
POLD2	23	5	18	7.10E-01	5.8377525	0.76470588	0.57047643	9.81E-01	9071.417868	0.1400556
POLD3	23	8	15	6.44E-01	5.8377525	0.76470588	0.57047643	9.78E-01	5524.141817	0.1396592
POLD4	23	7	16	6.65E-01	5.8377525	0.76470588	0.57047643	9.80E-01	6516.909994	0.1397968
**RAD50**	27	13	14	5.81E-01	10.2729789	0.81318681	0.75778635	1.04E+00	**11437.382893**	0.1300547
RAD51	23	12	11	4.02E-01	30.3997662	0.65497076	0.4937734	9.17E-01	1403.106256	0.1386277
RAD51B	14	12	2	8.53E-02	0.3636364	0.82051282	0.31833841	1.01E+00	74.55065	0.1774293
RAD51C	21	9	12	4.93E-01	1.6614219	0.81904762	0.47562207	9.12E-01	1911.911354	0.1453357
RAD51D	16	11	5	2.13E-01	0.5127787	0.81904762	0.3597479	9.38E-01	282.219746	0.1656386
RAD52	4	2	2	1.00E-01	0	**1**	0.12449815	9.63E-01	254.418337	0.3917785
**RPA1**	43	20	**23**	9.74E-01	215.4152627	0.49802372	0.91307865	9.47E-01	8620.183617	0.1074375
**RPA2**	41	22	19	8.45E-01	32.6964097	0.64210526	**1**	9.82E-01	8511.793141	0.1095086
**RPA3**	34	11	23	**1**	25.979693	0.64210526	0.81828849	9.98E-01	14877.81448	0.1182323
**RPA4**	3	0	3	1.27E-01	0	**1**	0.10543731	9.63E-01	700.745966	0.4815787
**SEM1**	3	1	2	9.07E-02	0	**1**	0.0682632	9.28E-01	64.063815	0.4810717
**TOP3A**	26	12	14	5.39E-01	43.5259509	0.73626374	0.70171805	1.04E+00	8739.804106	0.1322725
XRCC2	15	14	1	5.59E-02	0.8476272	0.82417582	0.3492084	1.03E+00	55.032467	0.1704502
XRCC3	13	12	1	4.65E-02	0.3333333	0.80769231	0.28356645	5.52E-01	0	0.1844967
BRCA1	2	0	2	7.17E-02	0	NA	0.02248201	9.53E-01	15.252907	0.6489791
CENPS	2	0	2	7.17E-02	0	NA	0.02248201	9.53E-01	15.252907	0.6489791
CENPX	2	0	2	7.17E-02	0	NA	0.02248201	9.53E-01	15.252907	0.6489791
EME2	2	0	2	7.17E-02	0	NA	0.02248201	9.53E-01	15.252907	0.6489791
FAAP100	4	2	2	7.17E-02	0	NA	0.04496402	1.00E+00	14.252907	0.3943335
FAAP20	2	0	2	7.17E-02	0	NA	0.02248201	9.53E-01	15.252907	0.6489791
FAAP24	2	0	2	7.17E-02	0	NA	0.02248201	9.53E-01	15.252907	0.6489791
FANCA	4	2	2	7.17E-02	0	NA	0.04496402	1.00E+00	14.252907	0.3943335
FANCB	4	2	2	7.17E-02	0	NA	0.04496402	1.00E+00	14.252907	0.3943335
FANCC	4	2	2	7.17E-02	0	NA	0.04496402	1.00E+00	14.252907	0.3943335
FANCD2	2	1	1	3.59E-02	0	NA	0.02248201	9.77E-01	3.563227	0.6504898
FANCE	4	2	2	7.17E-02	0	NA	0.04496402	1.00E+00	14.252907	0.3943335
FANCF	4	2	2	7.17E-02	0	NA	0.04496402	1.00E+00	14.252907	0.3943335
FANCG	4	2	2	7.17E-02	0	NA	0.04496402	1.00E+00	14.252907	0.3943335
FANCI	3	1	2	7.17E-02	0	NA	0.03372301	9.77E-01	14.252907	0.4827827
FANCL	4	2	2	7.17E-02	0	NA	0.04496402	1.00E+00	14.252907	0.3943335
**FANCM**	**66**	**44**	22	4.35E-16	**481**	0.01811594	0.29119966	5.13E-01	131.839388	0.0955692
RMI1	2	0	2	7.17E-02	0	NA	0.02248201	9.53E-01	15.252907	0.6489791
RMI2	2	0	2	7.17E-02	0	NA	0.02248201	9.53E-01	15.252907	0.6489791
UBE2T	3	2	1	3.59E-02	0	NA	0.03372301	1.00E+00	3.563227	0.4815848
BRCA2	16	16	0	1.09E-16	0	0.69230769	0.36151501	-4.08E-18	0	0.1645300
TOP3B	1	1	0	6.80E-18	0	NA	0.03524702	0.00E+00	0	**1.1169817**
HES1	2	2	0	1.36E-17	0	NA	0.02248201	0.00E+00	0	0.6438225
MAX SCORE	43	22	23	0.9743705	215.4152627	0.82417582	0.91307865	1.08361	14877.81448	0

**FIGURE 9:**
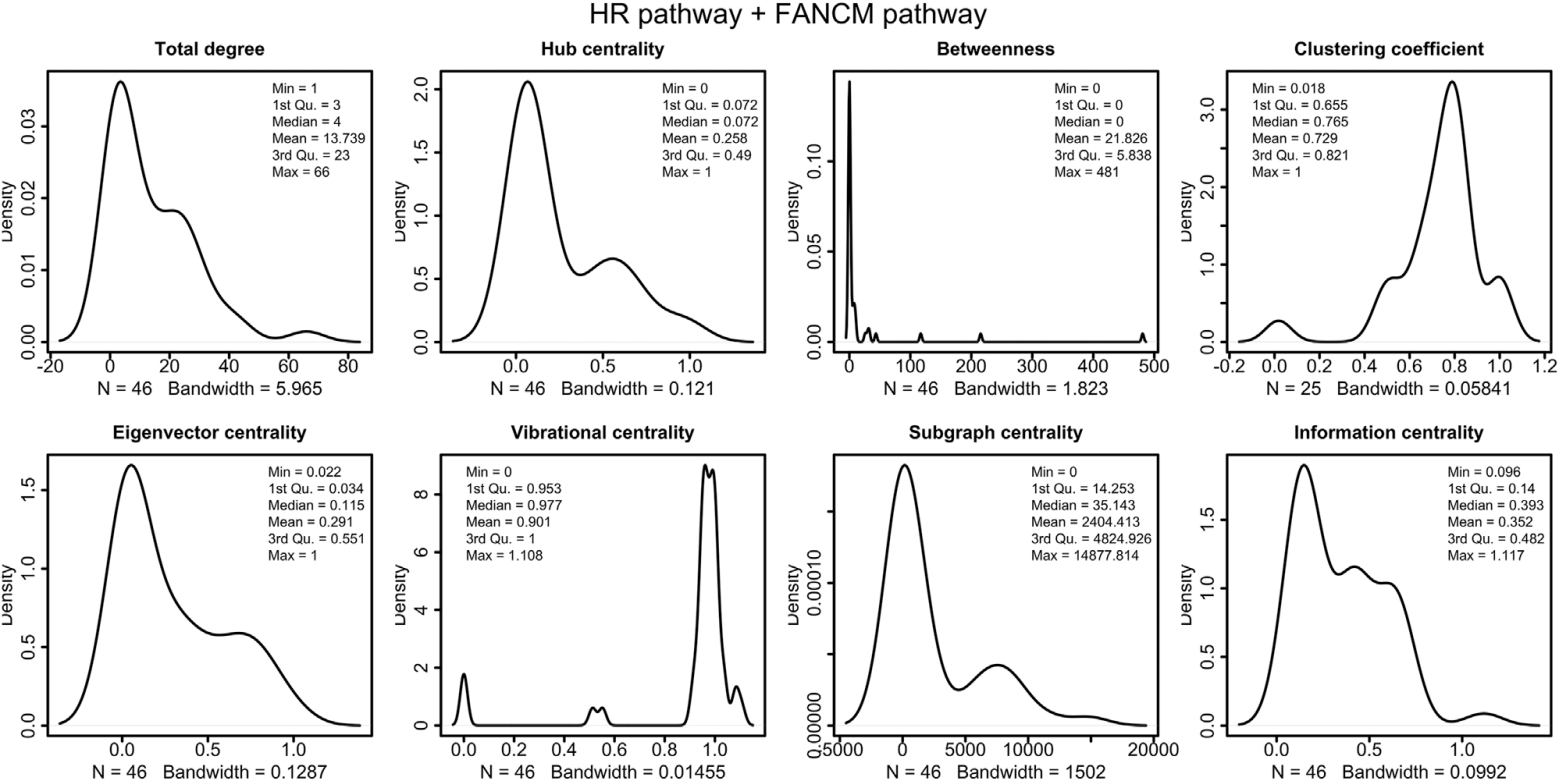
Distributions of the centrality measures of HR pathway (Orlic-Milacic, 2015) merged with FANCM pathway (Pathways Commons, 2022). We observe that the majority of node have low betweenness, and high vibrational centrality.

**FIGURE 10:**
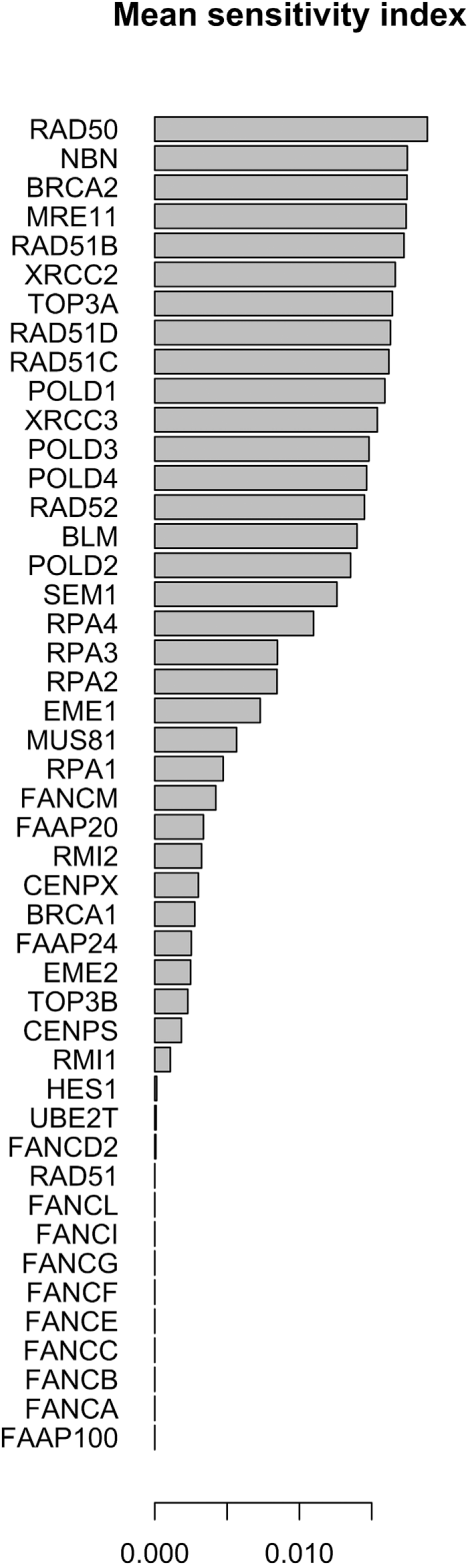
Mean of the sensitivity index distributions for the proteins in HR network Orlic-Milacic, (2015) merged with FANCM pathway (Pathways Commons, 2022). These results refer to simulation in the time interval [0, 1400] a.u., and initial values of the proteins randomly sampled in the range [18, 20] a. u. and kinetics rate values sample in [10^–5^, 10^–6^] a. u.

**FIGURE 11:**
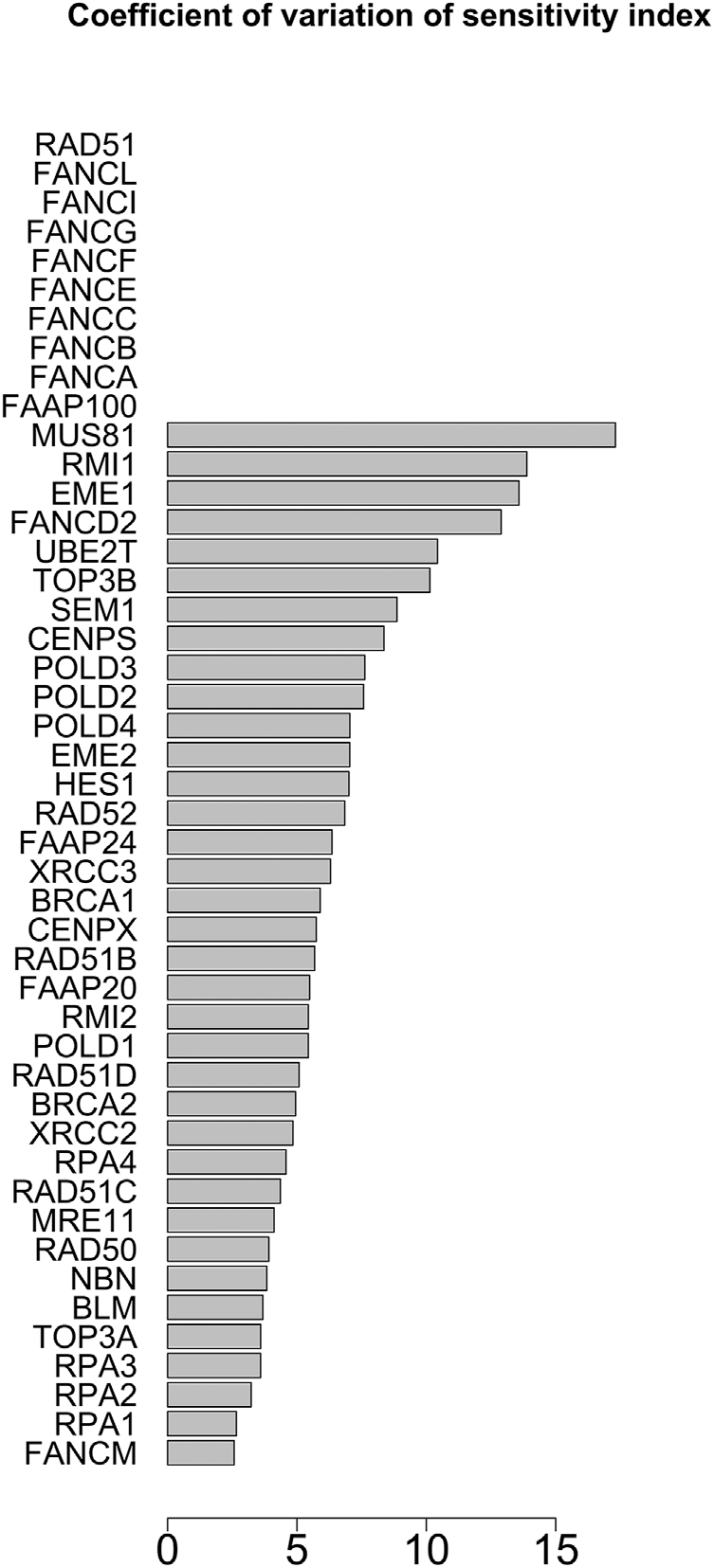
Coefficient of variation of the sensitivity index distributions for the proteins in HR network (Orlic-Milacic, 2015) merged with FANCM pathway (Pathways Commons, 2022). These results refer to simulation in the time interval [18, 20] a.u., and initial values of the proteins randomly sampled in the range [10^–5^, 10^–6^] a. u.

The distribution of the centrality measures on the entire network is shown in Figure 9. These results not only re-emphasise as central the genes/proteins already identified in the analysis of the HR network alone in (Orlic-Milacic, 2015), but also highlight the central role of FANCM and as a node of particular relevance due to their high in-degree and high betweenness. By assigning an initial quantity between 18 and 20 (expressed in arbitrary units) to each node, the solution of the system of 46 differential equations converges for values of rate constants in the range 10^–4^ and 10^–5^ a.u. In file Simulations_of_Dynamics_HR_FANCM_pathway.pdf provided in the Supplementary Material, we show the time evolution curves (obtained as a solution of the equations) of each node of the HR pathway merged with FANCM pathway. The parameter sensitivity analysis was conducted by perturbing each parameter in the convergence range of the solution and yielded the results shown in Figures 10, 11. We found that the nodes most sensitive to the parameters are RAD50, NBN, BRCA2, MRE11 and RAD51B. Compared to what was obtained in the analysis of the HR network alone, we find here that BLM is no longer at the top in terms of parameter sensitivity while still maintaining a central role in terms of vibration centrality.

### 4.2 Remarks

The method provides a range of values for the rate constants within which the solution to the problem exists at the given set of initial values for the node concentration/abundance. We can interpret this range as that of the ‘most probable’ range of values if.

• the initial conditions are known
• the analysed network does not exclude important interactions occurring *in vivo* and if the system is subjected to the conditions of the real system *in vitro* and *in vivo*. The network considered is only an extract of a much more complex network (still not completely known) that operates *in vivo* and in interaction with environmental factors.

We also note that we do not dispose of experimental time curves that can be used to calibrate the model. Calibration from experimental data rather than sensitivity analysis would be the most appropriate method to use to obtain an estimated (even interval) parameter estimate. In the absence of both experimental data. We agree with the Reviewer that sensitivity analysis provides information on the minimum set of parameters to be inferred from experimental data, since the parameters to which the model is less sensitive are less influential.

Finally, we also observe that having fixed a set of initial values for the concentrations/abundances of the network components, more than one set of intervals for the rate constants could guarantee the convergence of the numerical method of solving the system of differential equations. It is also true that by changing the initial values of the concentrations, the range of values of the rate constants for which the system converges could change. The results that we report in this new version of the manuscript show, for example, that if the range of the initial concentration values is between 0 and 100, the numerical solution is found for rate constant values between 0 and 0.01, whereas if the range of the initial concentration values is a few tens, the numerical solution is found for rate constant values between 10^–6^ and 106–5. A reduction of 10 in the order of magnitude of the initial concentration values thus corresponds to a reduction of 10^–3^ in the order of magnitude of the rate constants. This is an indication that the system is underdetermined, and in fact consists of more parameters than the number of variables and in the complete absence of experimental data. All this also shows that calibrating the model in the light of experimental data is the best way to hope for a set of ‘probable’ values of the rate constants.

The work shown in this study therefore does not so much emphasise the numerical solutions, but, through a mathematical model, wants to test the susceptibility of the network components to the parameters and wants to integrate it with the role that the network components have (estimated by the centrality measurements).

## 5 Conclusion

This report presented an application of network analysis and mathematical modelling to the double-strand break repair pathway homologous recombination repair (HR). The complexity of the network of repair mechanisms itself, as well as the complexity of its interactions with the surrounding environment (Li et al., 2009; Chatterjee and Walker, 2017; Kusakabe et al., 2019; Poetsch, 2020; Roux et al., 2021), and the mutations of its components make its mathematical modelling particularly difficult, especially when based on rate equations. It is therefore of great necessity to have a tool that can implement these two important steps:

1. network analysis including standard centrality measures and new measures to quantify the robustness and responsiveness of the network to stimuli and stresses not dependent on the network topology
2. automatic construction of a mathematical model, for its analysis, and which allows to carry out refinements and modifications, when new data and new experimental knowledge make it necessary.

The implementation of these step is an innovative perspective for the analysis of DNA repair mechanisms. So far in the literature, there are many studies and analyses focused on the genetic and genomic aspects of the pathways, but studies on the mathematical modelling of its dynamics are absent. Our study therefore aims to fill this gap, since the mechanisms of DNA repair are governed by genes, proteins and pathways in continuous communication with the environment. For this reason, the analysis of the dynamics of the network is particularly useful, since it can quantify the vulnerability of the network and the modes of response to stimuli and exogenous stress. To the best of our knowledge there are no schemes for translating a graph associated with a biological network into a set of dynamic equations. The main reason for this is that there is no unambiguously defined semantics of a graphic representation of a biological network, i.e. there is no unambiguous definition of the graphic symbolism in terms of the mathematical equation describing the interaction indicated by that symbolism. The translation model we propose in this study is a basic model that describes the interactions indicated by the graph with linear first-order differential equations in the parameters. The code that implements this translation, however, gives the user the possibility to modify the model where he/she deems it appropriate in the light of available biological knowledge, or in the case he/she like to generate new hypothetical scenarios. We believe that the availability of a tool such as NADS can support the investigation of such a complex network that is subject to continuous interaction with external agents, not only to understand its dynamics, but also to predict its evolution and identify points of vulnerability for the benefit of the medical applications that this research may provide.

## Data Availability

The original contributions presented in the study are included in the article/Supplementary Material, further inquiries can be directed to the corresponding authors. the software used int eh analysis is available at https://gitlab.inf.unibz.it/Paola.Lecca/network-analyzer-and-dynamics-simulator.
