# Supplementary material for: Dynamic Modelling of DNA Repair Pathway at the Molecular Level: A New Perspective
Source: Front Mol Biosci. 2022 Sep 13;9:878148. doi: 10.3389/fmolb.2022.878148 (PMC9513183; doi:10.3389/fmolb.2022.878148)
Supplement: Supplementary file 1 [file Presentation1.zip › Submitted_Supplementary_Material/Data_Tables.pdf]

Supplementary material: Dynamic modelling of DNA repair pathway at the molecular level: a new perspective

Paola Lecca, Faculty of Computer Science, Free University of Bozen-Bolzano, Italy
Adaoha E. C. Ihekwaba-Ndibe, Faculty of Health and Life Sciences, Coventry University, Coventry, UK

In this document we provide the node-to-node interactions in the networks considered in the study (Tables S1, S2, S3, S6) and a summary of the definitions of the centrality measures considered for their analysis (Tables S4 and S5).

Table S1. HR network as in Orlic-Milacic (2015) and its parametrization PART I).

NODE	INTERACTION	NODE	KINETIC RATE
BLM	in catalysis with	MRE11	k 1
BLM	in catalysis with	NBN	k 2
BLM	in catalysis with	POLD1	k 3
BLM	in catalysis with	POLD2	k 4
BLM	in catalysis with	POLD3	k 5
BLM	in catalysis with	POLD4	k 6
BLM	in catalysis with	RAD50	k 7
BLM	in catalysis with	RPA1	k 8
BLM	in catalysis with	RPA2	k 9
BLM	in catalysis with	RPA3	k 10
BLM	controls state change of	RPA2	k 11
EME1	controls state change of	BLM	k 12
EME1	controls state change of	BRCA2	k 13
EME1	controls state change of	MRE11	k 14
EME1	controls state change of	NBN	k 15
EME1	controls state change of	RAD50	k 16
EME1	controls state change of	RAD51	k 17
EME1	controls state change of	RAD51B	k 18
EME1	controls state change of	RAD51C	k 19
EME1	controls state change of	RAD51D	k 20
EME1	controls state change of	TOP3A	k 21
EME1	controls state change of	XRCC2	k 22
EME1	controls state change of	XRCC3	k 23
EME1	interacts with	MUS81	k 24
MRE11	in catalysis with	POLD1	k 25
MRE11	in catalysis with	POLD2	k 26
MRE11	in catalysis with	POLD3	k 27
MRE11	in catalysis with	POLD4	k 28
MRE11	in catalysis with	RPA1	k 29
MRE11	in catalysis with	RPA2	k 30
MRE11	in catalysis with	RPA3	k 31
MRE11	controls state change of	NBN	k 32
MRE11	controls state change of	RPA2	k 33
MRE11	interacts with	BLM	k 34
MUS81	controls state change of	BLM	k 35
MUS81	controls state change of	BRCA2	k 36
MUS81	controls state change of	MRE11	k 37
MUS81	controls state change of	NBN	k 38
MUS81	controls state change of	RAD50	k 39
MUS81	controls state change of	RAD51	k 40
MUS81	controls state change of	RAD51B	k 41
MUS81	controls state change of	RAD51C	k 42
MUS81	controls state change of	RAD51D	k 43
MUS81	controls state change of	TOP3A	k 44
MUS81	controls state change of	XRCC2	k 45
MUS81	controls state change of	XRCC3	k 46
NBN	in catalysis with	POLD1	k 47
NBN	in catalysis with	POLD2	k 48
NBN	in catalysis with	POLD3	k 49
NBN	in catalysis with	POLD4	k 50
NBN	in catalysis with	RPA1	k 51
NBN	in catalysis with	RPA2	k 52
NBN	in catalysis with	RPA3	k 53
NBN	controls state change of	RPA2	k 54
NBN	interacts with	BLM	k 55
NBN	interacts with	MRE11	k 56
POLD1	controls state change of	BLM	k 57
POLD1	controls state change of	BRCA2	k 58
POLD1	controls state change of	MRE11	k 59
POLD1	controls state change of	NBN	k 60
POLD1	controls state change of	RAD50	k 61
POLD1	controls state change of	RAD51	k 62
POLD1	controls state change of	RAD51B	k 63
POLD1	controls state change of	RAD51C	k 64
POLD1	controls state change of	RAD51D	k 65
POLD1	controls state change of	RPA1	k 66
POLD1	controls state change of	RPA2	k 67
POLD1	controls state change of	RPA3	k 68
POLD1	controls state change of	TOP3A	k 69
POLD1	controls state change of	XRCC2	k 70
POLD1	controls state change of	XRCC3	k 71
POLD1	interacts with	POLD3	k 72
POLD1	interacts with	POLD4	k 73
POLD2	controls state change of	BLM	k 74
POLD2	controls state change of	BRCA2	k 75
POLD2	controls state change of	MRE11	k 76
POLD2	controls state change of	NBN	k 77
POLD2	controls state change of	RAD50	k 78
POLD2	controls state change of	RAD51	k 79
POLD2	controls state change of	RAD51B	k 80
POLD2	controls state change of	RAD51C	k 81
POLD2	controls state change of	RAD51D	k 82
POLD2	controls state change of	RPA1	k 83
POLD2	controls state change of	RPA2	k 84
POLD2	controls state change of	RPA3	k 85
POLD2	controls state change of	TOP3A	k 86
POLD2	controls state change of	XRCC2	k 87
POLD2	controls state change of	XRCC3	k 88
POLD2	interacts with	POLD1	k 89
POLD2	interacts with	POLD3	k 90

Table S2. HR network as in Orlic-Milacic (2015) and its parametrization PART II).

NODE	INTERACTION	NODE	KINETIC RATE
POLD2	interacts with	POLD4	k 91
POLD3	controls state change of	BLM	k 92
POLD3	controls state change of	BRCA2	k 93
POLD3	controls state change of	MRE11	k 94
POLD3	controls state change of	NBN	k 95
POLD3	controls state change of	RAD50	k 96
POLD3	controls state change of	RAD51	k 97
POLD3	controls state change of	RAD51B	k 98
POLD3	controls state change of	RAD51C	k 99
POLD3	controls state change of	RAD51D	k 100
POLD3	controls state change of	RPA1	k 101
POLD3	controls state change of	RPA2	k 102
POLD3	controls state change of	RPA3	k 103
POLD3	controls state change of	TOP3A	k 104
POLD3	controls state change of	XRCC2	k 105
POLD3	controls state change of	XRCC3	k 106
POLD4	controls state change of	BLM	k 107
POLD4	controls state change of	BRCA2	k 108
POLD4	controls state change of	MRE11	k 109
POLD4	controls state change of	NBN	k 110
POLD4	controls state change of	RAD50	k 111
POLD4	controls state change of	RAD51	k 112
POLD4	controls state change of	RAD51B	k 113
POLD4	controls state change of	RAD51C	k 114
POLD4	controls state change of	RAD51D	k 115
POLD4	controls state change of	RPA1	k 116
POLD4	controls state change of	RPA2	k 117
POLD4	controls state change of	RPA3	k 118
POLD4	controls state change of	TOP3A	k 119
POLD4	controls state change of	XRCC2	k 120
POLD4	controls state change of	XRCC3	k 121
POLD4	interacts with	POLD3	k 122
RAD50	in catalysis with	POLD1	k 123
RAD50	in catalysis with	POLD2	k 124
RAD50	in catalysis with	POLD3	k 125
RAD50	in catalysis with	POLD4	k 126
RAD50	in catalysis with	RPA1	k 127
RAD50	in catalysis with	RPA2	k 128
RAD50	in catalysis with	RPA3	k 129
RAD50	controls state change of	NBN	k 130
RAD50	controls state change of	RPA2	k 131
RAD50	interacts with	BLM	k 132
RAD50	interacts with	MRE11	k 133
RAD50	interacts with	NBN	k 134
RAD50	interacts with	RPA1	k 135
RAD50	interacts with	RPA2	k 136
RAD51	controls expression of	RAD50	k 137
RAD51	interacts with	BLM	k 138
RAD51	interacts with	BRCA2	k 139
RAD51	interacts with	RAD51B	k 140
RAD51	interacts with	RAD51D	k 141
RAD51	interacts with	RAD52	k 142
RAD51	interacts with	RPA1	k 143
RAD51	interacts with	RPA2	k 144
RAD51	interacts with	SEM1	k 145
RAD51	interacts with	XRCC2	k 146
RAD51	interacts with	XRCC3	k 147
RAD51B	interacts with	RPA1	k 148
RAD51B	interacts with	XRCC2	k 149
RAD51C	controls state change of	BRCA2	k 150
RAD51C	controls state change of	RAD51	k 151
RAD51C	controls state change of	RPA1	k 152
RAD51C	controls state change of	RPA2	k 153
RAD51C	controls state change of	RPA3	k 154
RAD51C	interacts with	BRCA2	k 155
RAD51C	interacts with	RAD51	k 156
RAD51C	interacts with	RAD51B	k 157
RAD51C	interacts with	RAD51D	k 158
RAD51C	interacts with	RPA1	k 159
RAD51C	interacts with	XRCC2	k 160
RAD51C	interacts with	XRCC3	k 161
RAD51D	interacts with	BLM	k 162
RAD51D	interacts with	RAD51B	k 163
RAD51D	interacts with	RPA1	k 164
RAD51D	interacts with	XRCC2	k 165
RAD51D	interacts with	XRCC3	k 166
RAD52	interacts with	RPA1	k 167
RAD52	interacts with	RPA2	k 168
RPA1	controls state change of	BLM	k 169
RPA1	controls state change of	BRCA2	k 170
RPA1	controls state change of	MRE11	k 171
RPA1	controls state change of	NBN	k 172
RPA1	controls state change of	RAD50	k 173
RPA1	controls state change of	RAD51	k 174
RPA1	controls state change of	RAD51B	k 175
RPA1	controls state change of	RAD51C	k 176
RPA1	controls state change of	RAD51D	k 177
RPA1	controls state change of	RPA2	k 178
RPA1	controls state change of	TOP3A	k 179
RPA1	controls state change of	XRCC2	k 180

Table S3. HR network as in Orlic-Milacic (2015) and its parametrization PART III).

NODE	INTERACTION	NODE	KINETIC RATE
RPA1	controls state change of	XRCC3	k 181
RPA1	interacts with	BLM	k 182
RPA1	interacts with	BRCA2	k 183
RPA1	interacts with	MRE11	k 184
RPA1	interacts with	NBN	k 185
RPA1	interacts with	POLD1	k 186
RPA1	interacts with	TOP3A	k 187
RPA1	interacts with	TOP3B	k 188
RPA1	interacts with	XRCC2	k 189
RPA2	controls state change of	BLM	k 190
RPA2	controls state change of	BRCA2	k 191
RPA2	controls state change of	MRE11	k 192
RPA2	controls state change of	NBN	k 193
RPA2	controls state change of	RAD50	k 194
RPA2	controls state change of	RAD51	k 195
RPA2	controls state change of	RAD51B	k 196
RPA2	controls state change of	RAD51C	k 197
RPA2	controls state change of	RAD51D	k 198
RPA2	controls state change of	TOP3A	k 199
RPA2	controls state change of	XRCC2	k 200
RPA2	controls state change of	XRCC3	k 201
RPA2	interacts with	BLM	k 202
RPA2	interacts with	BRCA2	k 203
RPA2	interacts with	MRE11	k 204
RPA2	interacts with	NBN	k 205
RPA2	interacts with	POLD1	k 206
RPA2	interacts with	RPA1	k 207
RPA2	interacts with	TOP3A	k 208
RPA3	controls state change of	BLM	k 209
RPA3	controls state change of	BRCA2	k 210
RPA3	controls state change of	MRE11	k 211
RPA3	controls state change of	NBN	k 212
RPA3	controls state change of	RAD50	k 213
RPA3	controls state change of	RAD51	k 214
RPA3	controls state change of	RAD51B	k 215
RPA3	controls state change of	RAD51C	k 216
RPA3	controls state change of	RAD51D	k 217
RPA3	controls state change of	RPA2	k 218
RPA3	controls state change of	TOP3A	k 219
RPA3	controls state change of	XRCC2	k 220
RPA3	controls state change of	XRCC3	k 221
RPA3	interacts with	BLM	k 222
RPA3	interacts with	MRE11	k 223
RPA3	interacts with	NBN	k 224
RPA3	interacts with	POLD1	k 225
RPA3	interacts with	RAD50	k 226
RPA3	interacts with	RAD51	k 227
RPA3	interacts with	RAD52	k 228
RPA3	interacts with	RPA1	k 229
RPA3	interacts with	RPA2	k 230
RPA3	interacts with	TOP3A	k 231
RPA4	interacts with	RPA1	k 232
RPA4	interacts with	RPA2	k 233
RPA4	interacts with	RPA3	k 234
SEM1	interacts with	BRCA2	k 235
SEM1	interacts with	RPA1	k 236
TOP3A	in catalysis with	MRE11	k 237
TOP3A	in catalysis with	NBN	k 238
TOP3A	in catalysis with	POLD1	k 239
TOP3A	in catalysis with	POLD2	k 240
TOP3A	in catalysis with	POLD3	k 241
TOP3A	in catalysis with	POLD4	k 242
TOP3A	in catalysis with	RAD50	k 243
TOP3A	in catalysis with	RPA1	k 244
TOP3A	in catalysis with	RPA2	k 245
TOP3A	in catalysis with	RPA3	k 246
TOP3A	controls state change of	RPA2	k 247
TOP3A	interacts with	BLM	k 248
XRCC2	interacts with	BLM	k 249
XRCC3	interacts with	BRCA2	k 250

Table S4. Definitions of centrality measures considered in this study.

Degree centrality
The Out-degree of a node is the number of edges starting from it. The In-degree of a node is the number of edges ending to it. The total degree (simply said "degree") of a node is the sum of out- and in-degree of it.

Betweenness centrality
Betweenness centrality quantifies the relative importance of a node in the communication between other pairs of nodes. Nodes with high betweenness centrality highly facilitate or highly inhibit the communication between other nodes in the network.
Assuming that the information from one node to another node travels only through the shortest paths connecting those nodes, we can express mathematically the betweenness centrality of a node i as

$$BC_i = \sum_i \sum_j \frac{\rho(i,k,j)}{\rho(i,j)}$$

Where $\rho(i,j)$ is the number of shortest paths from node i to node j, and $\rho(i,k,j)$ is the number of these shortest paths that pass through node k.

Clustering coefficient
The clustering coefficient C_i of a vertex i is the frequency of pairs of neighbours of i that are connected by an edge, that is, it is the ratio of the number m_i of pairs of neighbours of i that are connected and the number of possible pairs of neighbours of i, which is $k_i(k_i-1)/2$, where k_i is the degree of i:

$$C_i = \frac{2m_i}{k_i(k_i-1)}$$

C_i can be also interpreted as the probability that a pair of neighbours of i are connected. It quantifies how close its neighbours are to being a clique (complete graph).

Eigenvector centrality
Eigenvector centrality is an extension of degree centrality. In-degree centrality awards one centrality point for every link a node receives. However since not all vertices are equivalent ()some are more important than others), and, reasonably, endorsements from important nodes count more. Eigenvector centrality assumes the thesis by which "a node is important if it is linked to by other important nodes".
Let $\mathbf{A} = (a_{i,})$ be the adjacency matrix of a graph (i.e. $a_{ij} = 1$ if node i is linked to vertex j, and $a_{ij} = 0$ otherwise). The eigenvector centrality EV_i of node i is recursively defined:

$$EV_i = \frac{1}{\lambda} \sum_{k \in N(i)} a_{ki} EV_k$$

where $N(i)$ is the set of neighbours of i, and initial vertex centrality $EV_k = 1, \forall k$ including i. $\lambda \neq 0$ is a constant such that $\lambda \mathbf{x} = A\mathbf{x}$.

Subgraph centrality
The subgraph centrality of the node i is the number of closed walks of different lengths in the network starting and ending at node i. Mathematically, the number of closed walks of length L starting and ending on node i is given by the i-th diagonal entry of the L-th power of the adjacency matrix A:

$$SC_i = (A^L)_{ii}.$$

Subgraph centrality characterizes the participation of each node in all subgraphs in a network. Smaller subgraphs are given more weight than larger ones, so that subgraph centrality measure is appropriate for characterizing network motifs.

Information centrality
The weighted function of the set of all paths connecting node i to node j is any weighted linear combination of the paths such that the sum of the weights is unity. Assuming that each link in a path is independent, and the variance of a single link is unity, it can be concluded that the variance of a path is simply its length. The information measure between two nodes i and j is the inverse of the variance of the weighted function. Mathematically it is defined as the harmonic mean of all the information measures between i and all other nodes in the network"

$$IC_i = \left[\frac{1}{n} \sum_j \frac{1}{\mathbf{I}_{ij}}\right]$$

where n is the number of nodes, $\mathbf{I}_{ij} = (c_{ii} + c_{jj} - c_{ij})^{-1}$, and $\mathbf{C} = (cij) = \mathbf{D} - \mathbf{A} + \mathbf{J}$ with $\mathbf{D}$ the degree matrix, $\mathbf{A}$ the adjacency matrix, and $\mathbf{J}$ a matrix with all elements equal to 1.

Hub centrality
The hub scores (also know as Kleinberg centralities) of the nodes are defined as the principal eigenvector of $\mathbf{A}\mathbf{A}^T$, where $\mathbf{A}$ is the adjacency matrix of the graph. Kleinberg centrality is a generalization of eigenvector centrality that prevents the problem of ordinary eigenvector centrality on directed networks, that nodes outside strongly connected components or their out-components get null centrality.

Table S5. Vibrational centrality, a new centrality measure introduced by Estrada et al. (Estrada and Hatano, 2010; Estrada, 2011).

The definition of vibrational centrality arose in the context of a vibrational approach to complex network in order to study reaction of a node to perturbation. Vibrational centrality quantifies the displacement of a node due to small oscillation of vibrations in the network. The displacement of a node i is defined as $$(\Delta x_i)^2 = \frac{1}{\beta\theta}(\mathbf{L}^+)_{ii}$$ where $\mathbf{L}^+$ is the generalized Moore-Penrose pseudo-inverse of the graph Laplacian $\mathbf{L} = \mathbf{D} - \mathbf{A}$ with $\mathbf{D}$ the degree matrix, and $\mathbf{A}$ the adjacency matrix. A physical model of the network as a set of nodes connected by springs i given and the network is supposed to be affected by an external stress. In the Estrada et al. (Estrada and Hatano, 2010) physical model this is simulated by immersing the network into a thermal bath of inverse temperature $1/\beta$. This scaling factor accounts for the external effects to which a real network can be submitted, such as different levels of stresses due to pharmacological treatment, knock-down or know-out experiments in gene networks. The concept of vibrational centrality extends also to socio-economics , IT networks, ecological networks. We quote Estrada's exact words in (Estrada and Hatano, 2010): " Returning to a real-world situation, the displacement of a node given by represents how much the corresponding node is affected by the external stress to which the network is submitted to. A node which displays a large value of the displacement is one which is very much affected by the external conditions, such as economical crisis, social agitation, environmental pressure or physiological conditions. In other words, it is a node with high vulnerability to the change in the external conditions."

Table S6. FANCM pathways as in (Pathways Commons, 2022).

NODE	INTERACTION	NODE
BLM	in catalysis with	FANCM
BLM	interacts with	FANCM
BRCA1	in catalysis with	FANCM
BRCA1	interacts with	FANCM
CENPS	controls state change of	FANCM
CENPS	interacts with	FANCM
CENPX	controls state change of	FANCM
CENPX	interacts with	FANCM
EME1	controls state change of	FANCM
EME1	interacts with	FANCM
EME2	controls state change of	FANCM
EME2	interacts with	FANCM
FAAP100	controls state change of	FANCM
FAAP100	interacts with	FANCM
FAAP20	controls state change of	FANCM
FAAP20	interacts with	FANCM
FAAP24	controls state change of	FANCM
FAAP24	interacts with	FANCM
FANCA	controls state change of	FANCM
FANCA	interacts with	FANCM
FANCB	controls state change of	FANCM
FANCB	interacts with	FANCM
FANCC	controls state change of	FANCM
FANCC	interacts with	FANCM
FANCD2	controls state change of	FANCM
FANCE	controls state change of	FANCM
FANCE	interacts with	FANCM
FANCF	controls state change of	FANCM
FANCF	interacts with	FANCM
FANCG	controls state change of	FANCM
FANCG	interacts with	FANCM
FANCI	controls state change of	FANCM
FANCI	interacts with	FANCM
FANCL	controls state change of	FANCM
FANCL	interacts with	FANCM
FANCM	controls state change of	FAAP100
FANCM	controls state change of	FANCA
FANCM	controls state change of	FANCB
FANCM	controls state change of	FANCC
FANCM	controls state change of	FANCD2
FANCM	controls state change of	FANCE
FANCM	controls state change of	FANCF
FANCM	controls state change of	FANCG
FANCM	controls state change of	FANCI
FANCM	controls state change of	FANCL
FANCM	controls state change of	HES1
FANCM	controls state change of	UBE2T
FANCM	controls transport of	FAAP100
FANCM	controls transport of	FANCA
FANCM	controls transport of	FANCB
FANCM	controls transport of	FANCC
FANCM	controls transport of	FANCE
FANCM	controls transport of	FANCF
FANCM	controls transport of	FANCG
FANCM	controls transport of	FANCL
FANCM	controls transport of	HES1
FANCM	controls transport of	UBE2T
RMI1	in catalysis with	FANCM
RMI1	interacts with	FANCM
RMI2	in catalysis with	FANCM
RMI2	interacts with	FANCM
RPA1	controls state change of	FANCM
RPA1	interacts with	FANCM
TOP3A	in catalysis with	FANCM
TOP3A	interacts with	FANCM
UBE2T	controls state change of	FANCM

REFERENCES

[Dataset] Orlic-Milacic M. HDR through homologous recombination (hrr). https://apps.pathwaycommons.org/search?q=homologous%20recombinations&type=Pathway (2015). Accessed: 2021-10-01.

Estrada E, Hatano N. A vibrational approach to node centrality and vulnerability in complex networks. *Physica A: Statistical Mechanics and its Applications* **389** (2010) 3648–3660. doi:10.1016/j.physa.2010.03.030.

Estrada E. *The Structure of Complex Networks* (Oxford University Press) (2011). doi:10.1093/acprof:oso/9780199591756.001.0001.

[Dataset] Pathways Commons. Pathways Commons (2022). Https://apps.pathwaycommons.org/search?type=Pathway&q=FANCM.
