# Supplementary material for: Dynamic Modelling of DNA Repair Pathway at the Molecular Level: A New Perspective
Source: Front Mol Biosci. 2022 Sep 13;9:878148. doi: 10.3389/fmolb.2022.878148 (PMC9513183; doi:10.3389/fmolb.2022.878148)
Supplement: Supplementary file 1 [file Presentation1.zip › Submitted_Supplementary_Material/Simulations_of_Dynamics_HR_FANCM_pathwys.pdf]

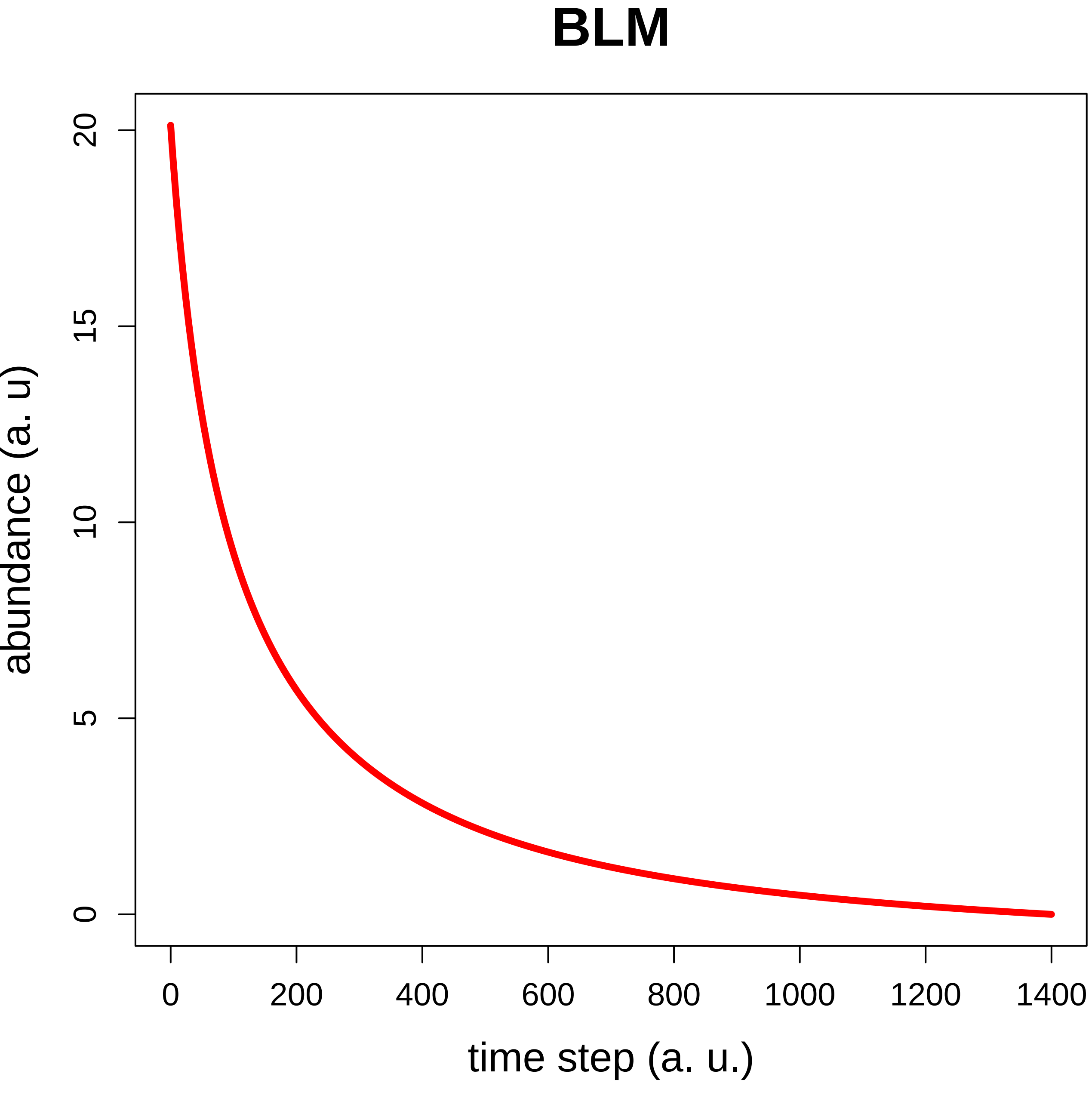
BLM
abundance (a. u)
0
5
10
15
20
0
200
400
600
800
1000
1200
1400
time step (a. u.)

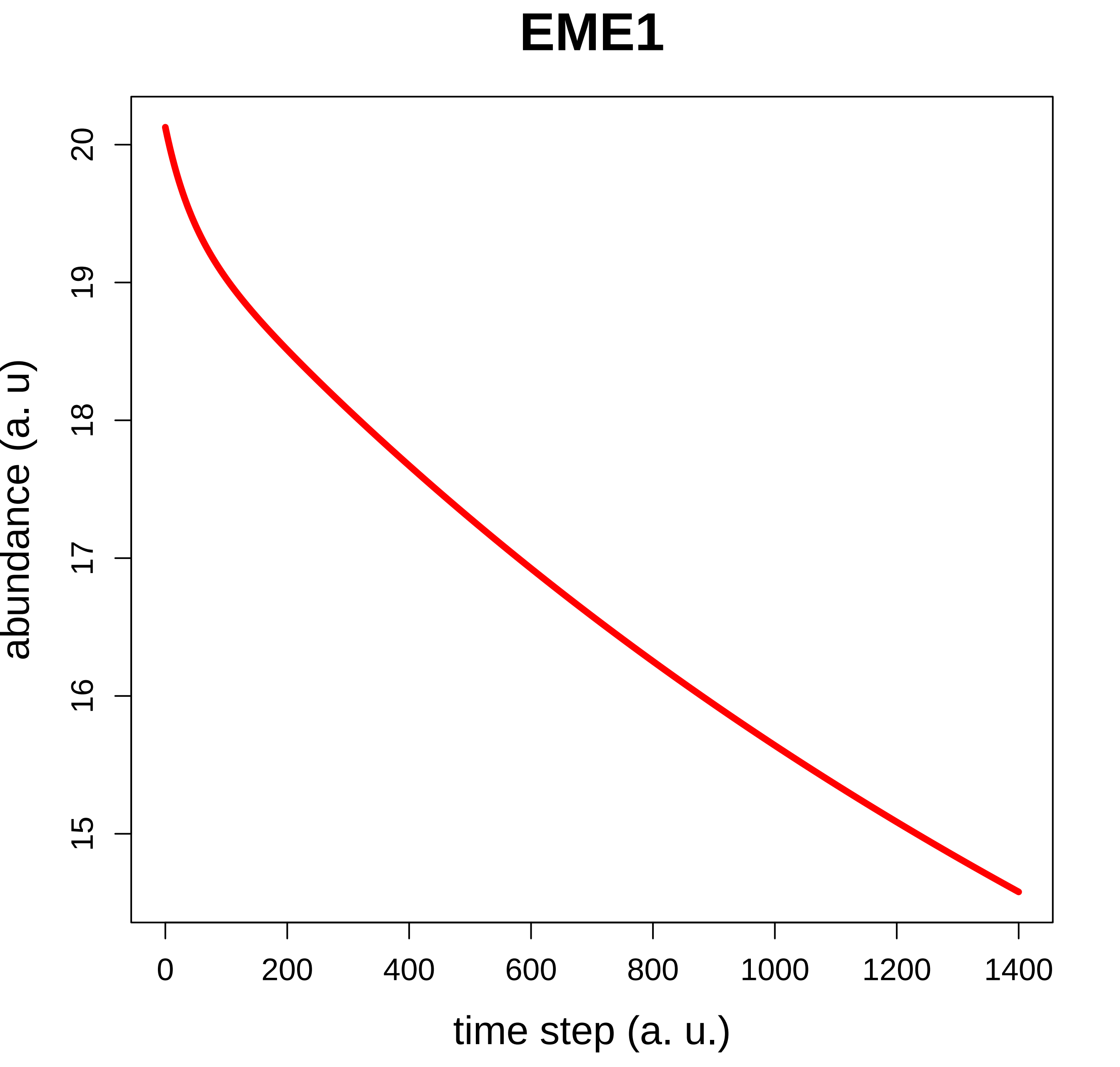

EME1
abundance (a. u)
time step (a. u.)
15
16
17
18
19
20
0
200
400
600
800
1000
1200
1400

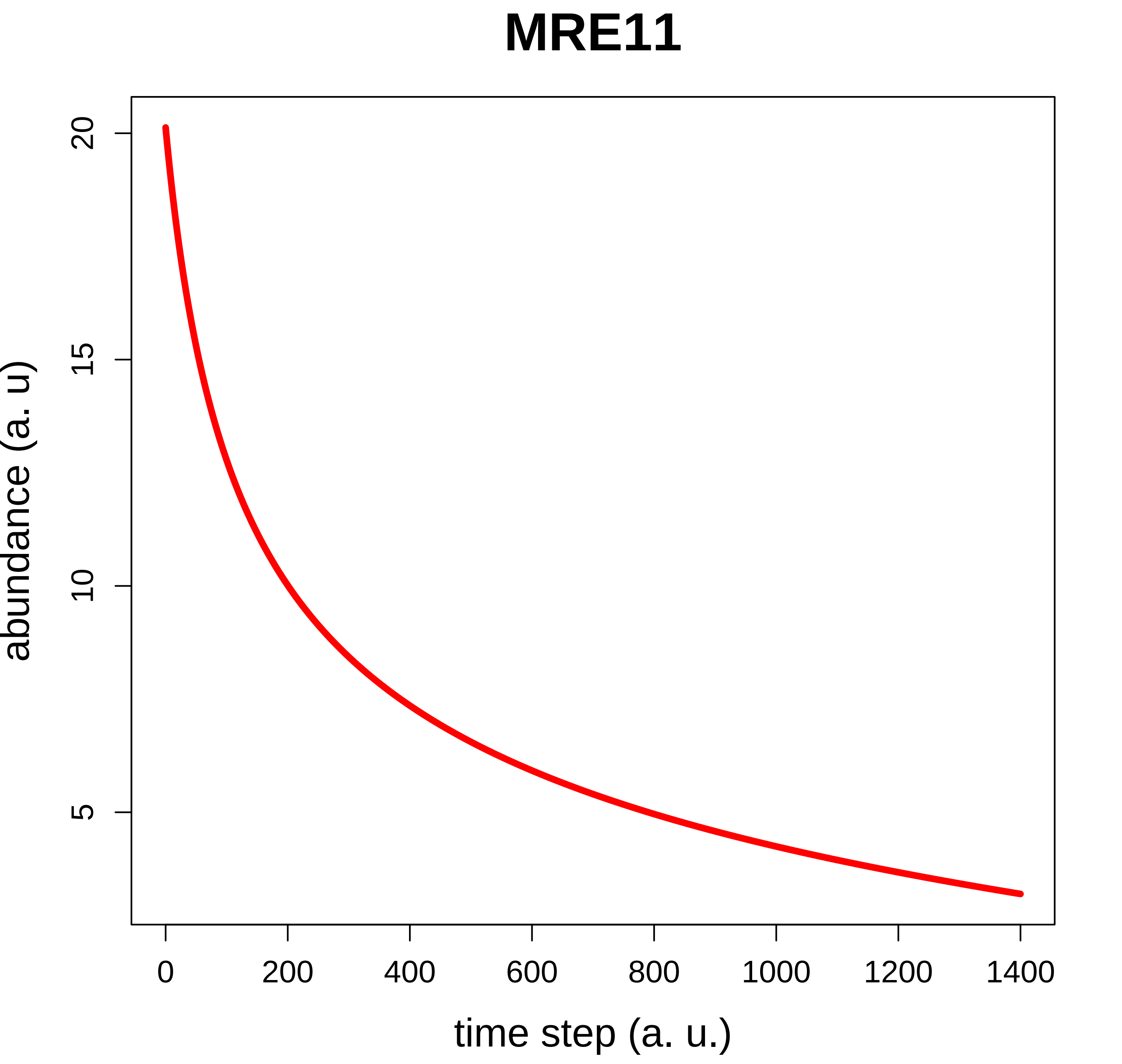

MRE11
abundance (a. u)
20
15
10
5
0
200
400
600
800
1000
1200
1400
time step (a. u.)

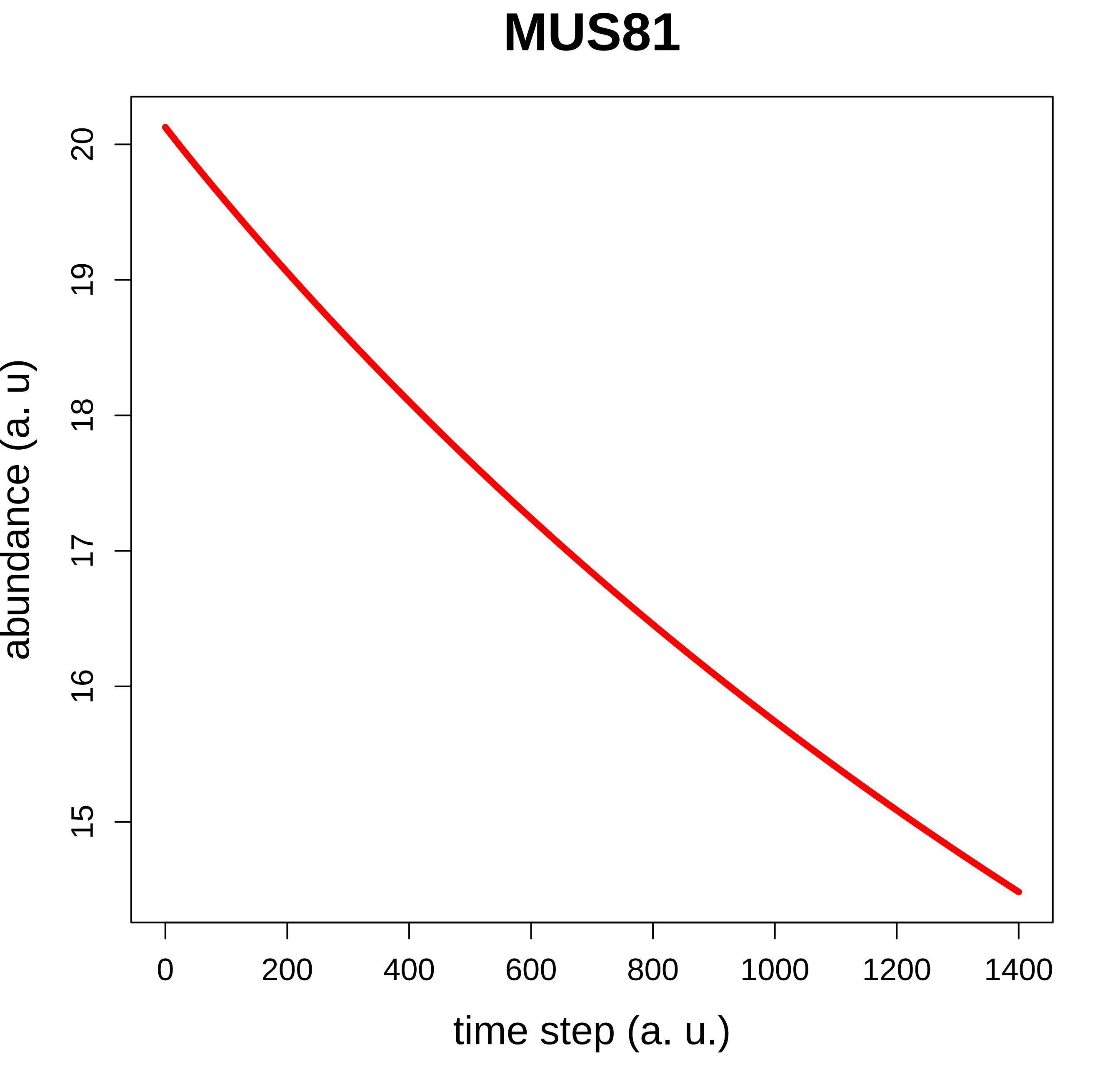

MUS81
abundance (a. u)
time step (a. u.)
15
16
17
18
19
20
0
200
400
600
800
1000
1200
1400

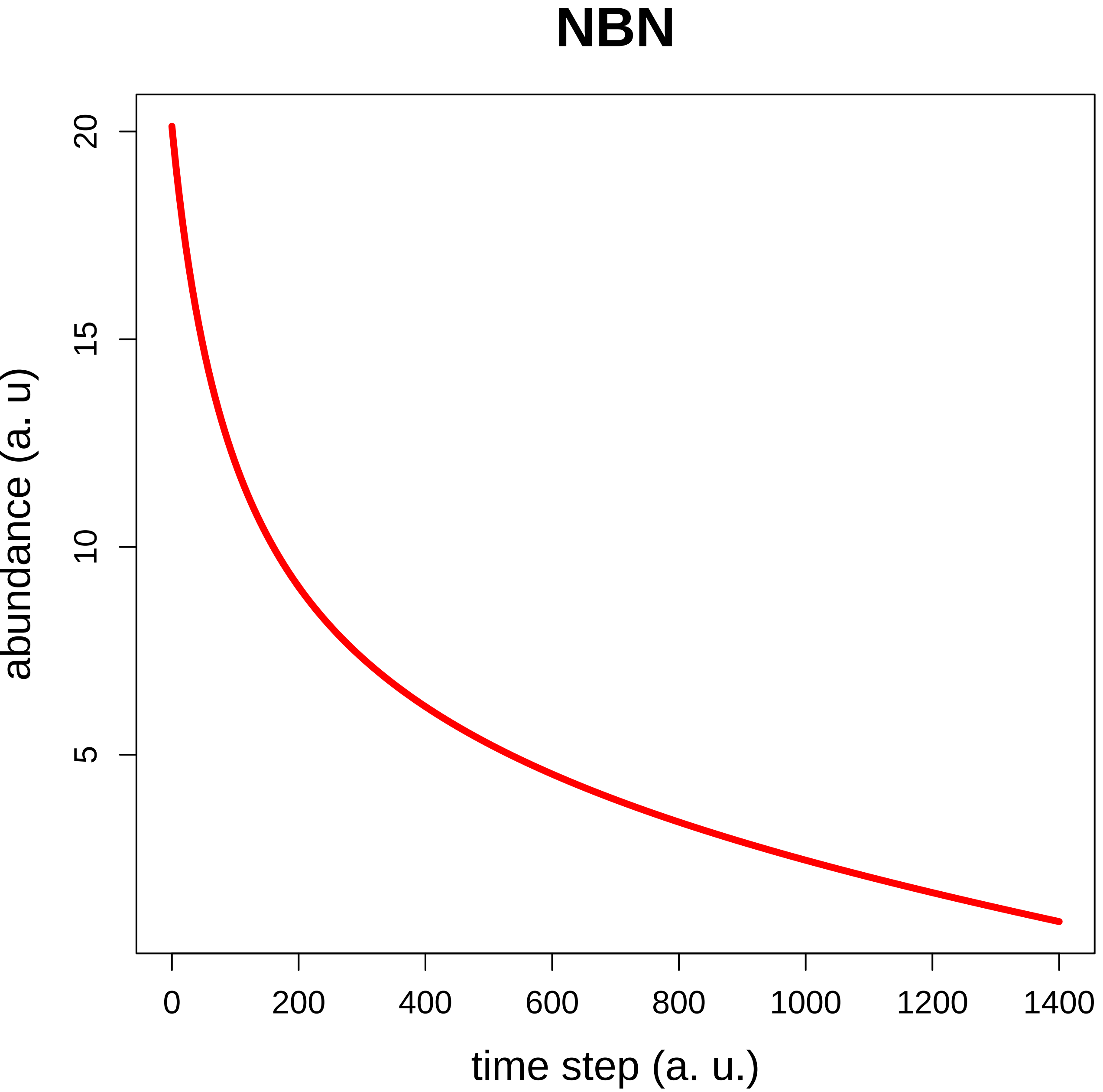
NBN
abundance (a. u)
20
15
10
5
0
200
400
600
800
1000
1200
1400
time step (a. u.)

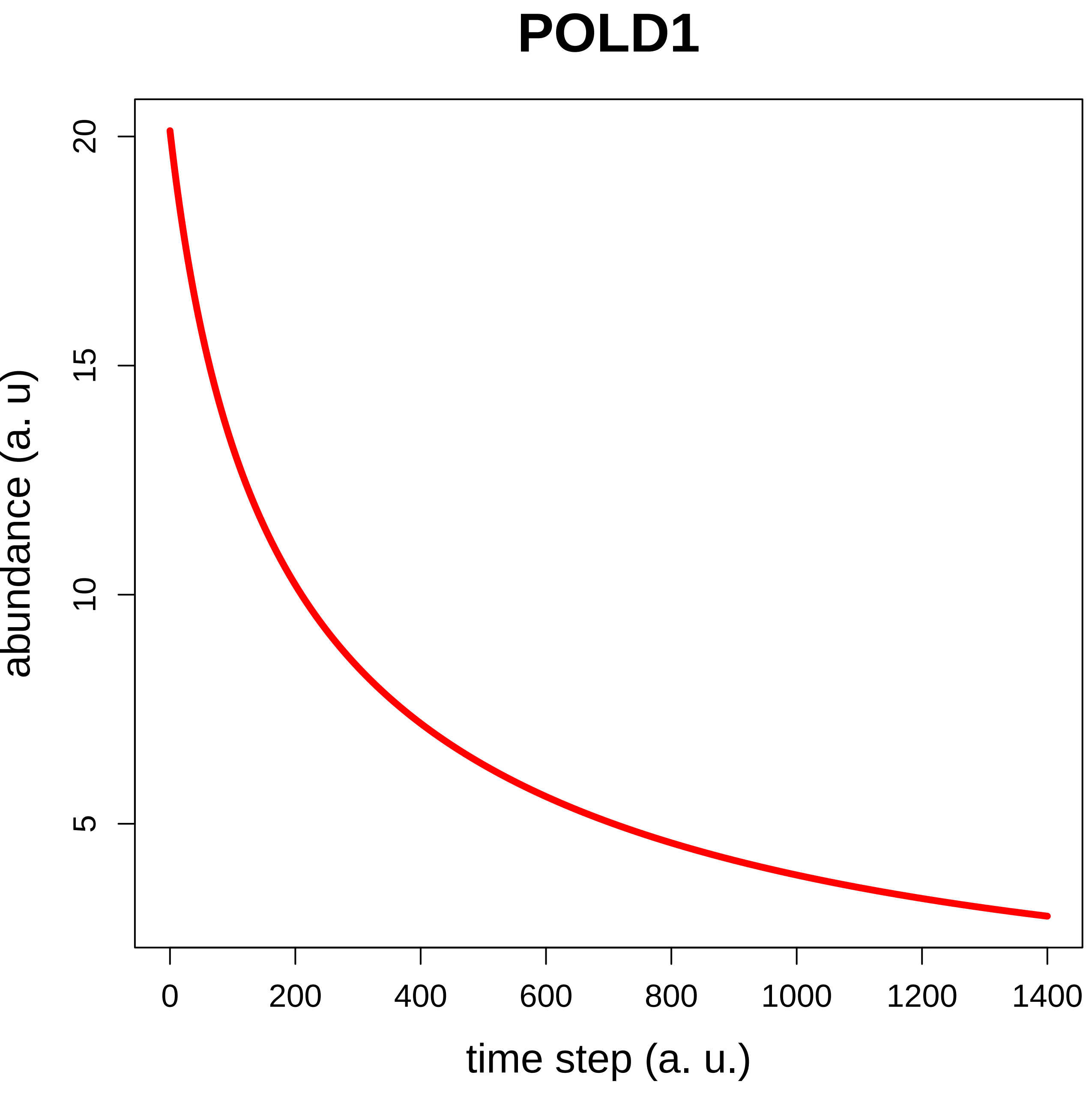

POLD1
abundance (a. u)
20
15
10
5
0
200
400
600
800
1000
1200
1400
time step (a. u.)

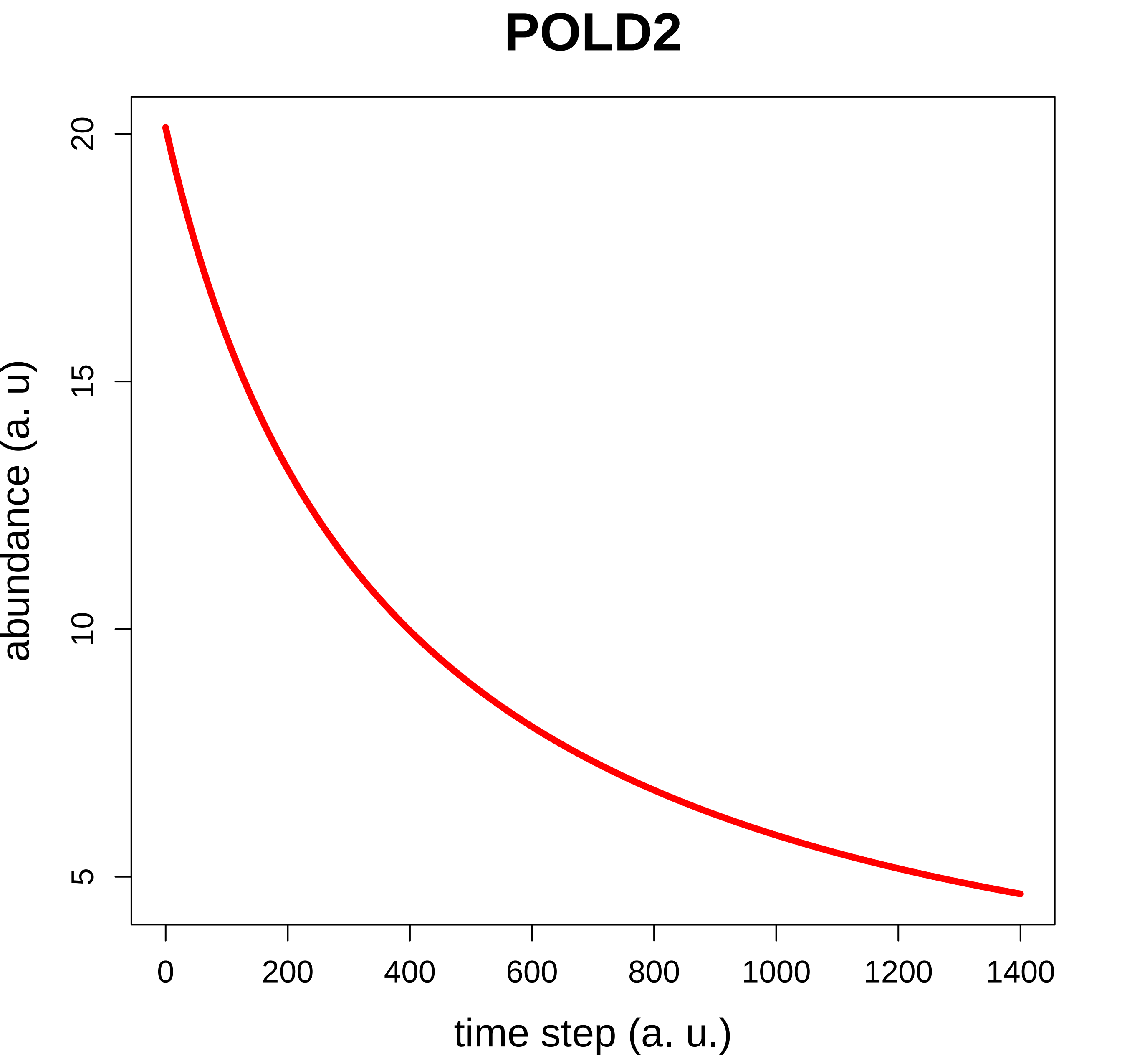

POLD2
abundance (a. u)
20
15
10
5
0
200
400
600
800
1000
1200
1400
time step (a. u.)

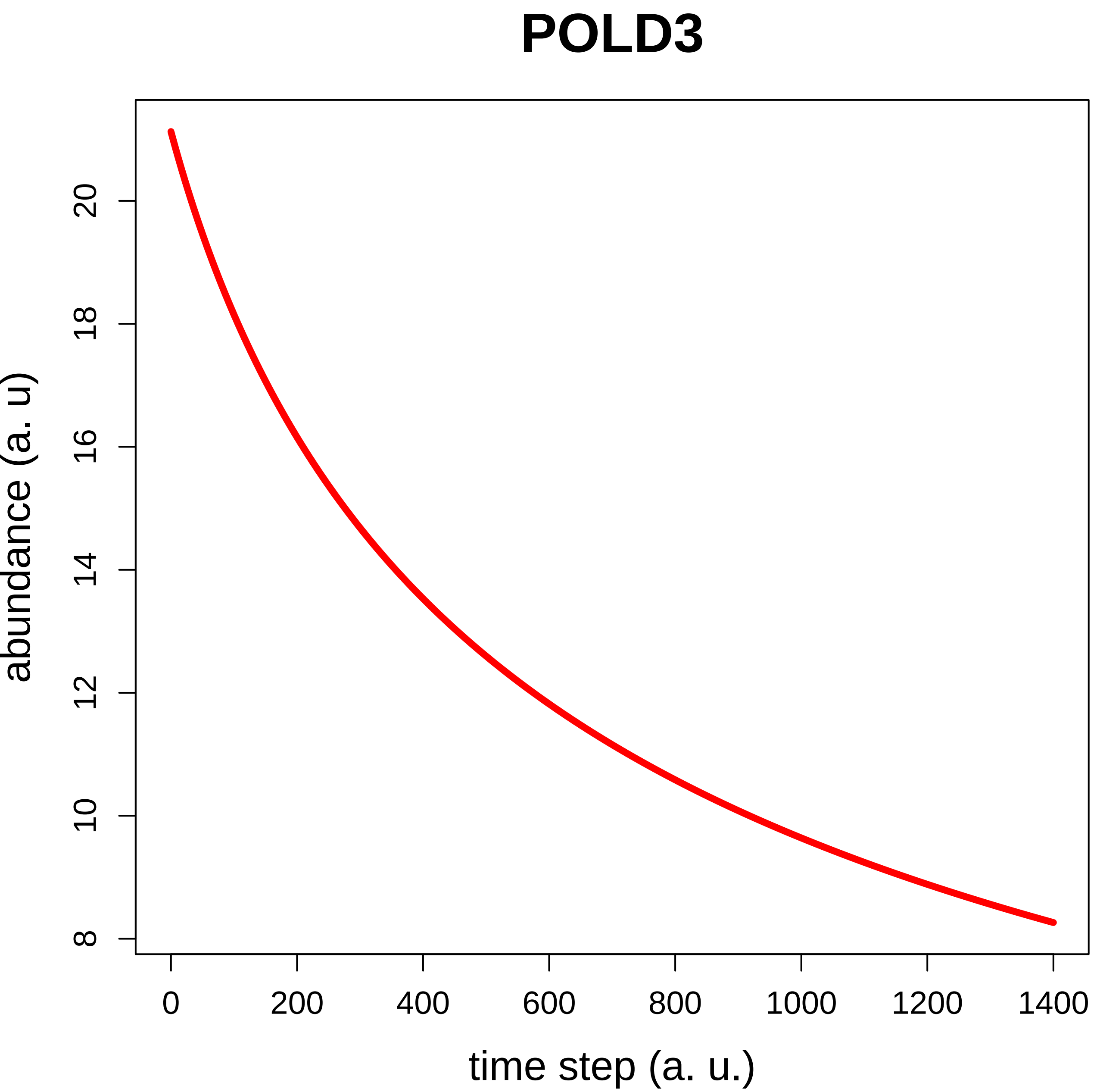

POLD3
abundance (a. u)
20
18
16
14
12
10
8
0
200
400
600
800
1000
1200
1400
time step (a. u.)

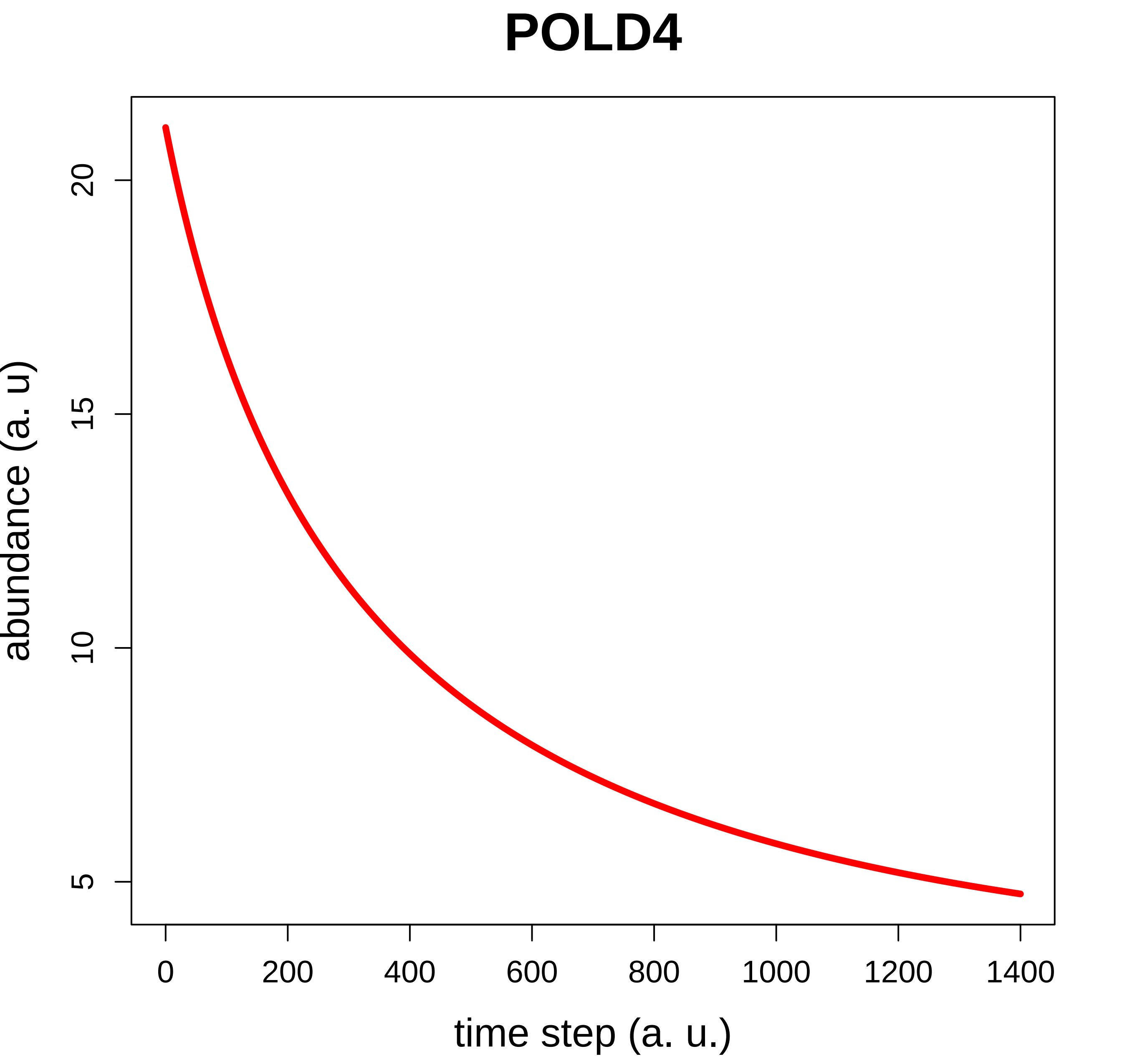

POLD4
20
15
10
5
abundance (a. u)
0
200
400
600
800
1000
1200
1400
time step (a. u.)

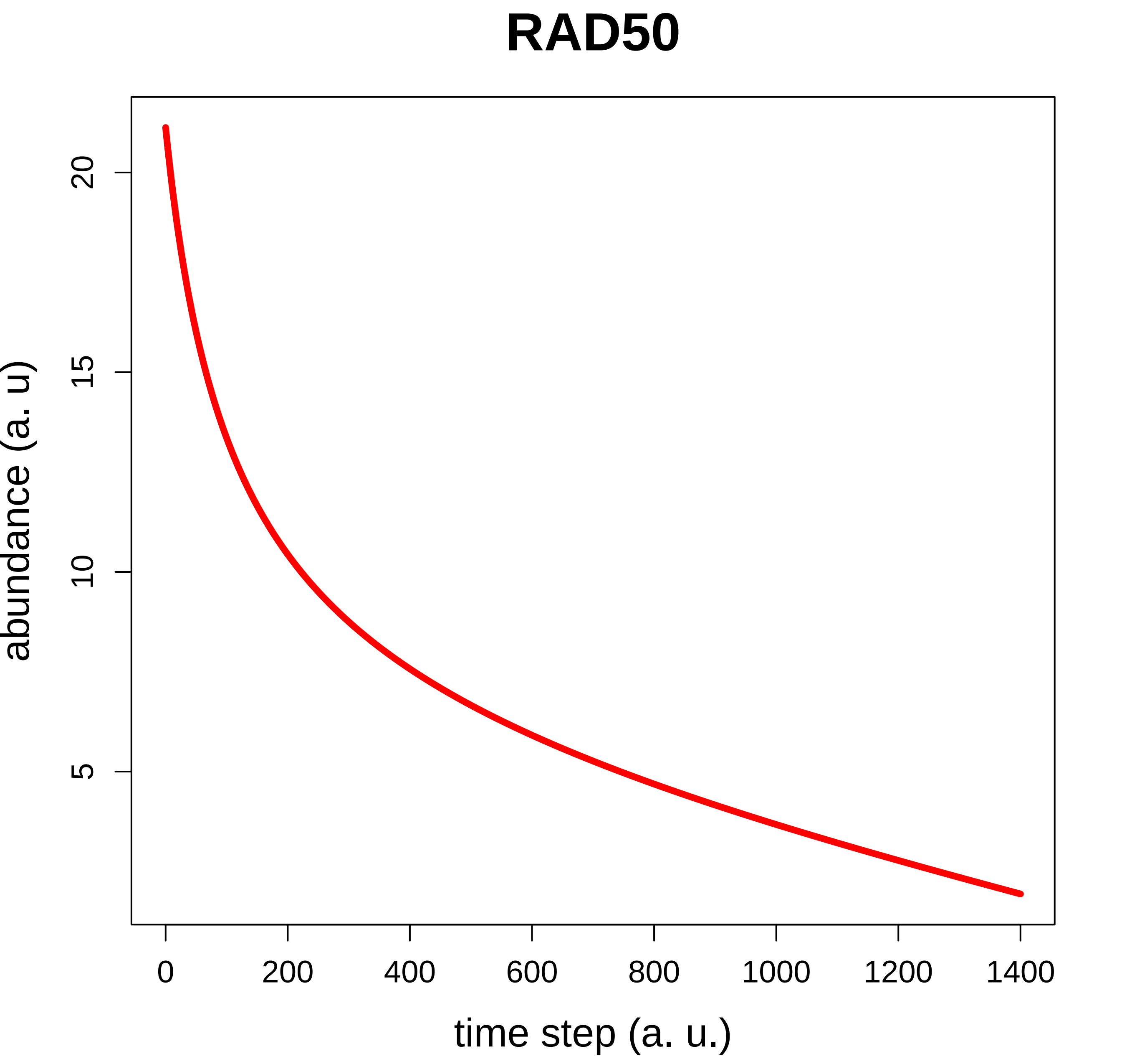
RAD50
abundance (a. u)
5
10
15
20
0
200
400
600
800
1000
1200
1400
time step (a. u.)

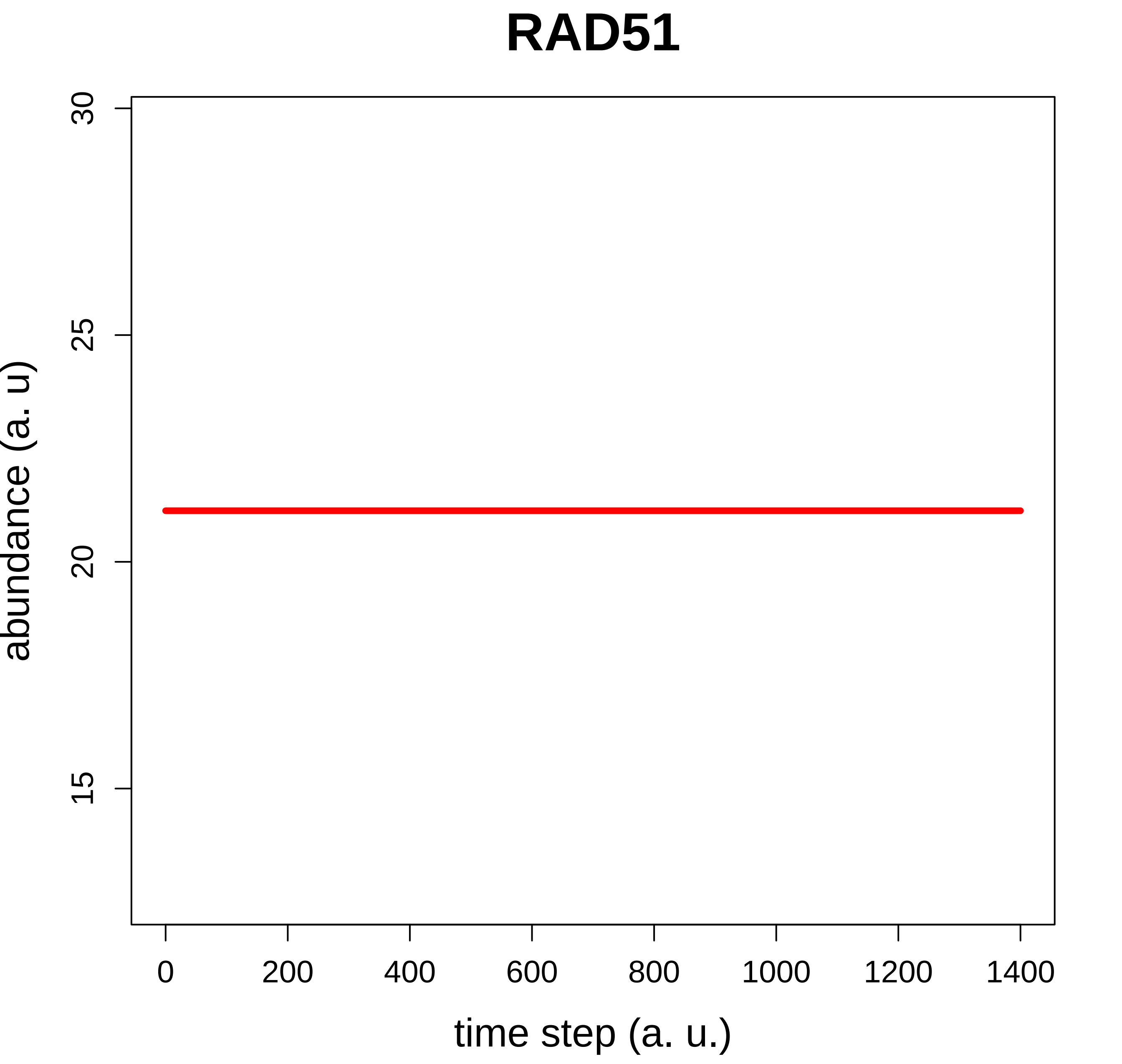
RAD51
abundance (a. u)
time step (a. u.)
30
25
20
15
0
200
400
600
800
1000
1200
1400

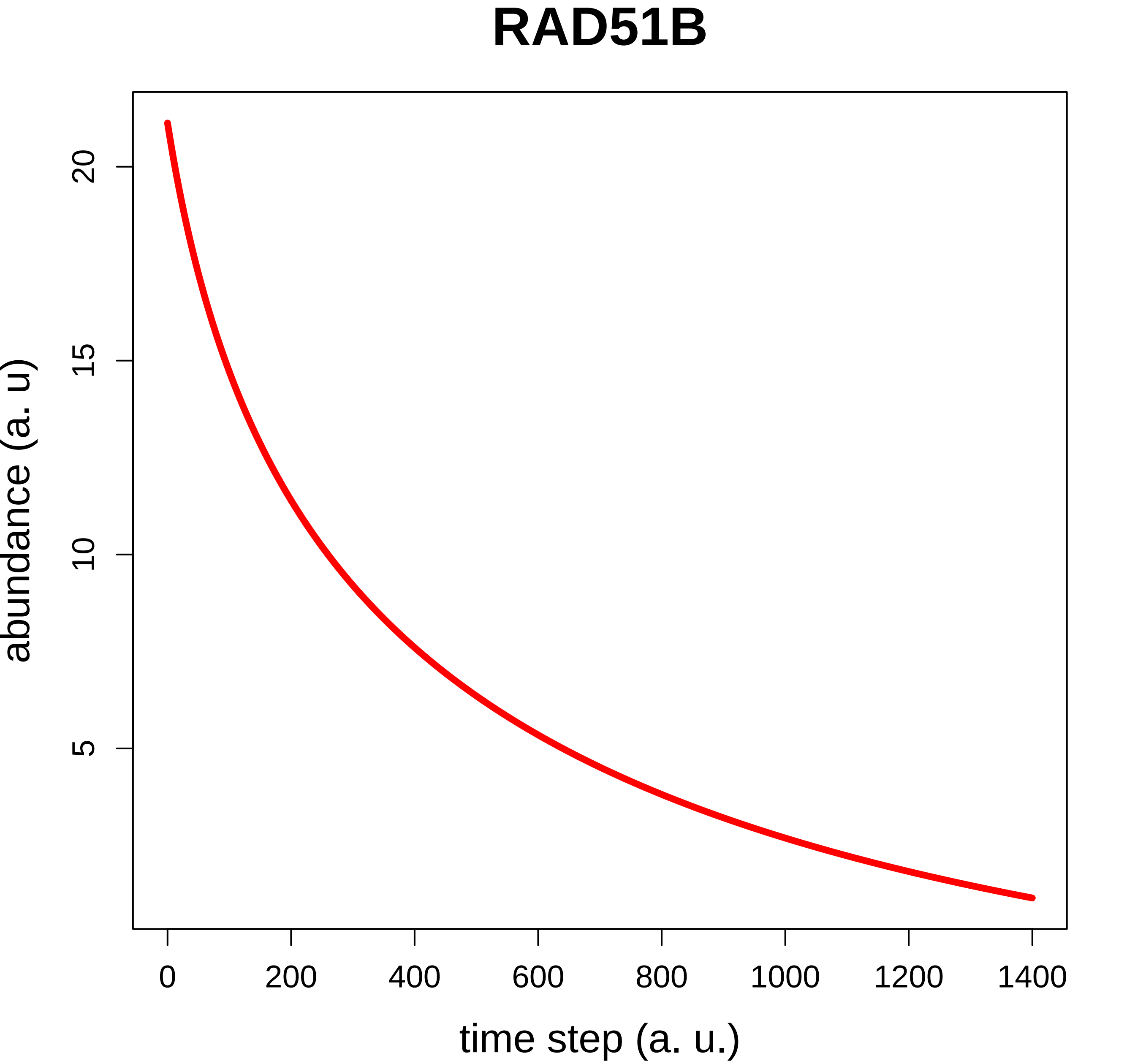

RAD51B
abundance (a. u)
time step (a. u.)
5
10
15
20
0
200
400
600
800
1000
1200
1400

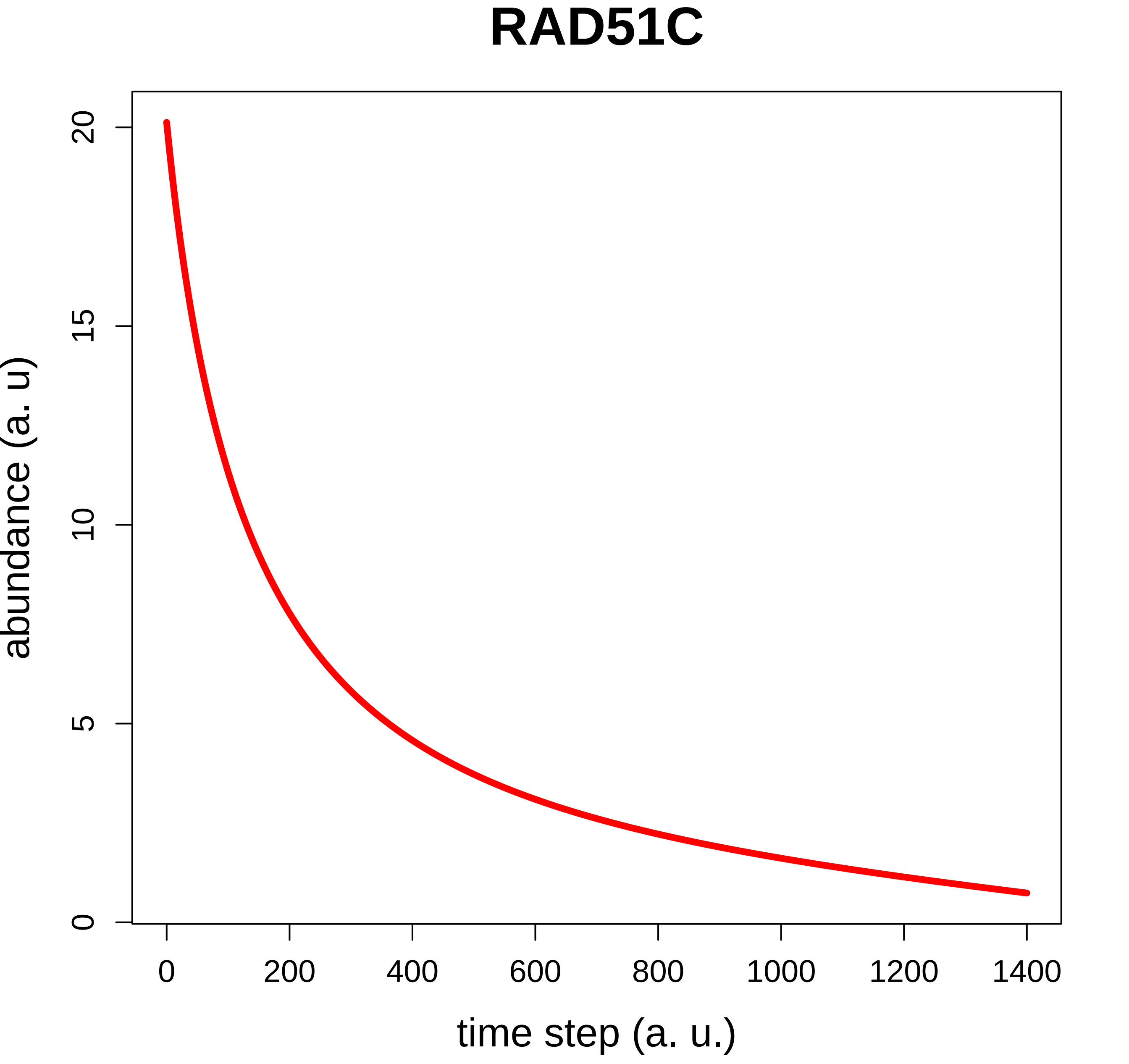
RAD51C
abundance (a. u)
20
15
10
5
0
0
200
400
600
800
1000
1200
1400
time step (a. u.)

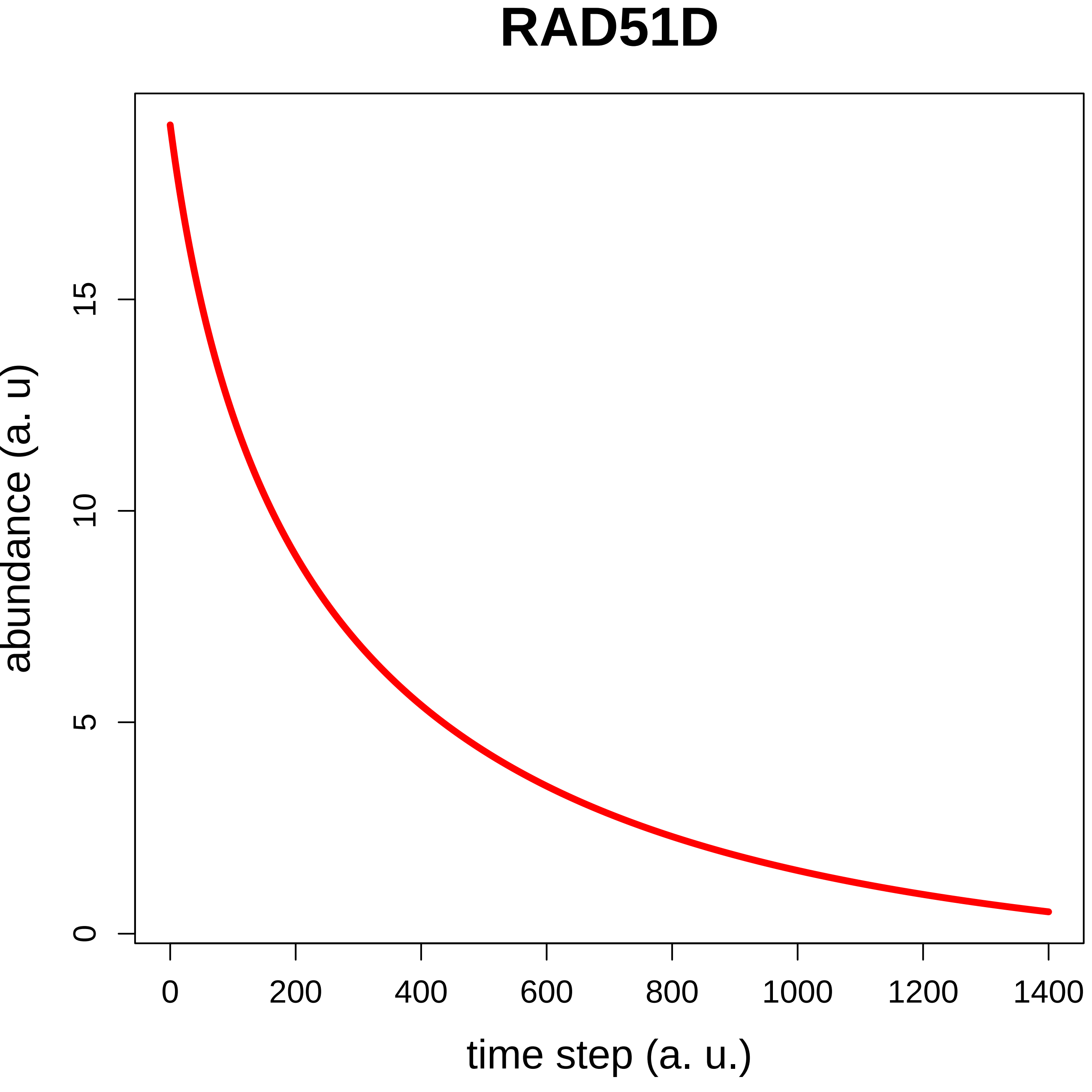
RAD51D
abundance (a. u)
time step (a. u.)
0
5
10
15
0
200
400
600
800
1000
1200
1400

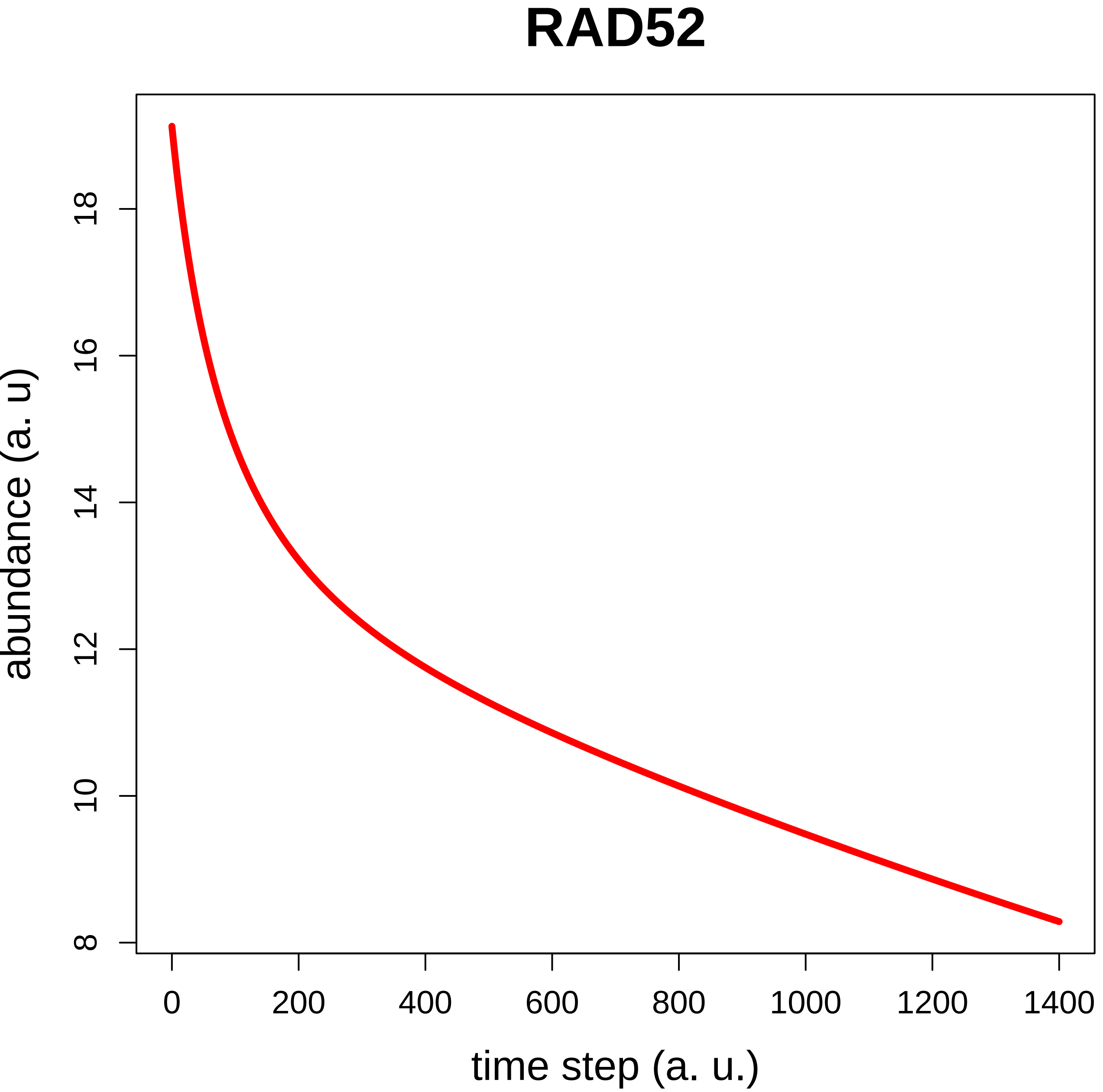

RAD52
abundance (a. u)
18
16
14
12
10
8
0
200
400
600
800
1000
1200
1400
time step (a. u.)

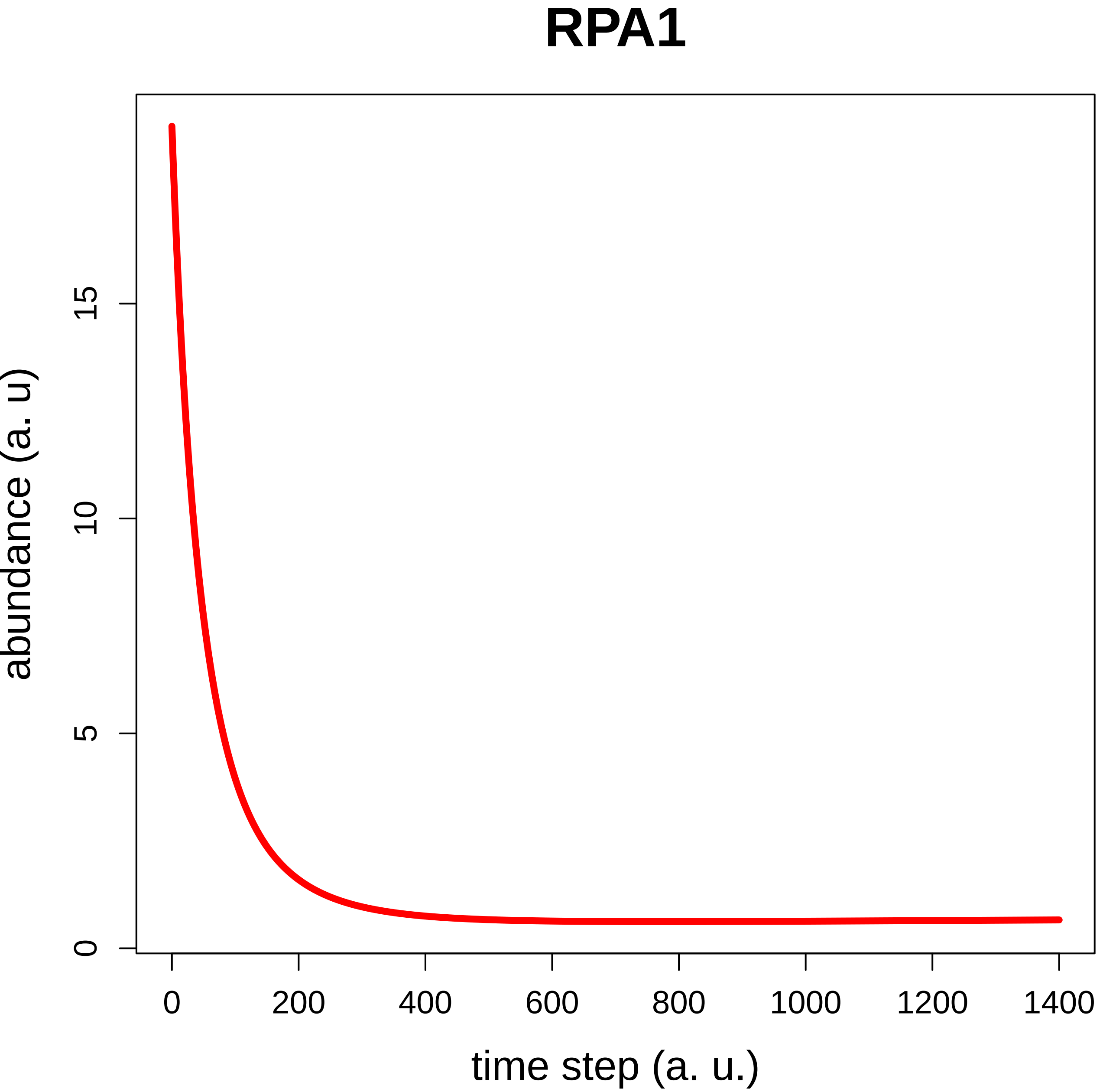
RPA1
abundance (a. u)
0
5
10
15
0
200
400
600
800
1000
1200
1400
time step (a. u.)

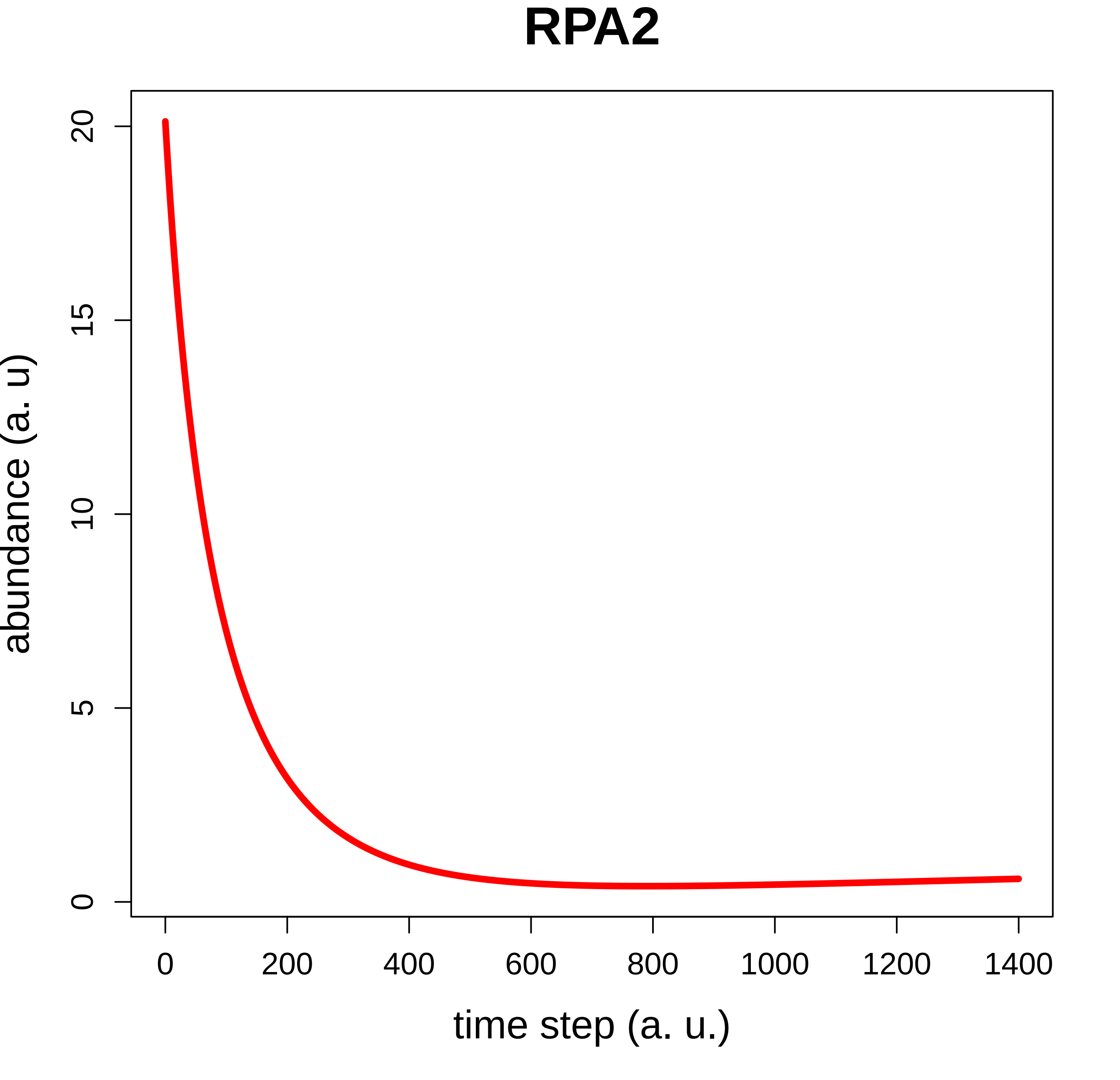

RPA2
abundance (a. u)
time step (a. u.)
0
5
10
15
20
0
200
400
600
800
1000
1200
1400

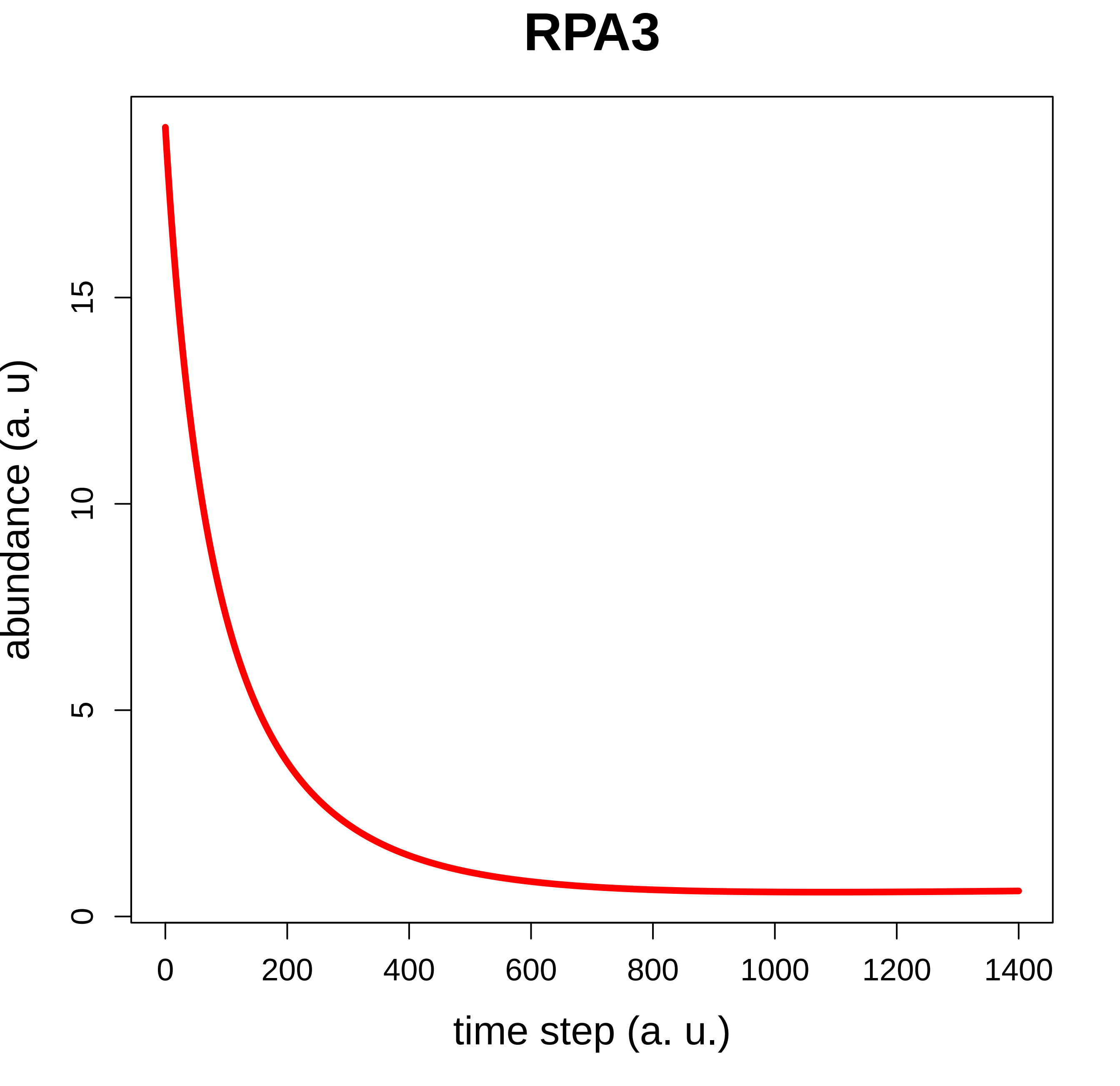

RPA3
abundance (a. u)
time step (a. u.)
0
5
10
15
0
200
400
600
800
1000
1200
1400

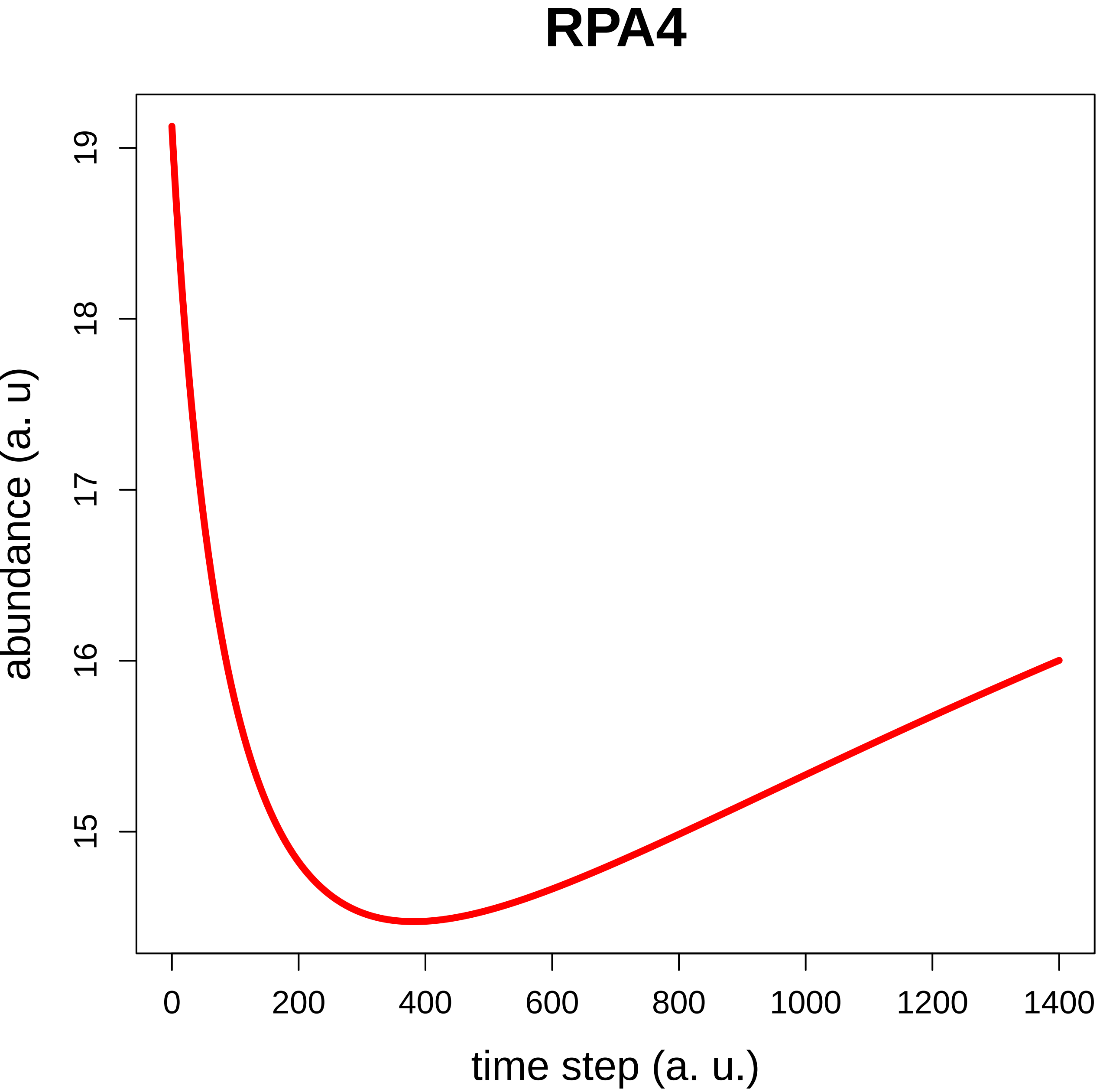

RPA4
abundance (a. u)
19
18
17
16
15
0
200
400
600
800
1000
1200
1400
time step (a. u.)

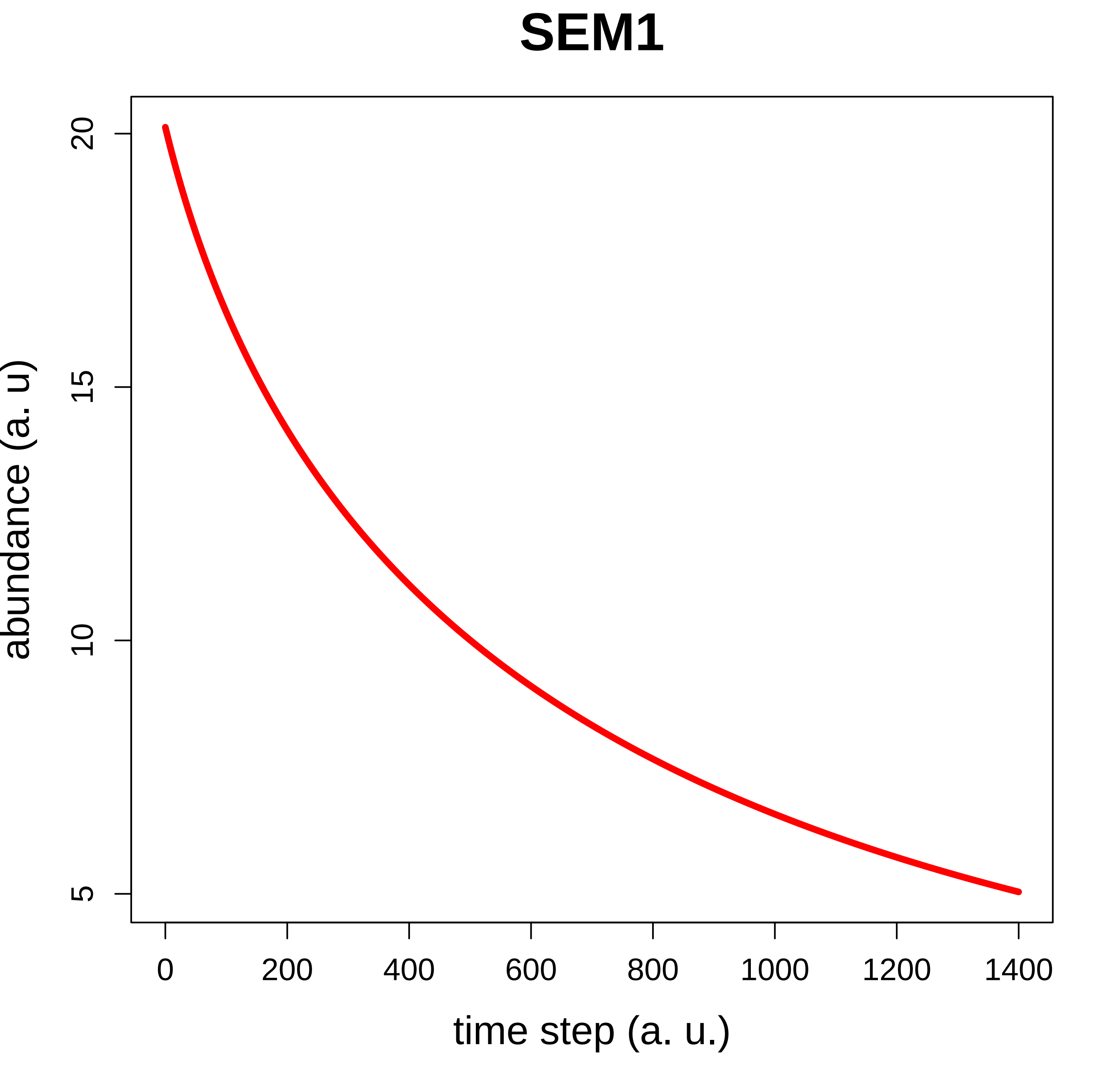
SEM1
abundance (a. u)
20
15
10
5
0
200
400
600
800
1000
1200
1400
time step (a. u.)

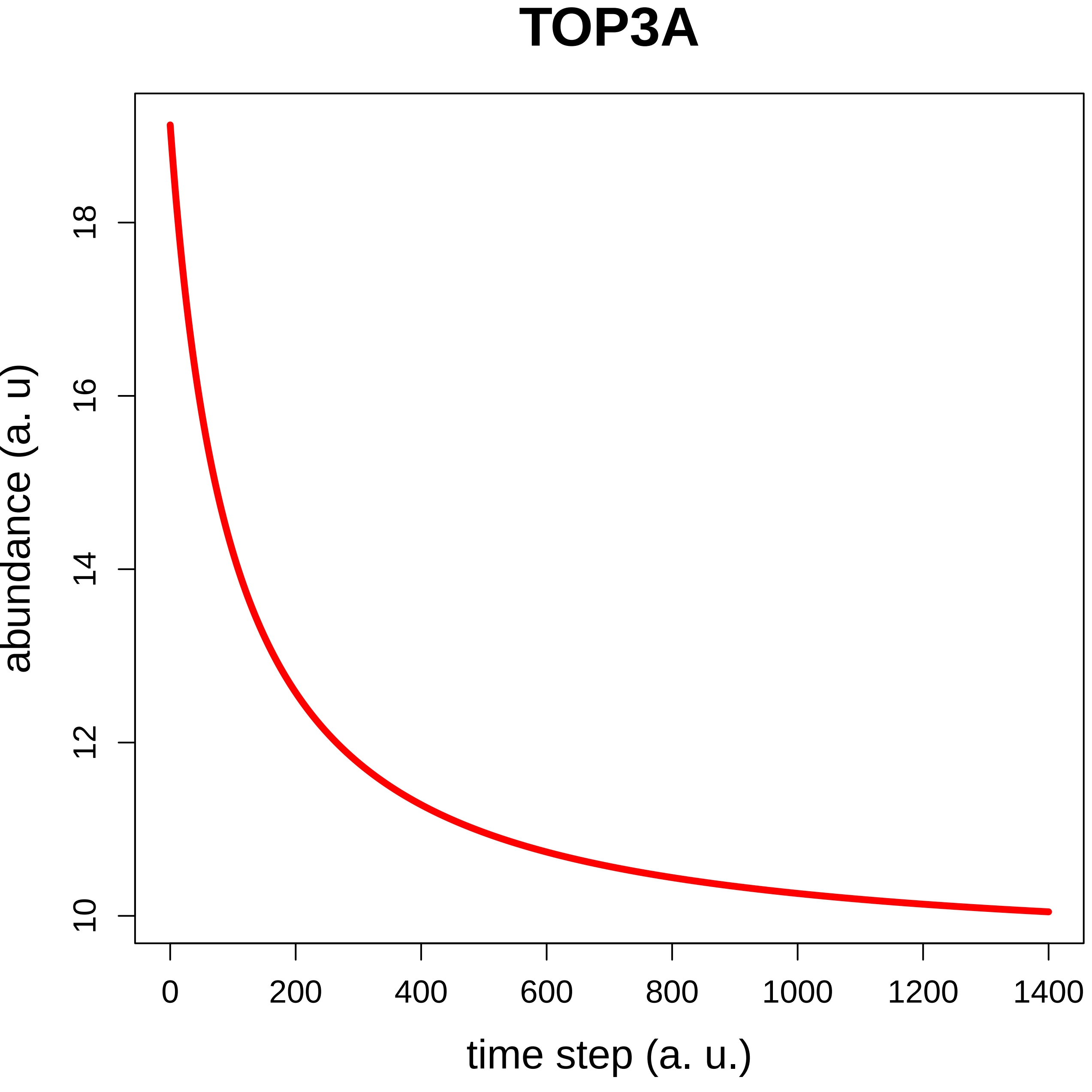

TOP3A
abundance (a. u)
18
16
14
12
10
0
200
400
600
800
1000
1200
1400
time step (a. u.)

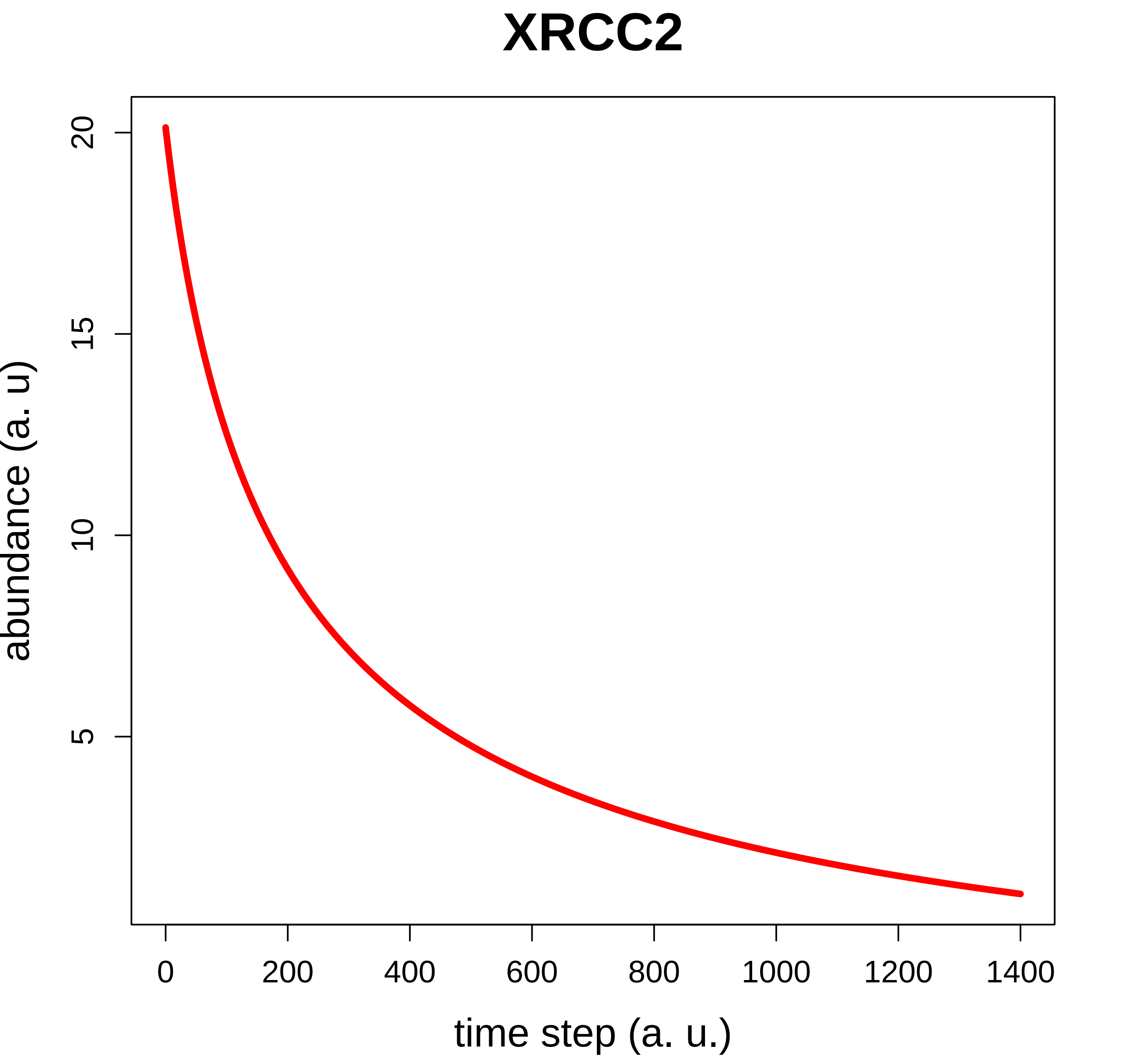

XRCC2
20
15
10
5
abundance (a. u)
0
200
400
600
800
1000
1200
1400
time step (a. u.)

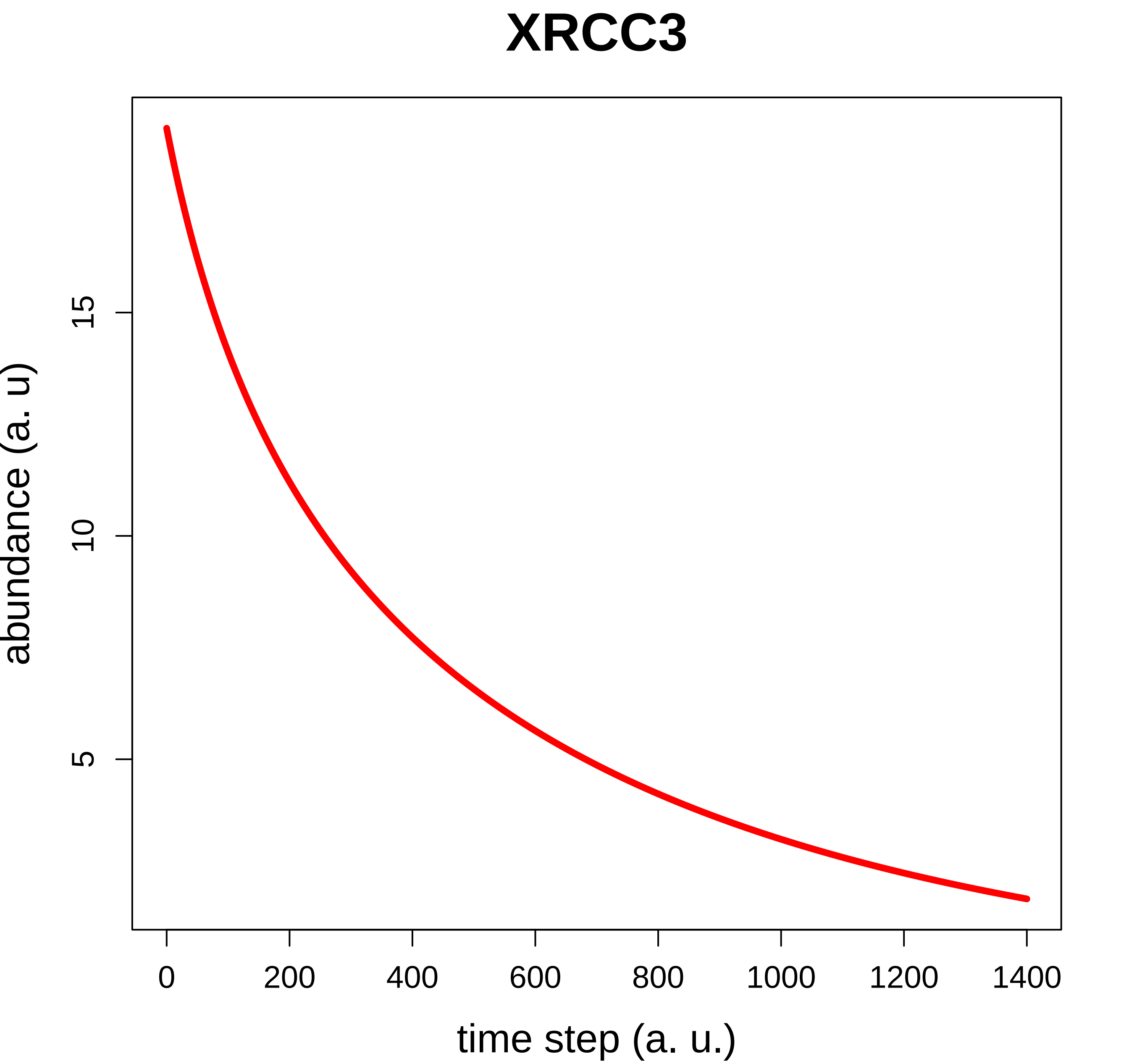
XRCC3
abundance (a. u)
5
10
15
0
200
400
600
800
1000
1200
1400
time step (a. u.)

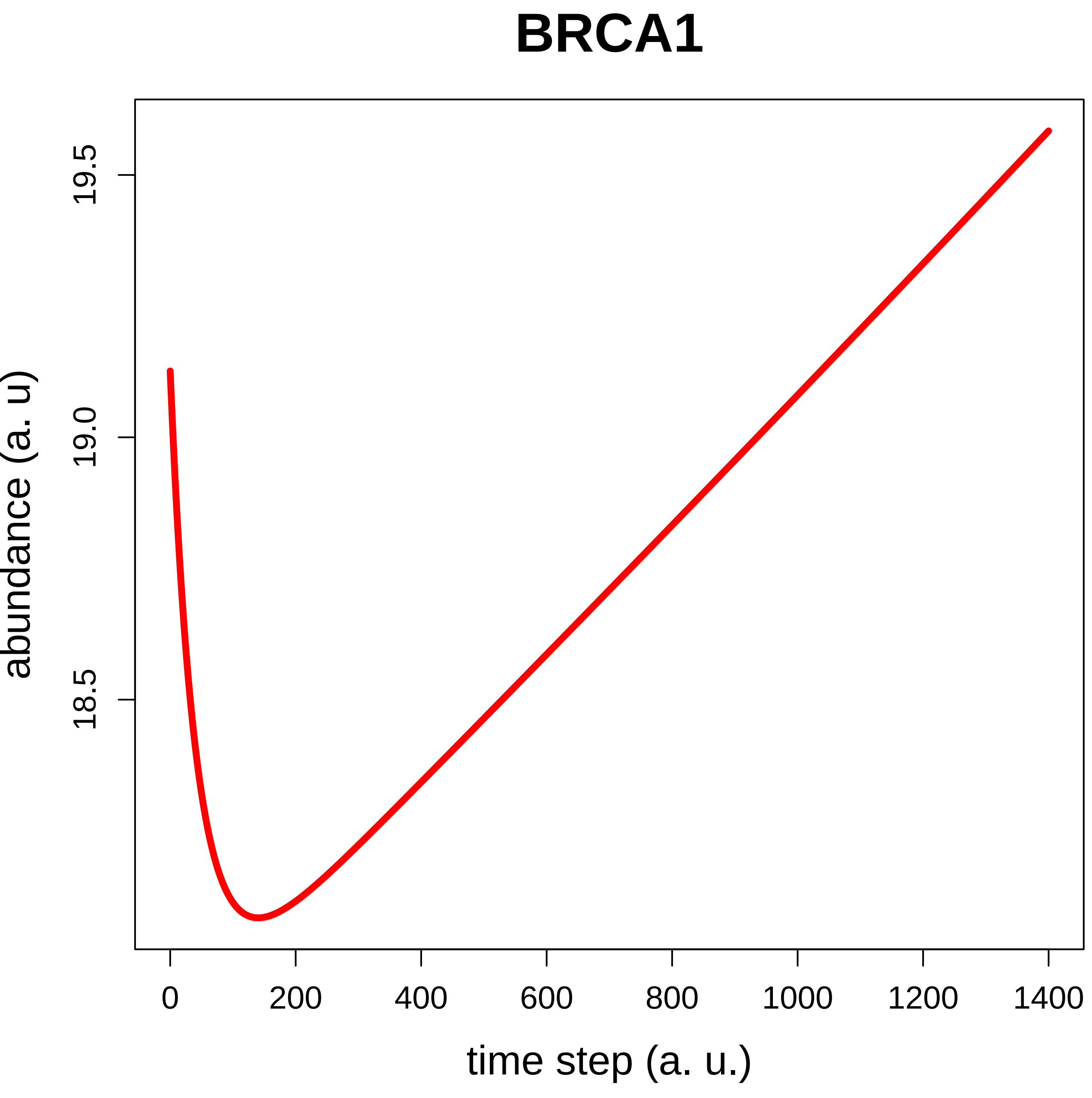
BRCA1
abundance (a. u)
19.5
19.0
18.5
0
200
400
600
800
1000
1200
1400
time step (a. u.)

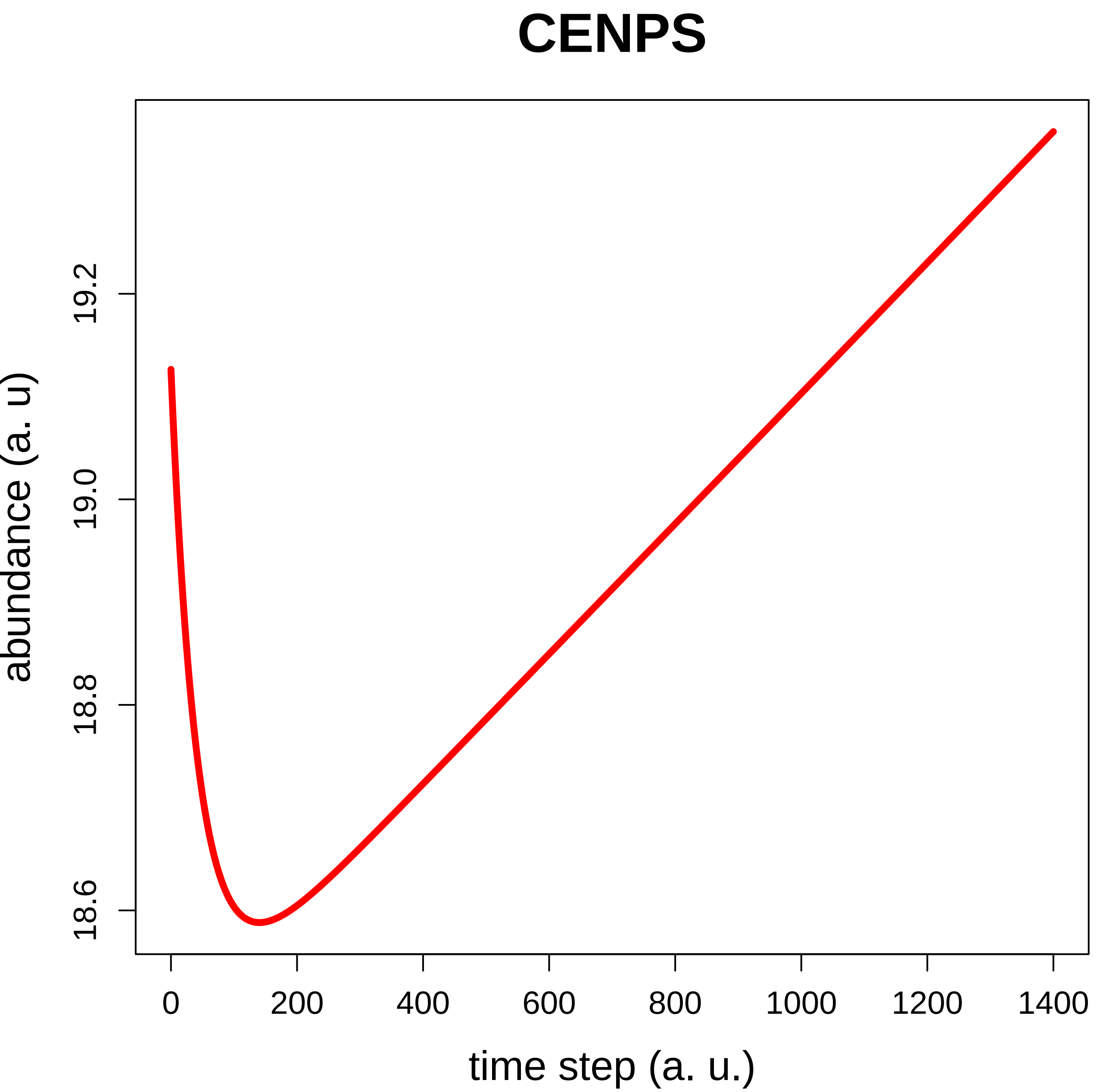
CENPS
abundance (a. u)
19.2
19.0
18.8
18.6
0
200
400
600
800
1000
1200
1400
time step (a. u.)

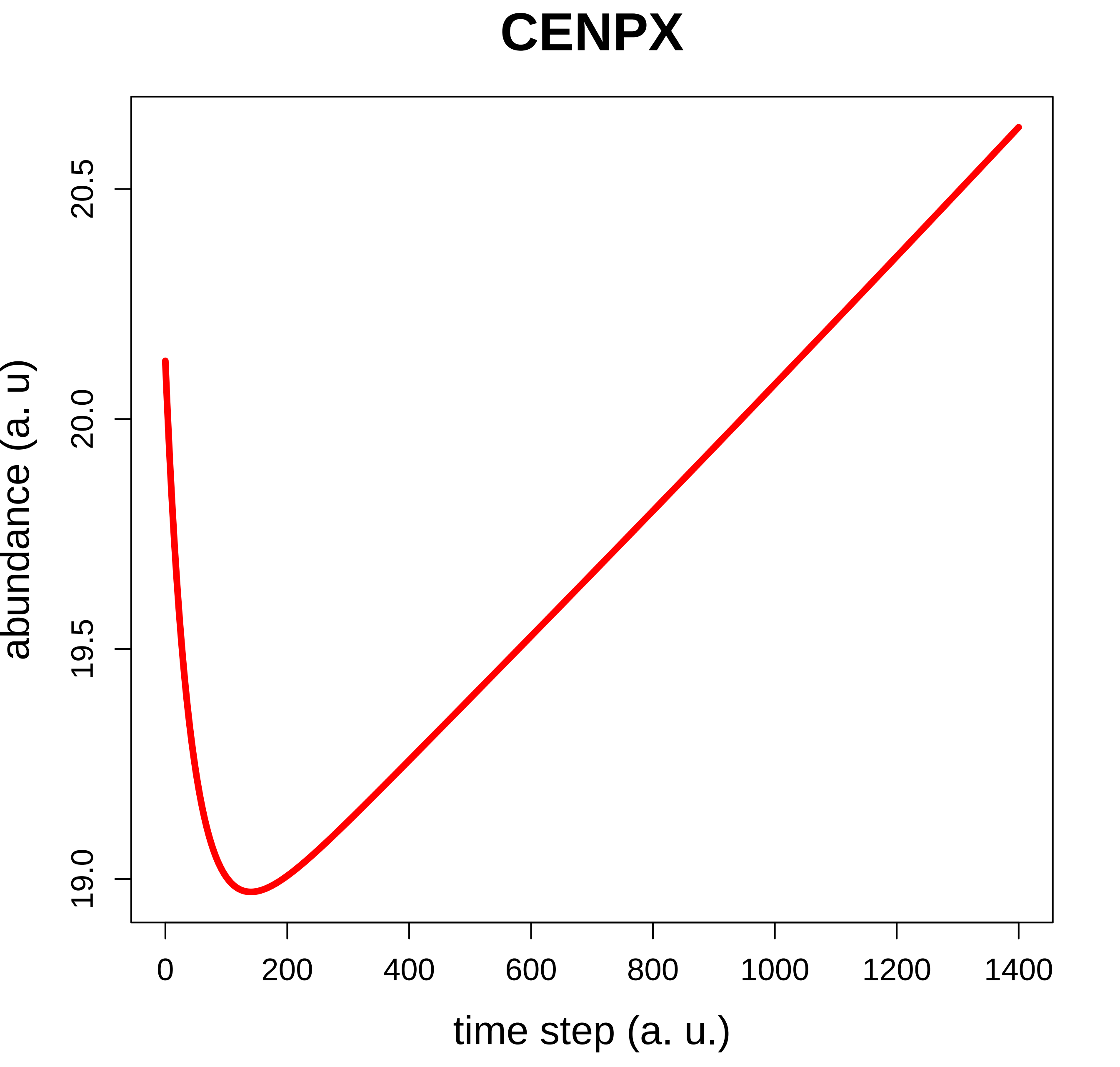

CENPX
20.5
20.0
19.5
19.0
abundance (a. u)
0
200
400
600
800
1000
1200
1400
time step (a. u.)

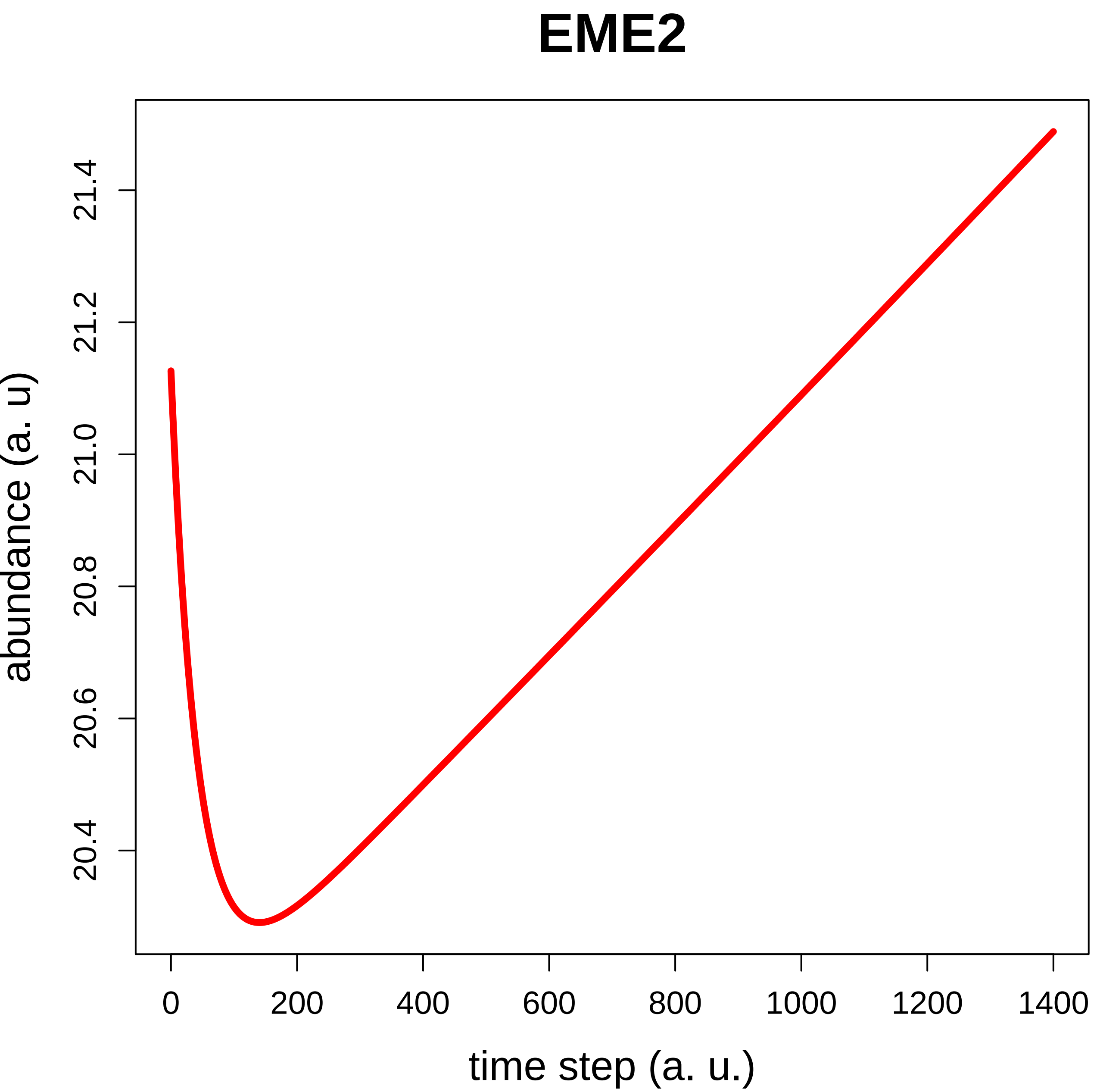
EME2
abundance (a. u)
time step (a. u.)
20.4
20.6
20.8
21.0
21.2
21.4
0
200
400
600
800
1000
1200
1400

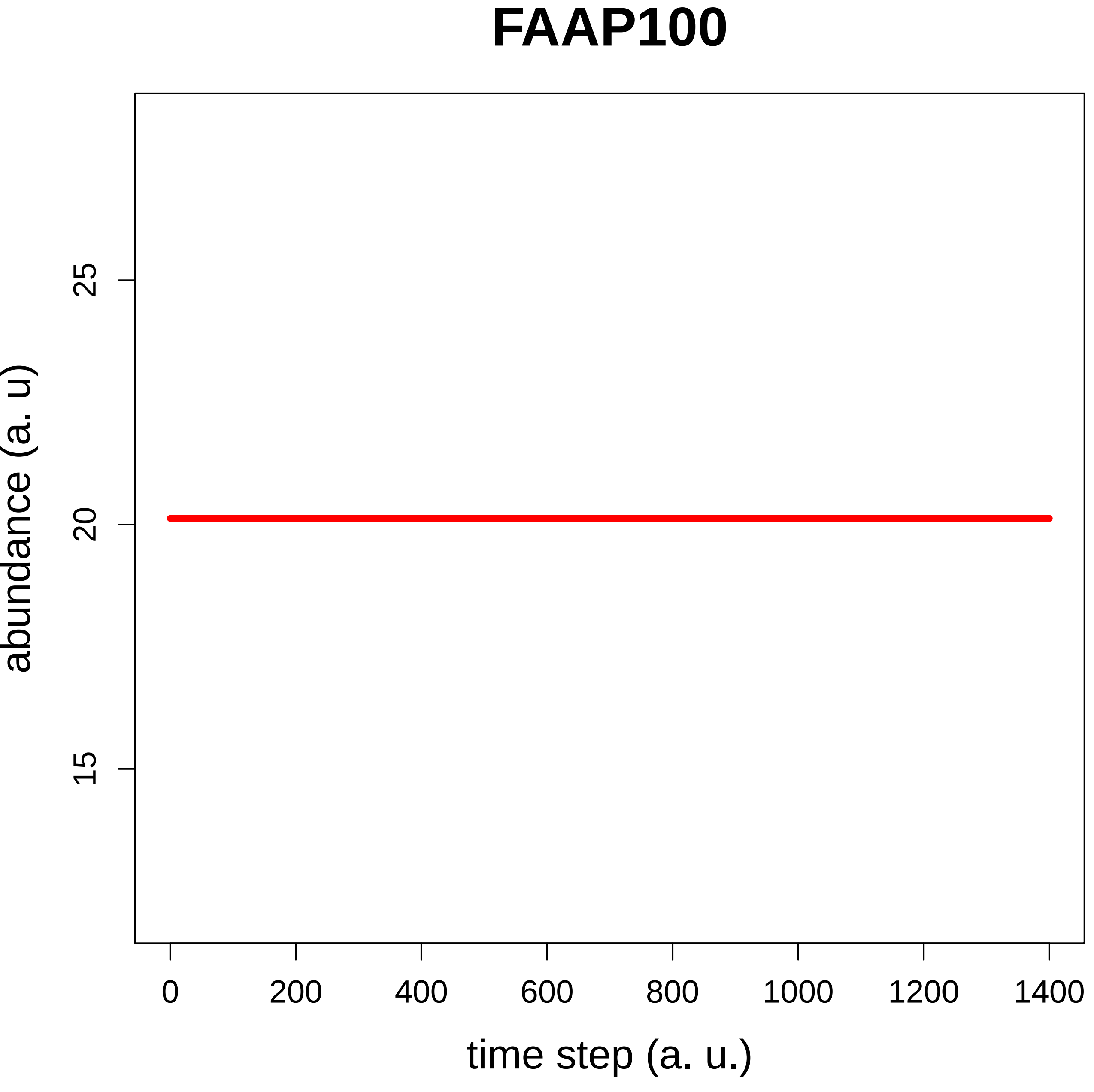

FAAP100
abundance (a. u)
25
20
15
0
200
400
600
800
1000
1200
1400
time step (a. u.)

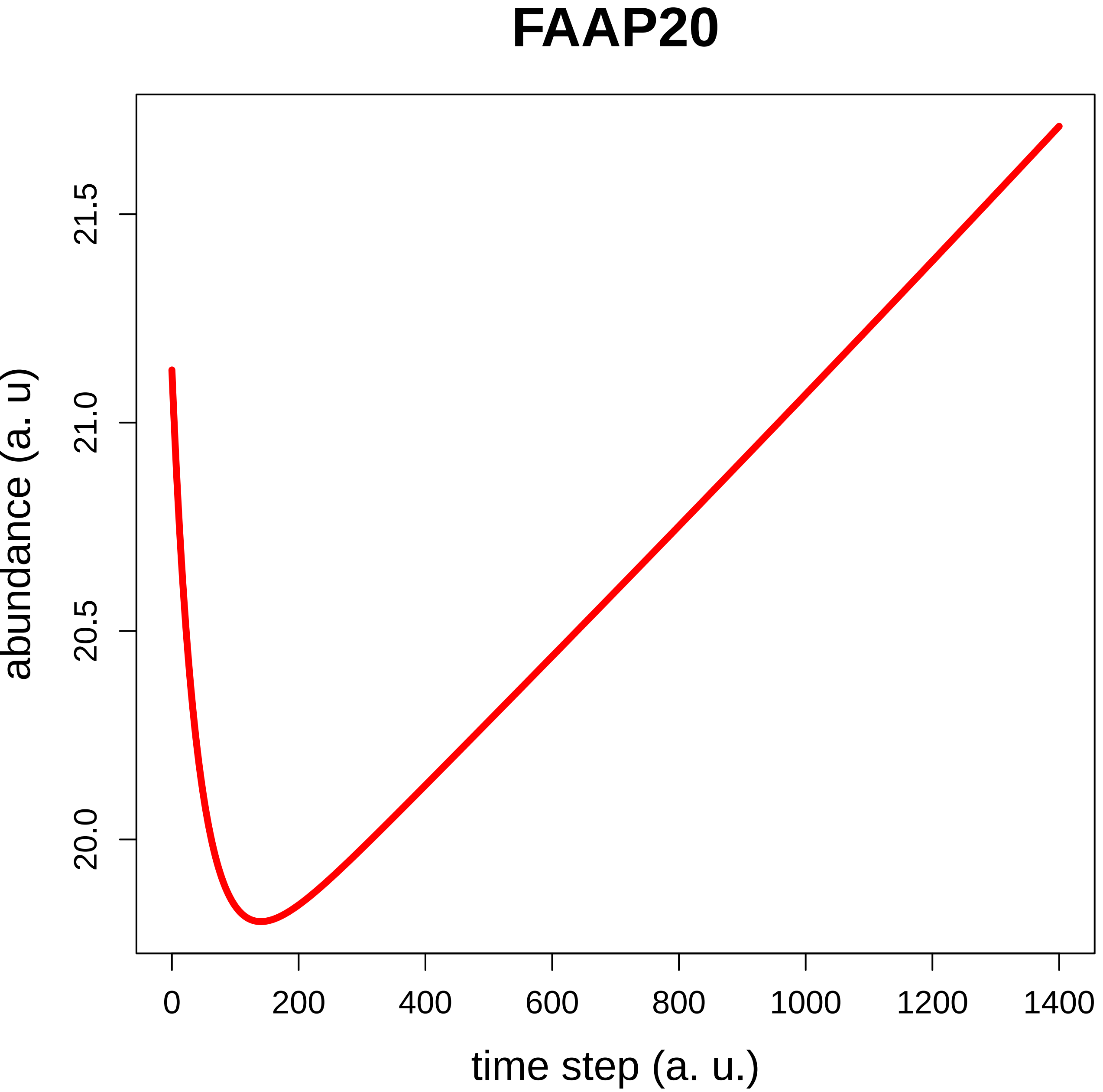

FAAP20
abundance (a. u)
20.0
20.5
21.0
21.5
0
200
400
600
800
1000
1200
1400
time step (a. u.)

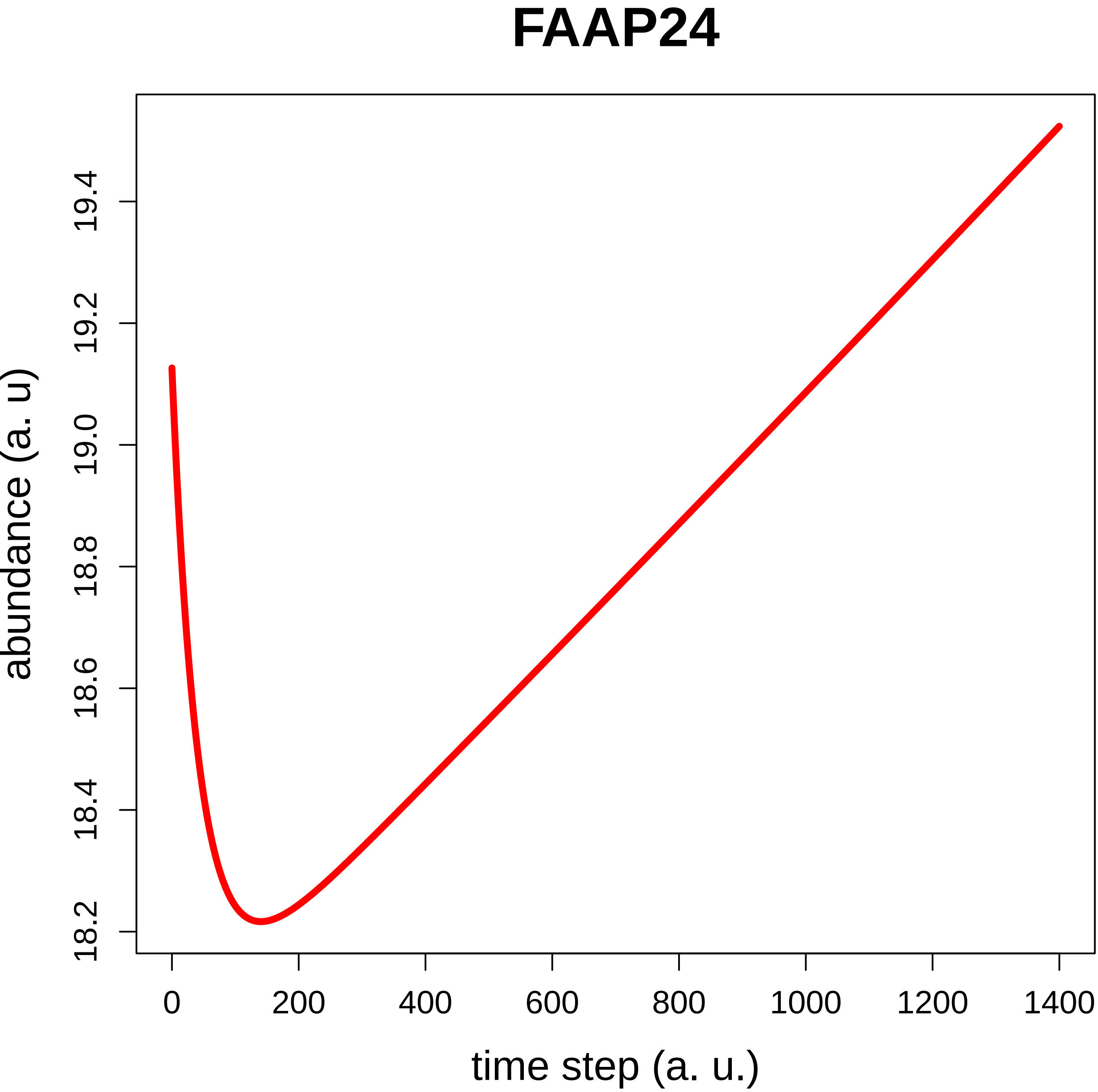

FAAP24
abundance (a. u)
19.4
19.2
19.0
18.8
18.6
18.4
18.2
0
200
400
600
800
1000
1200
1400
time step (a. u.)

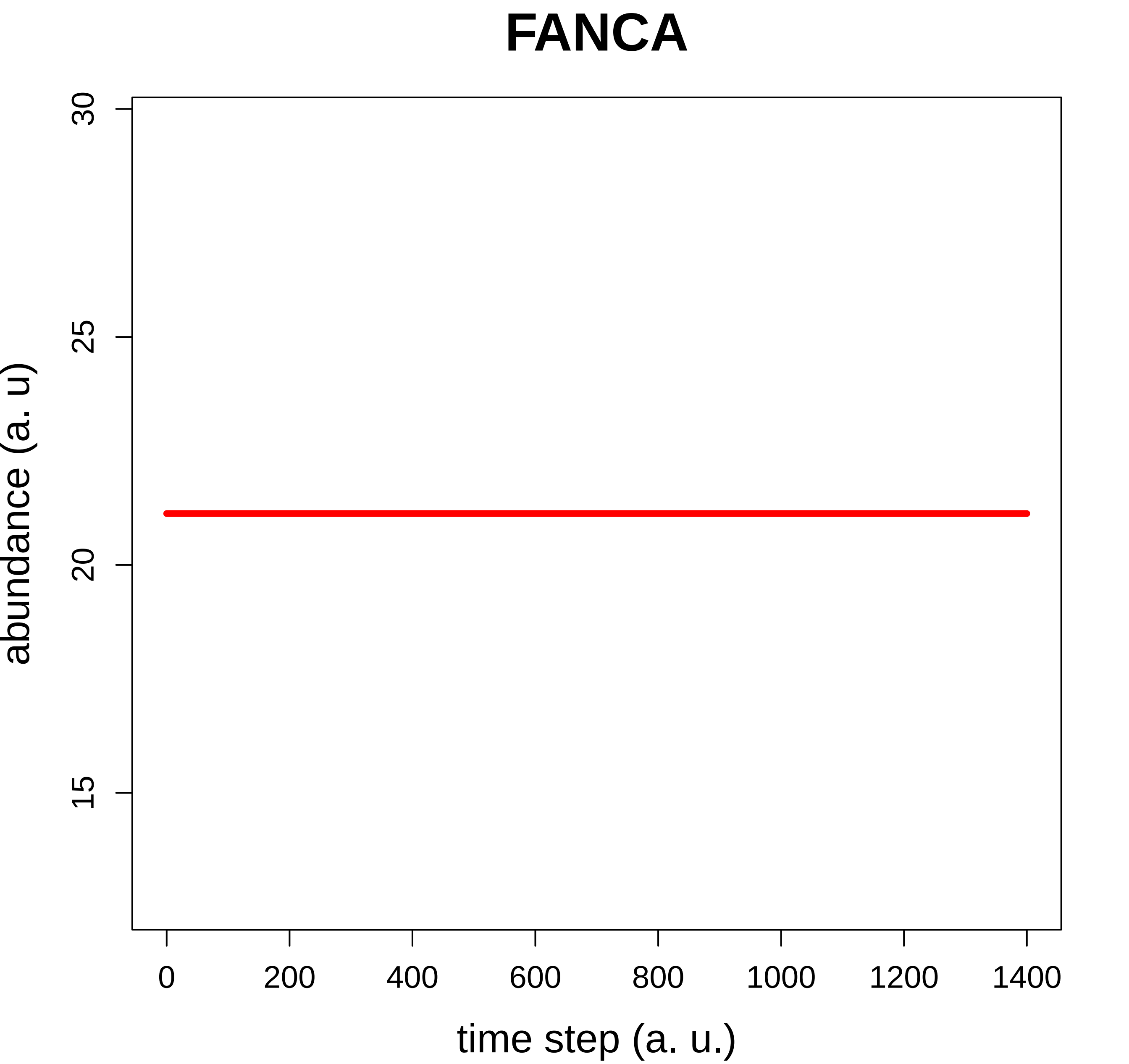

FANCA
abundance (a. u)
time step (a. u.)
30
25
20
15
0
200
400
600
800
1000
1200
1400

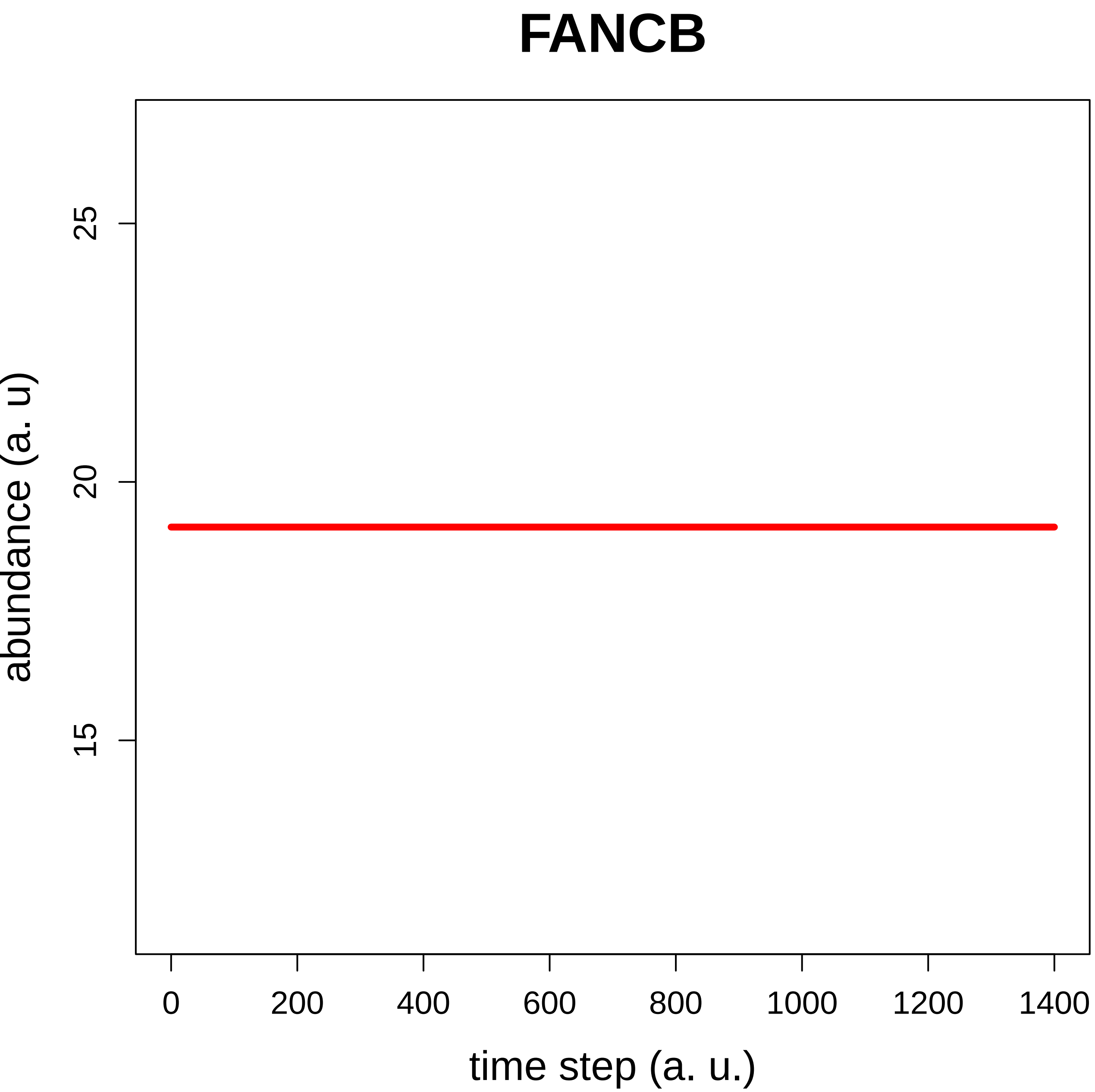

FANCB
25
20
15
abundance (a. u)
0
200
400
600
800
1000
1200
1400
time step (a. u.)

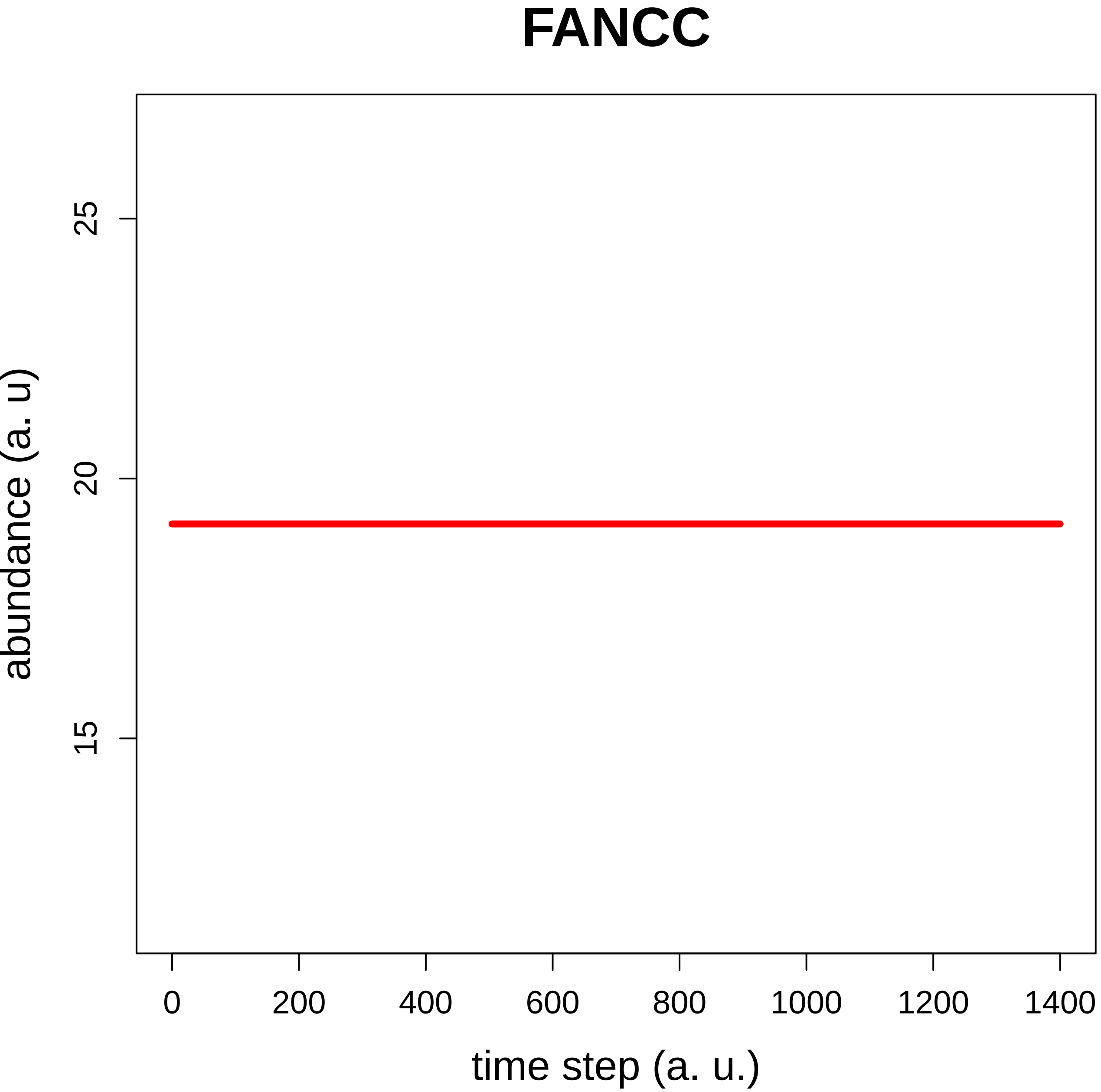
FANCC
25
20
15
abundance (a. u)
0
200
400
600
800
1000
1200
1400
time step (a. u.)

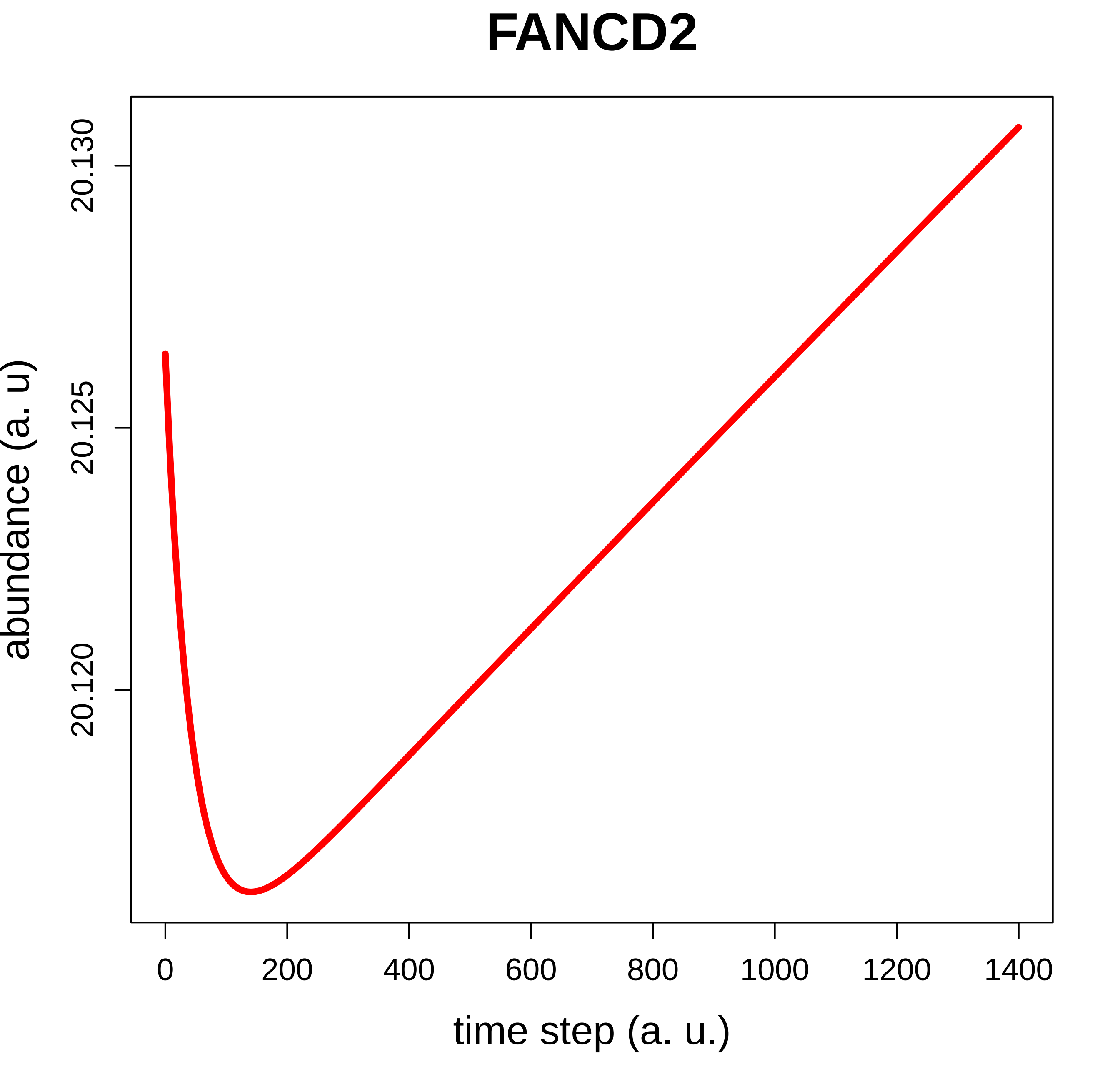
FANCD2
abundance (a. u)
20.130
20.125
20.120
0
200
400
600
800
1000
1200
1400
time step (a. u.)

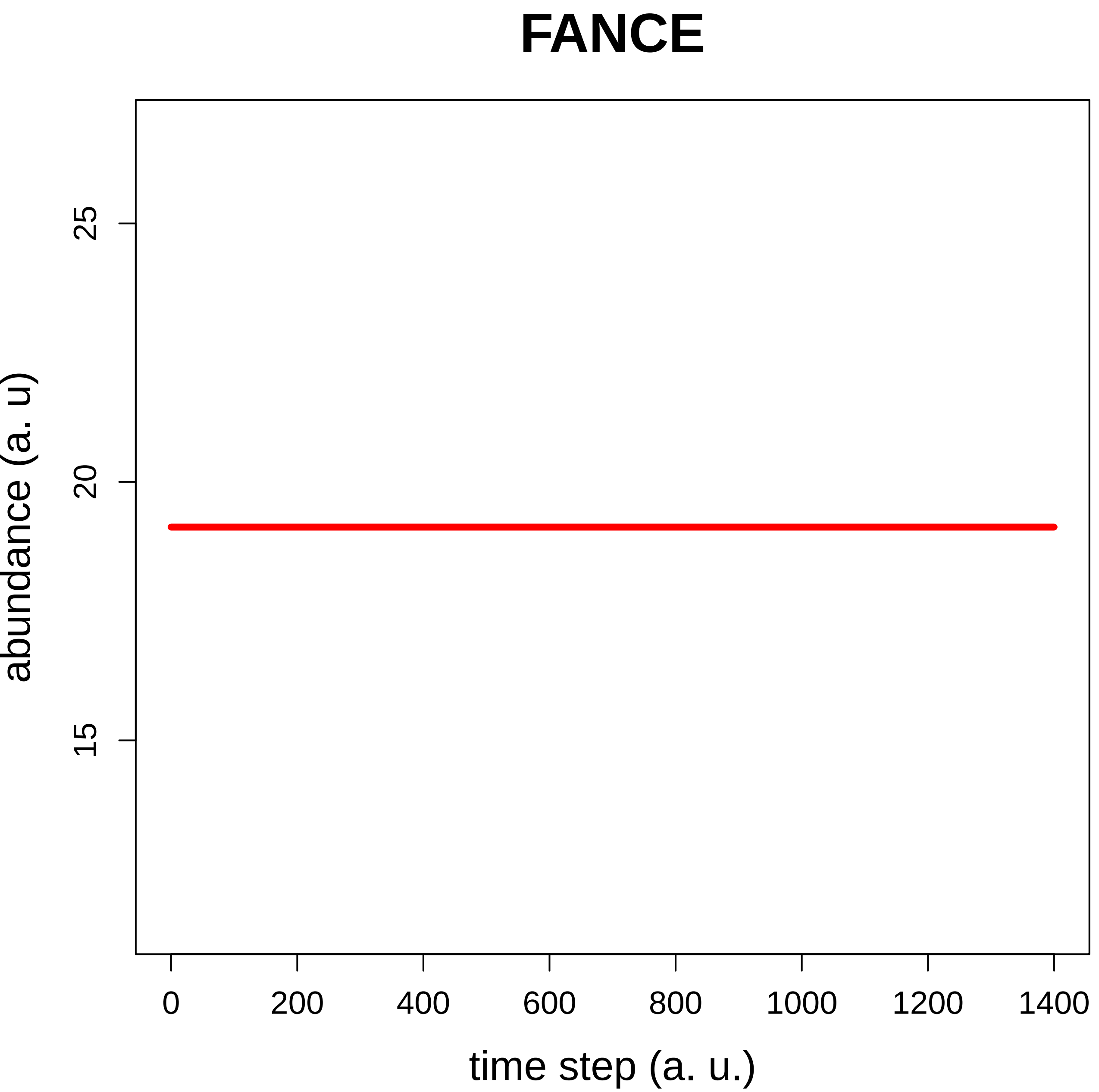
FANCE
abundance (a. u)
25
20
15
0
200
400
600
800
1000
1200
1400
time step (a. u.)

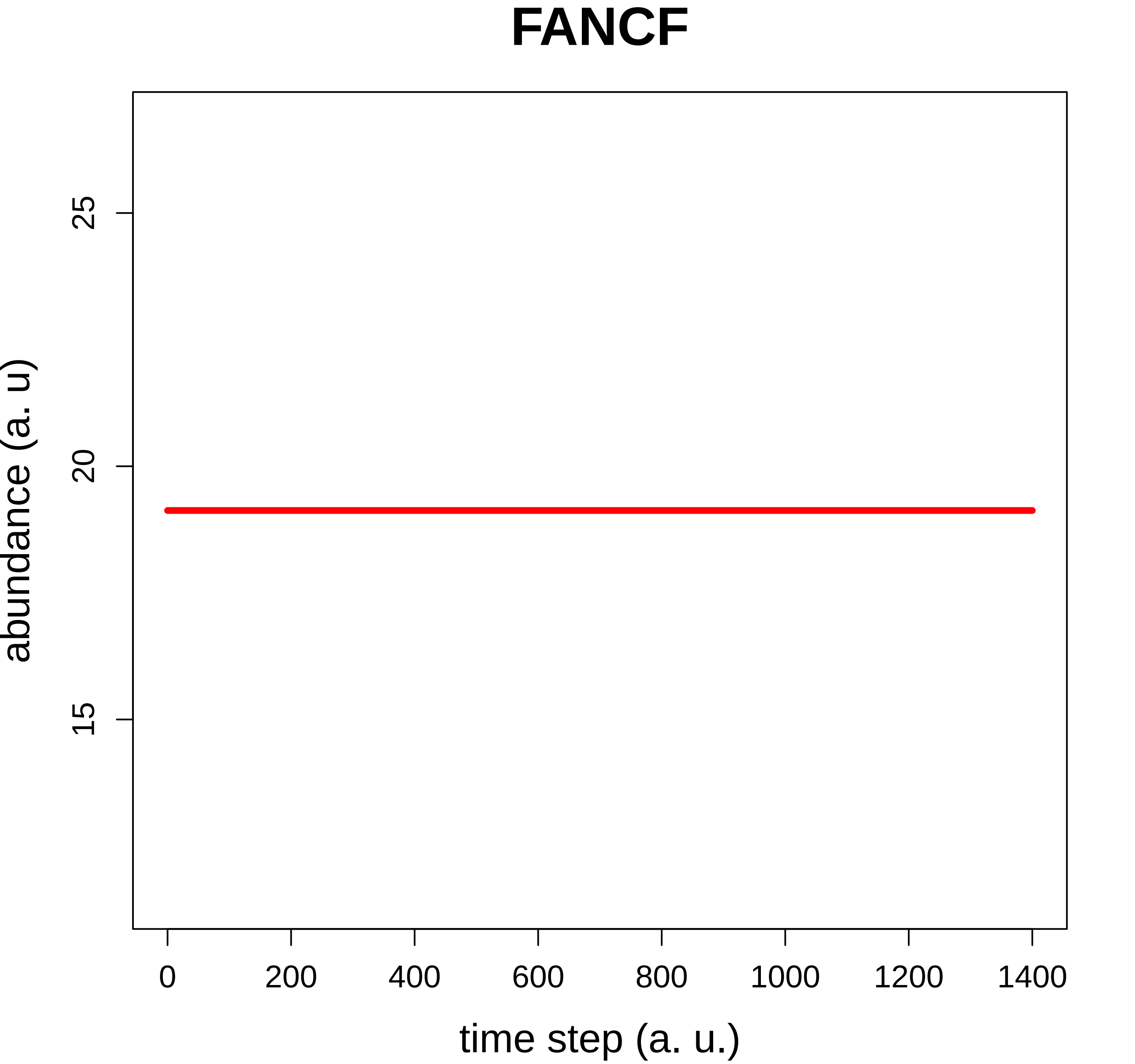
FANCF
abundance (a. u)
25
20
15
0
200
400
600
800
1000
1200
1400
time step (a. u.)

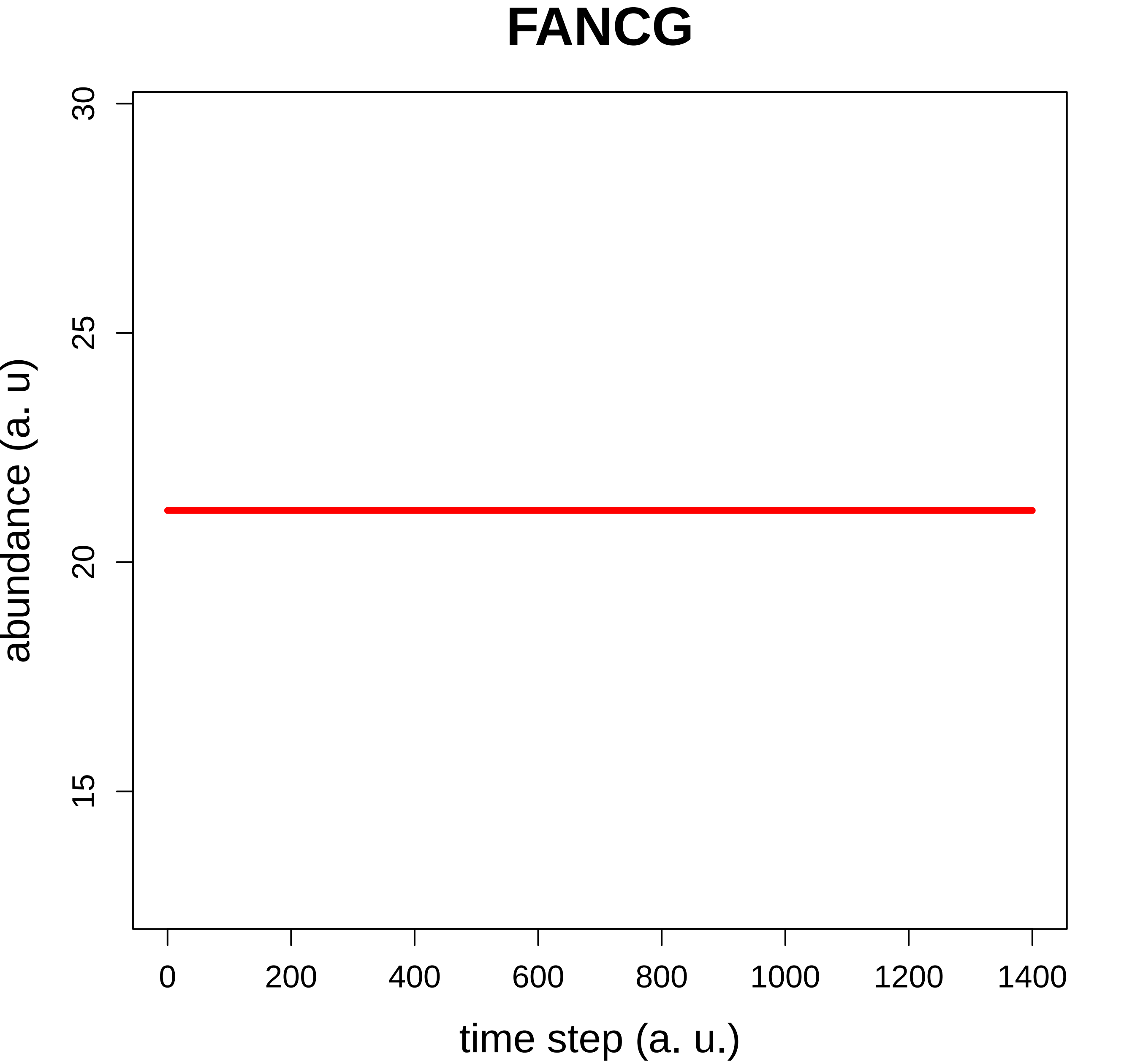
FANCG
abundance (a. u)
time step (a. u.)
15
20
25
30
0
200
400
600
800
1000
1200
1400

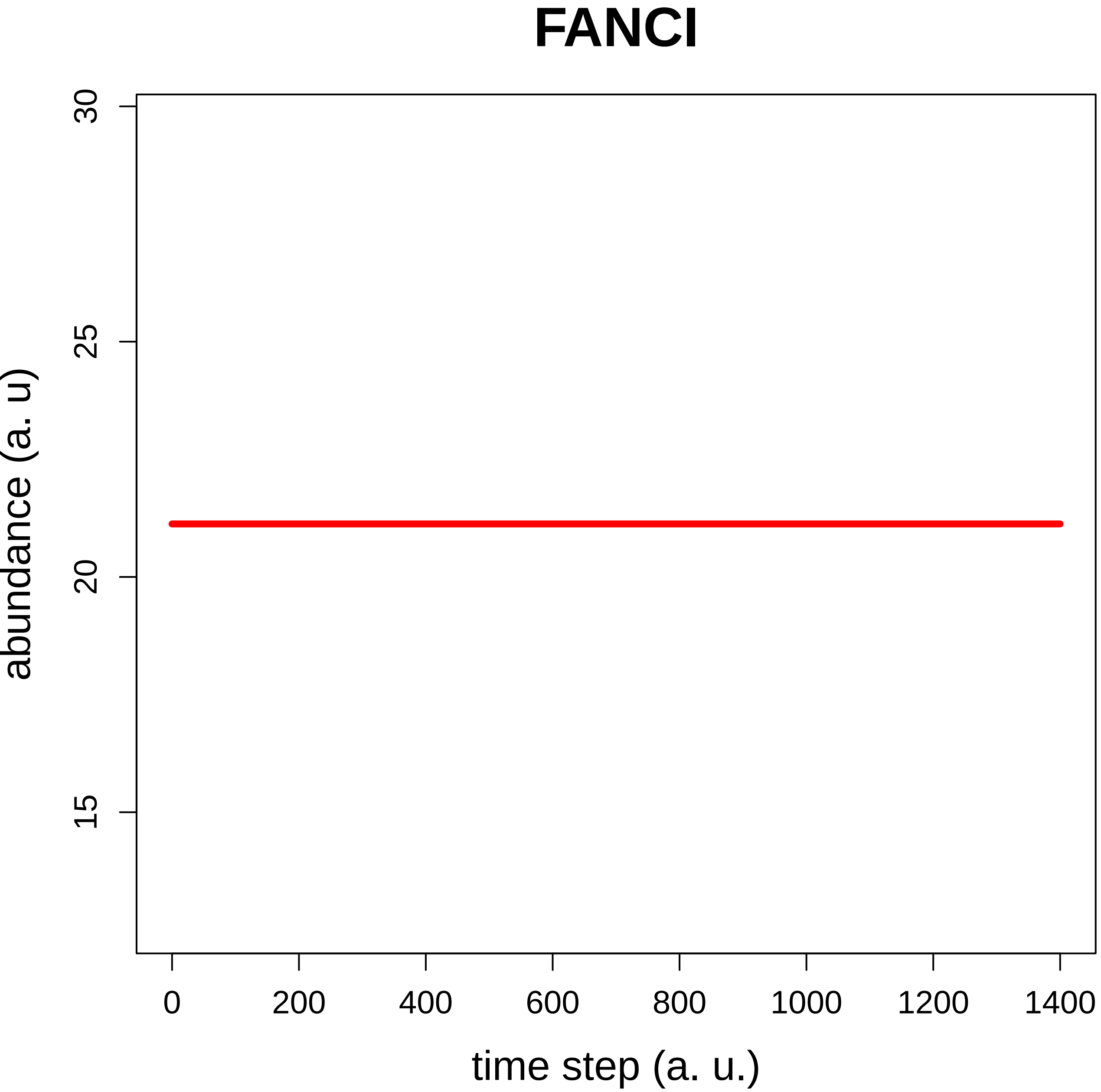

FANCI
abundance (a. u)
30
25
20
15
0
200
400
600
800
1000
1200
1400
time step (a. u.)

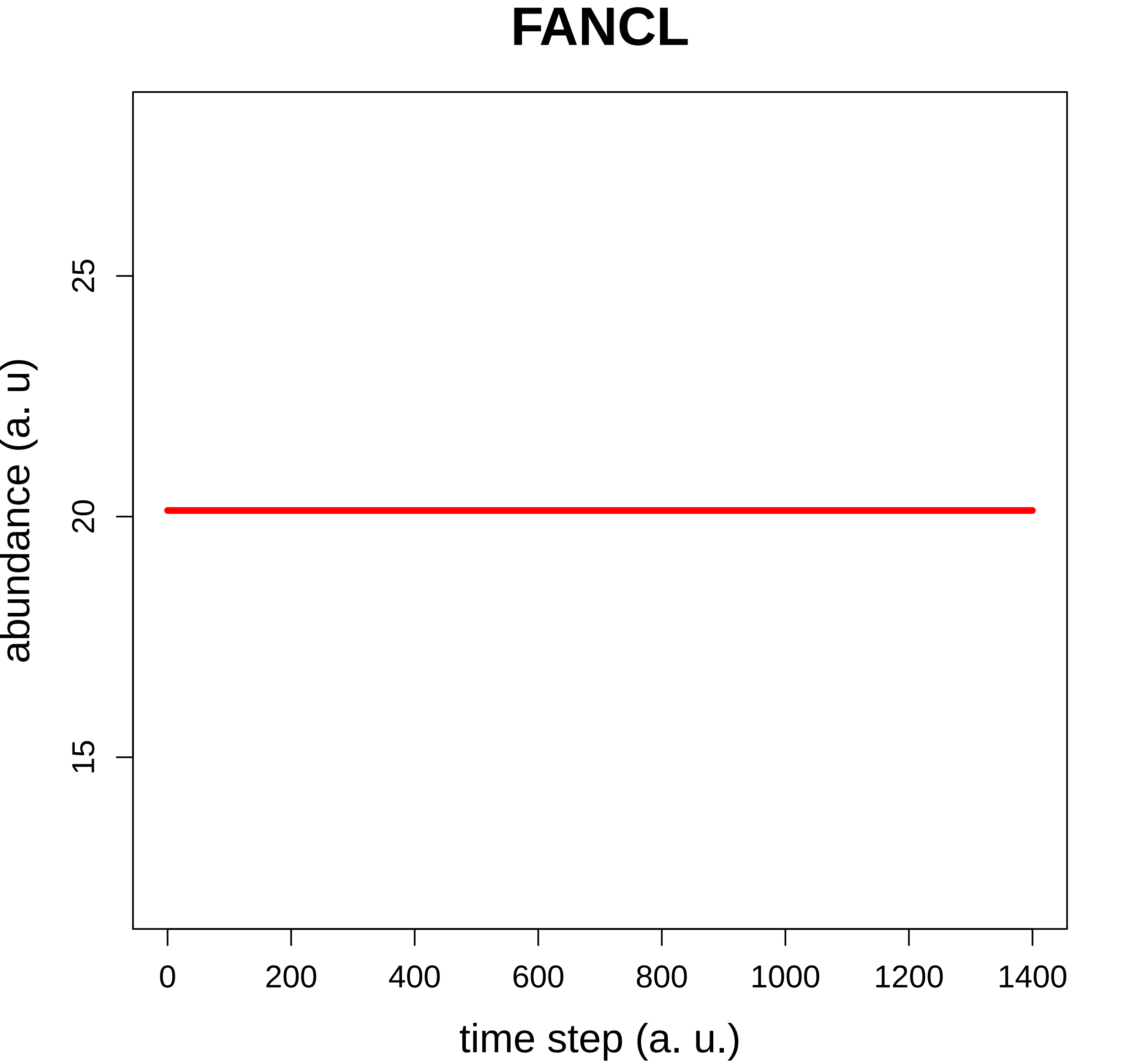

FANCL
abundance (a. u)
25
20
15
0
200
400
600
800
1000
1200
1400
time step (a. u.)

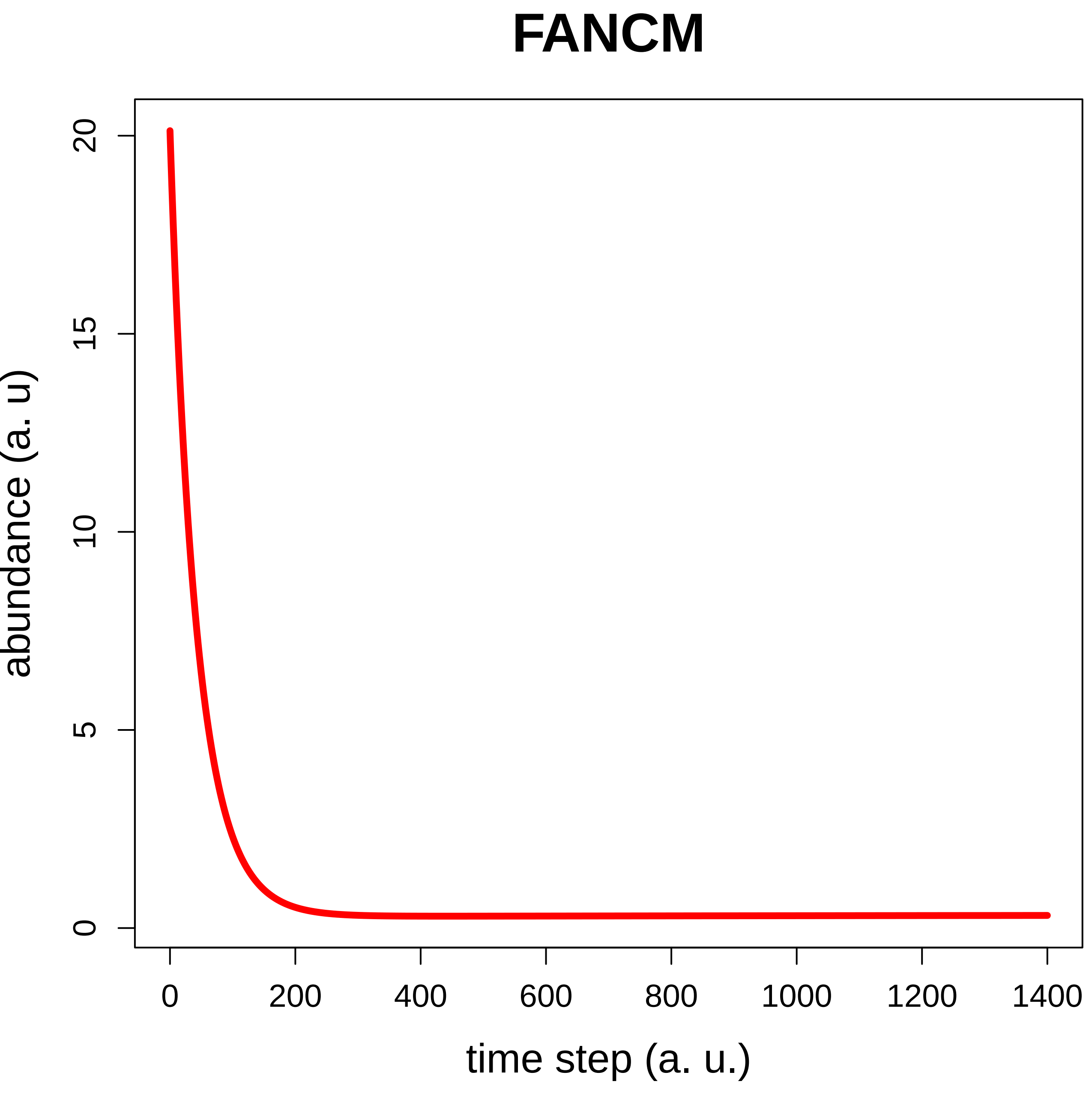
FANCM
abundance (a. u)
20
15
10
5
0
0
200
400
600
800
1000
1200
1400
time step (a. u.)

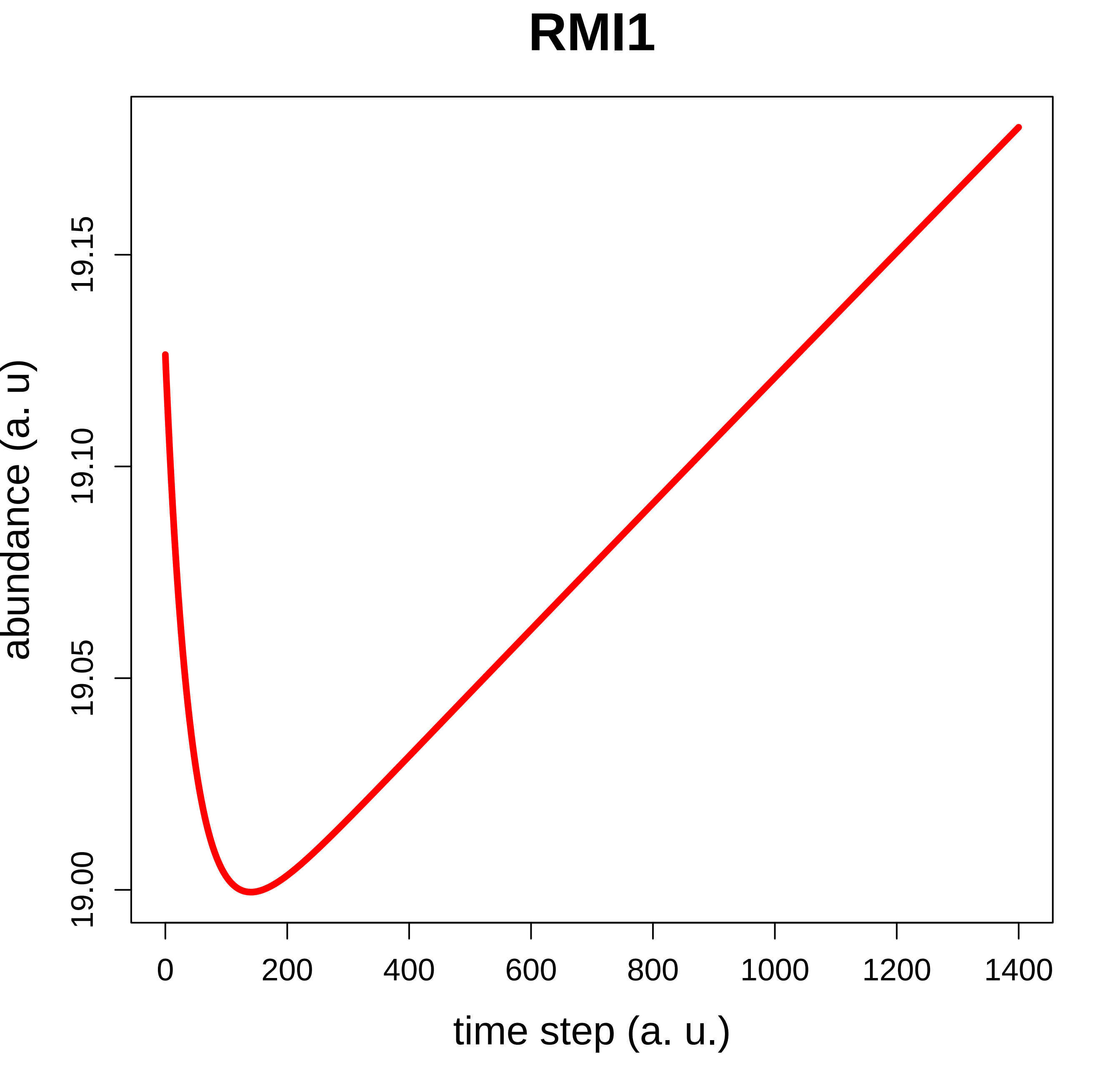

RMI1
abundance (a. u)
19.00
19.05
19.10
19.15
0
200
400
600
800
1000
1200
1400
time step (a. u.)

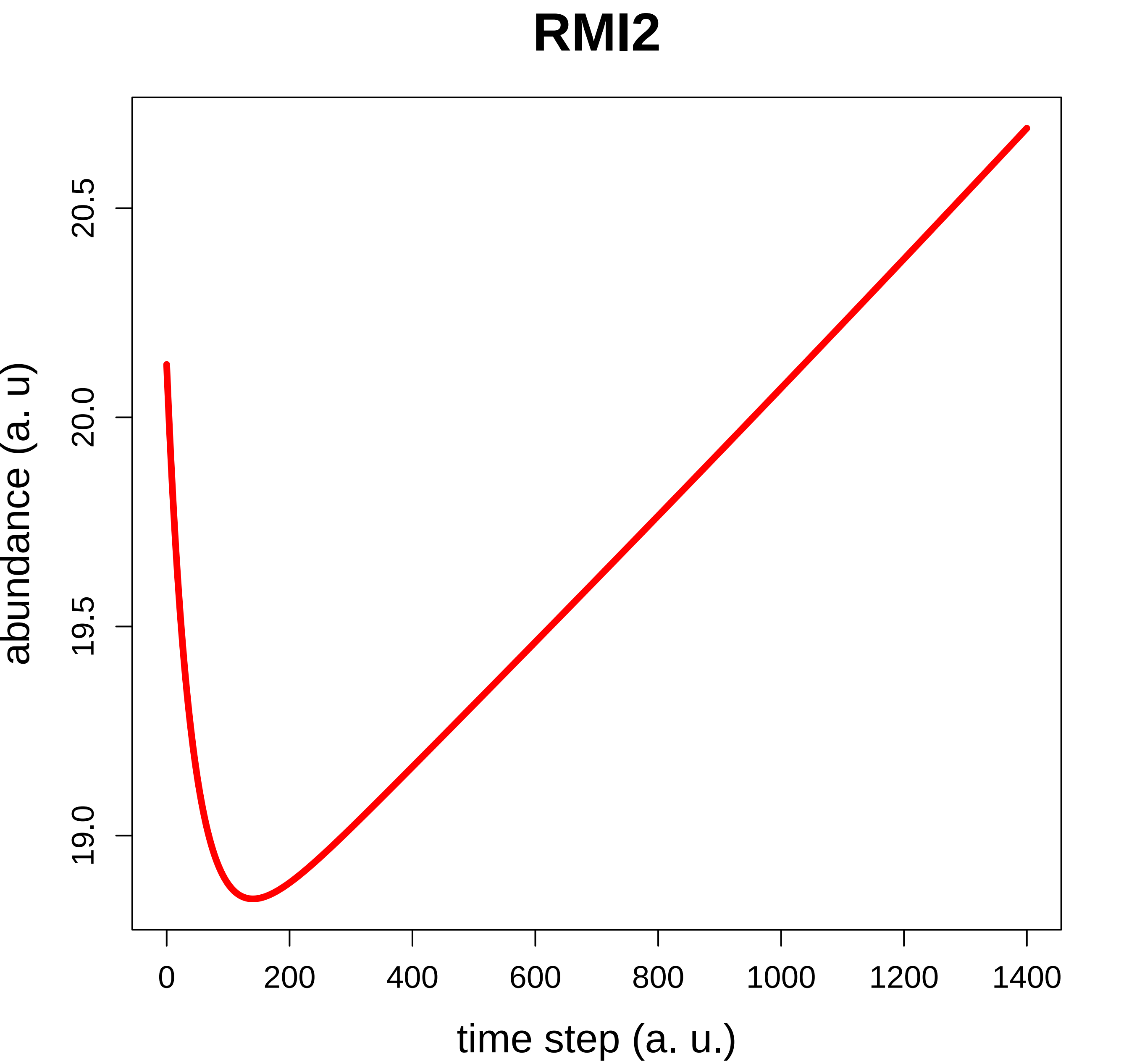
RMI2
abundance (a. u)
20.5
20.0
19.5
19.0
0
200
400
600
800
1000
1200
1400
time step (a. u.)

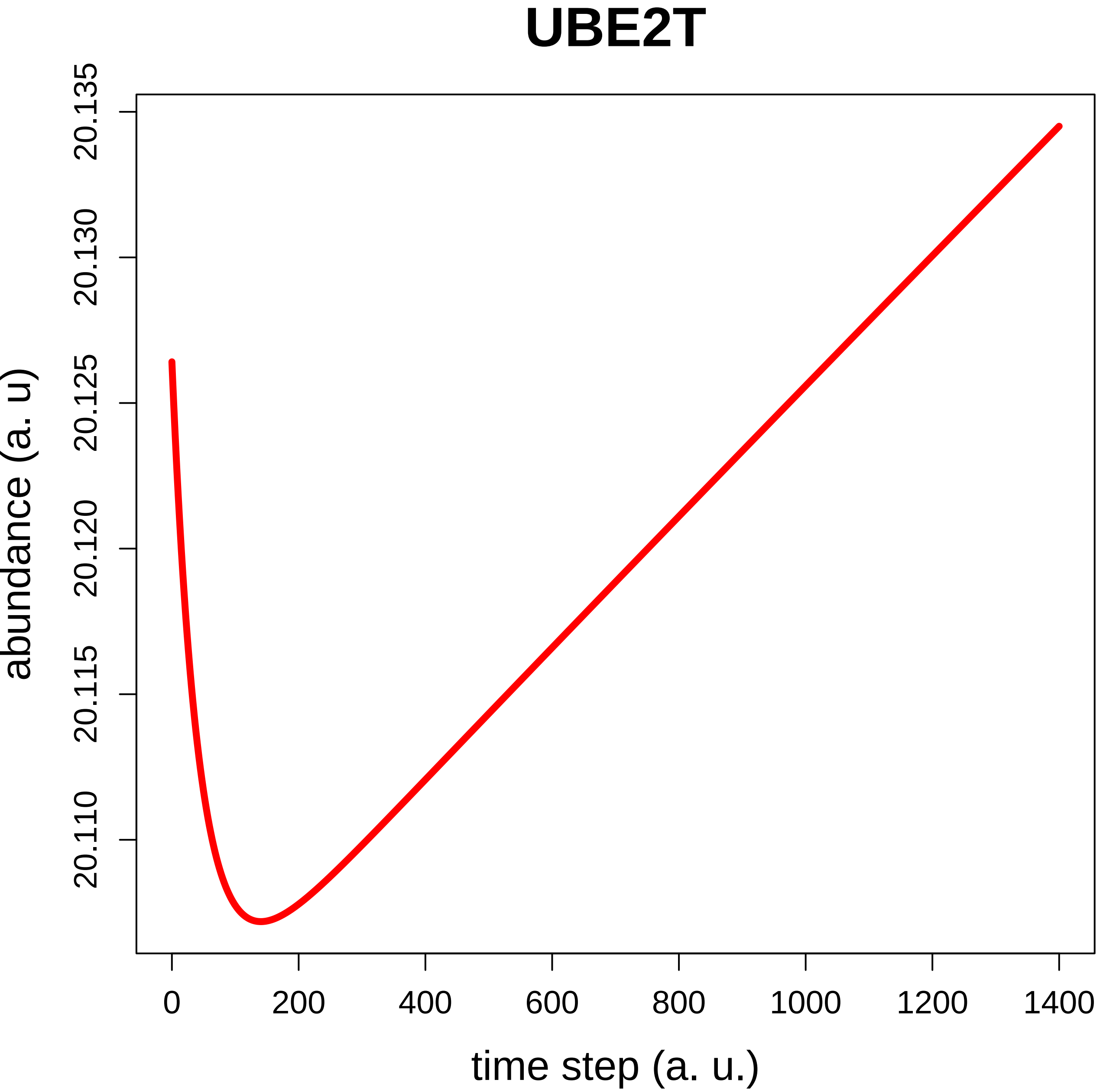

UBE2T
abundance (a. u)
20.110
20.115
20.120
20.125
20.130
20.135
0
200
400
600
800
1000
1200
1400
time step (a. u.)

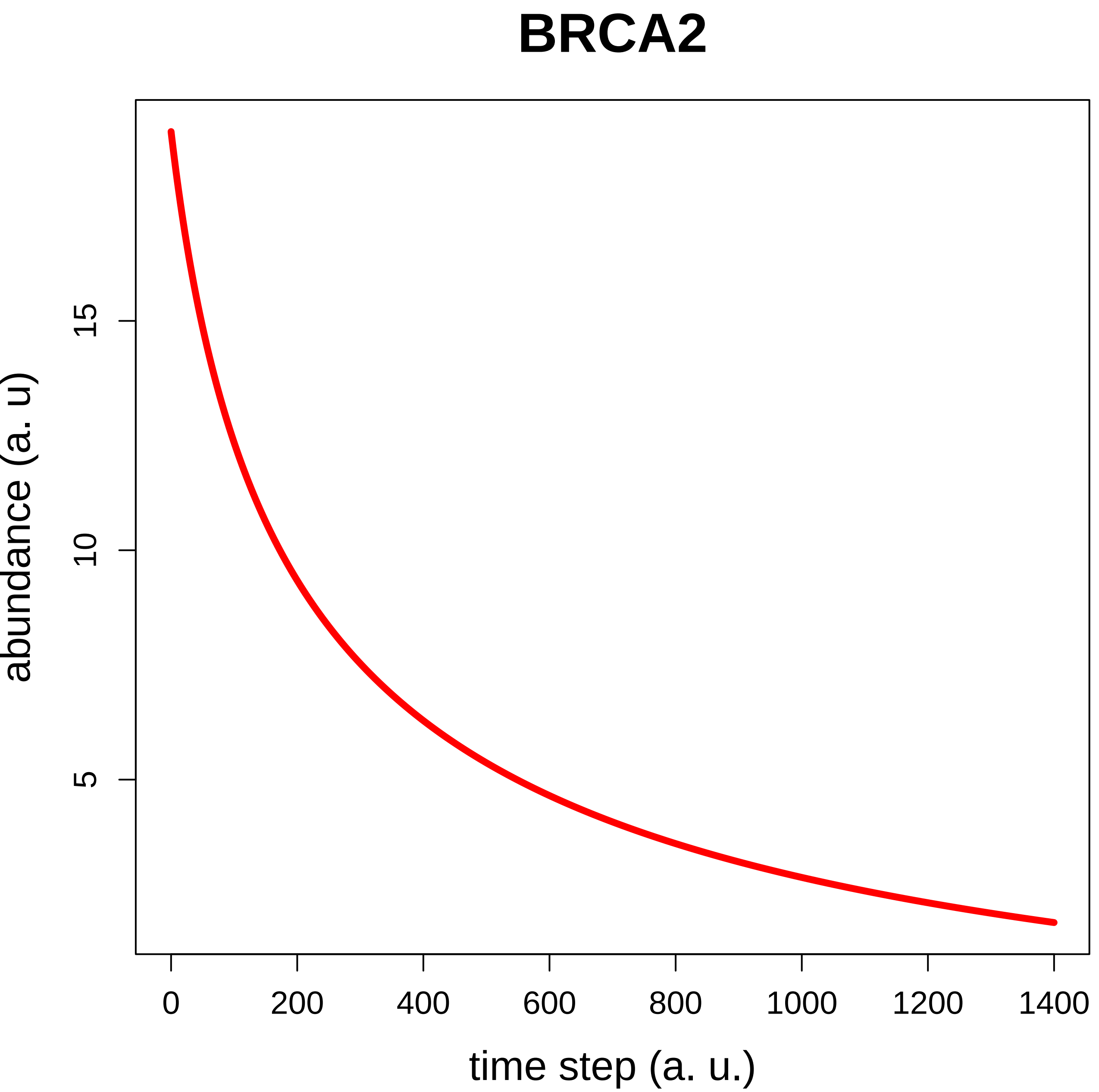
BRCA2
15
10
5
abundance (a. u)
0
200
400
600
800
1000
1200
1400
time step (a. u.)

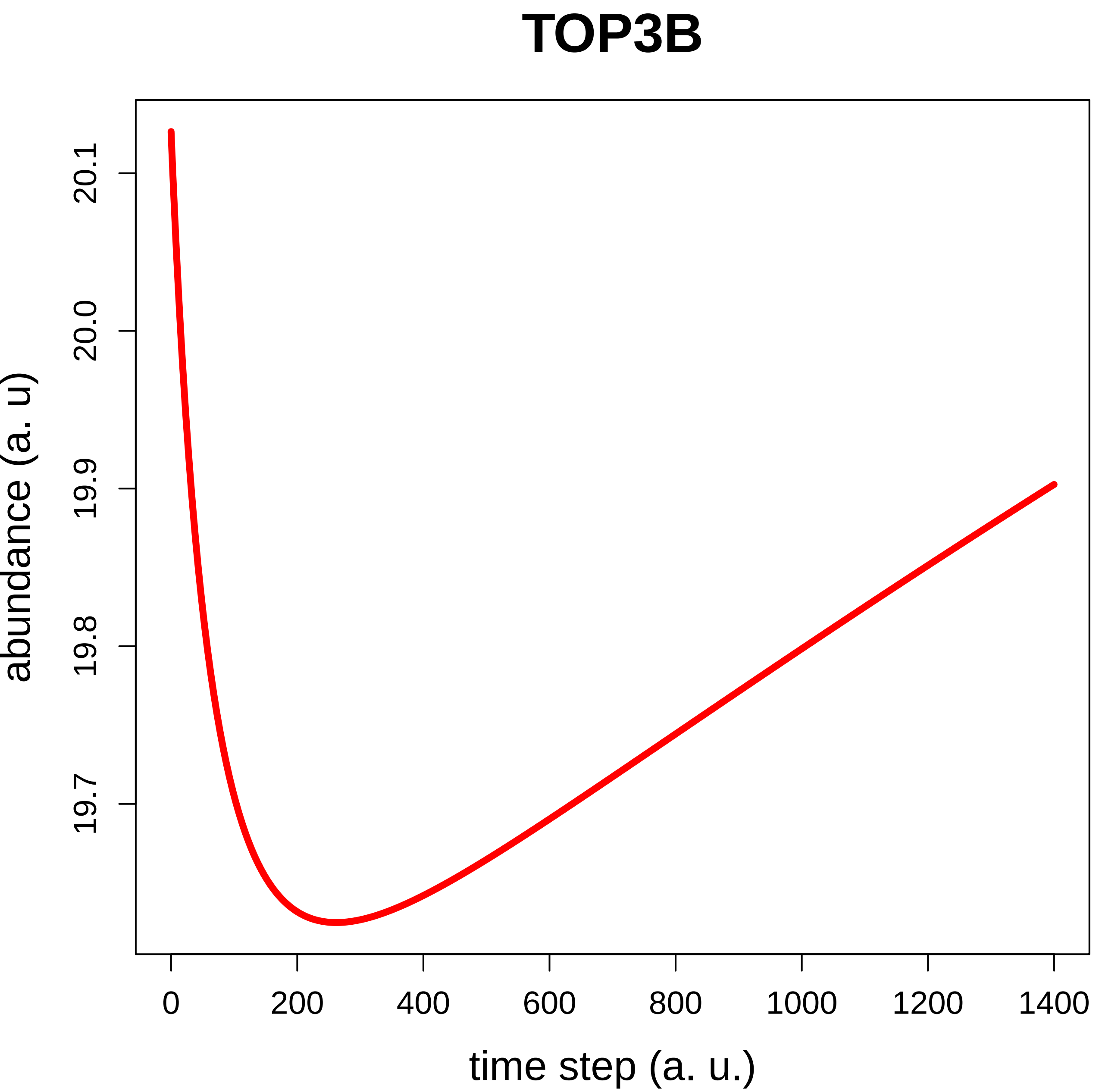
TOP3B
abundance (a. u)
20.1
20.0
19.9
19.8
19.7
0
200
400
600
800
1000
1200
1400
time step (a. u.)

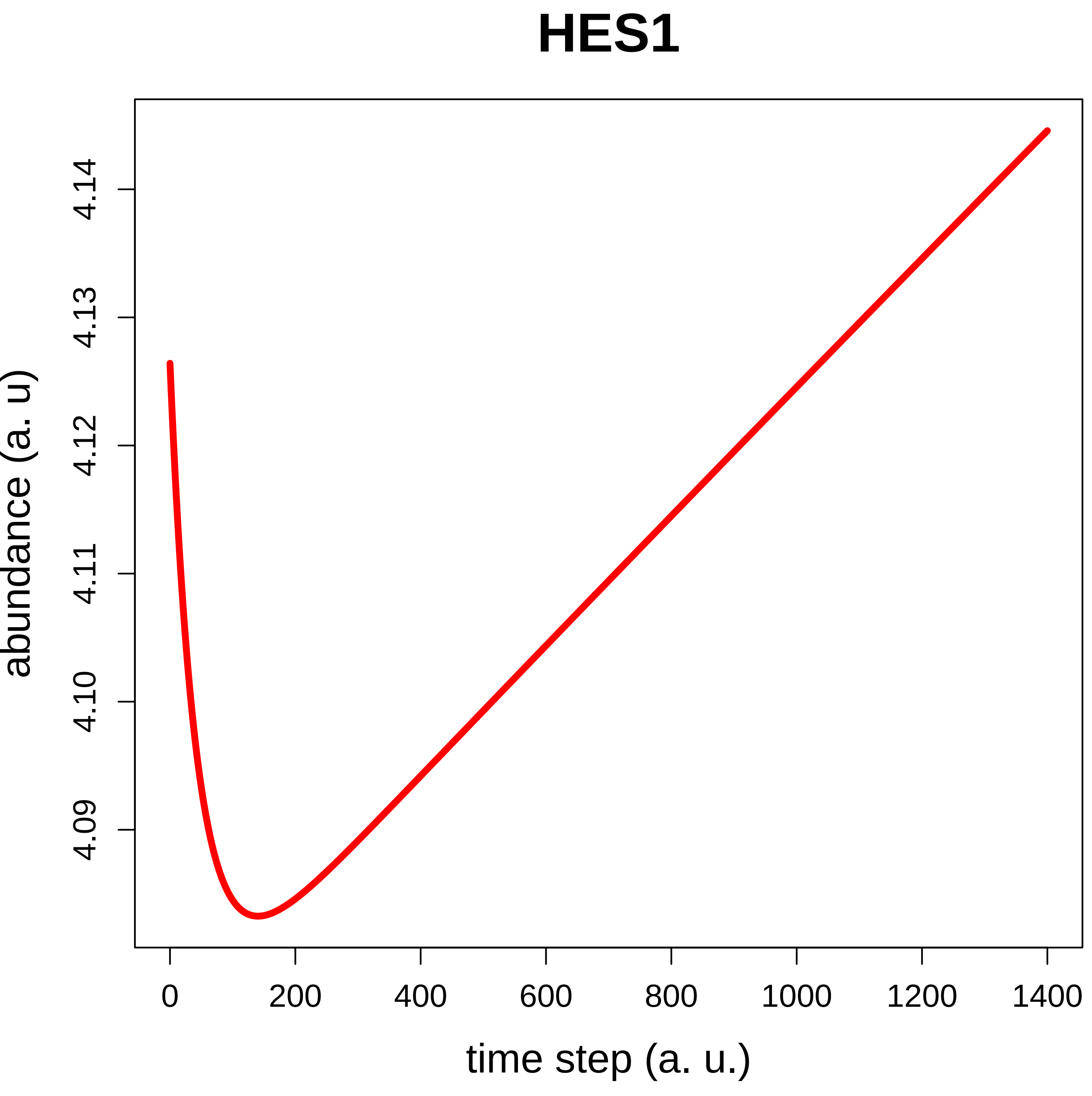

HES1
abundance (a. u)
4.09
4.10
4.11
4.12
4.13
4.14
0
200
400
600
800
1000
1200
1400
time step (a. u.)
