# Supplementary material for: Dynamic Modelling of DNA Repair Pathway at the Molecular Level: A New Perspective
Source: Front Mol Biosci. 2022 Sep 13;9:878148. doi: 10.3389/fmolb.2022.878148 (PMC9513183; doi:10.3389/fmolb.2022.878148)
Supplement: Supplementary file 1 [file Presentation1.zip › Submitted_Supplementary_Material/Simulations_of_Dynamics_HR_pathways.pdf]

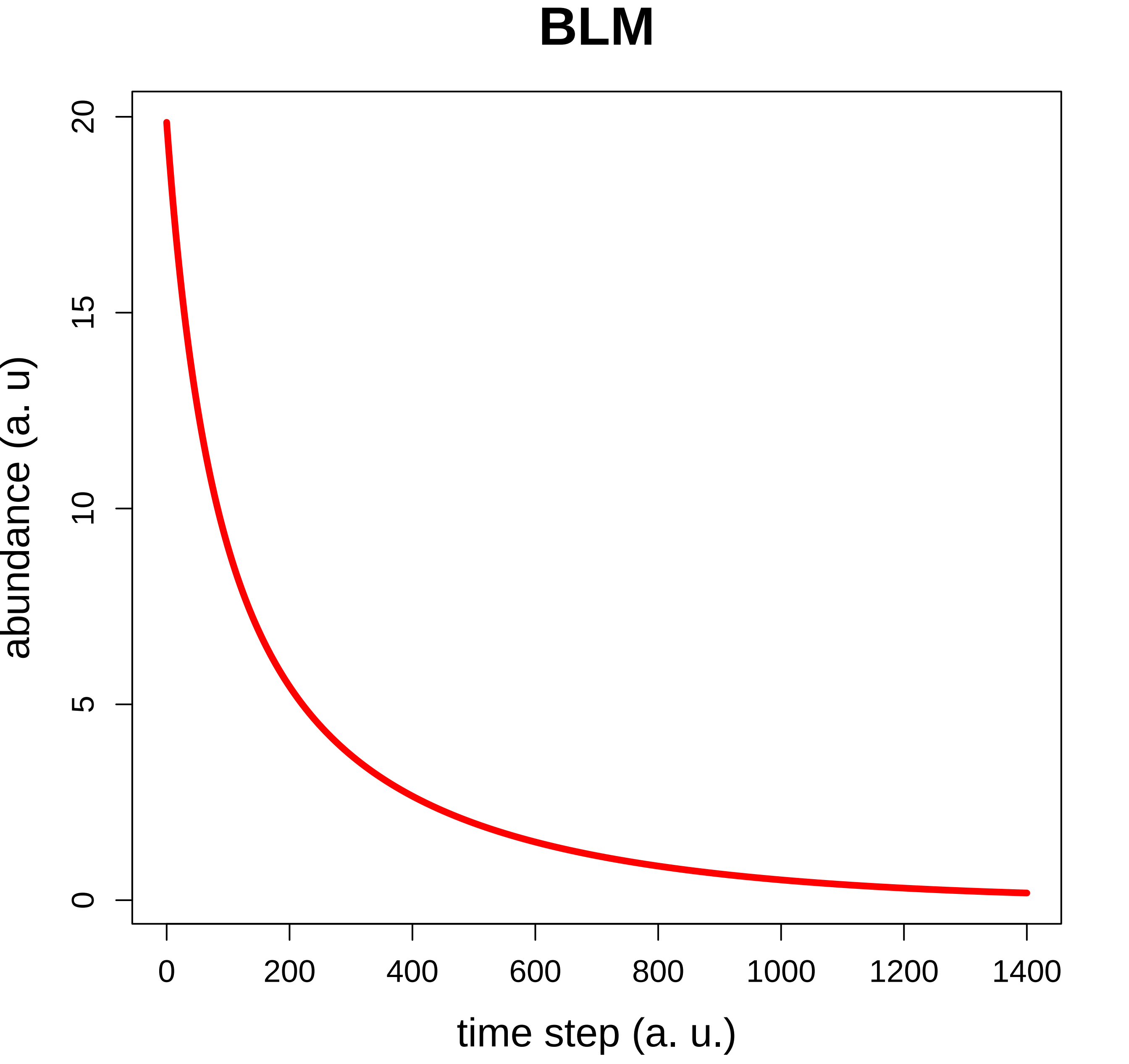

BLM
abundance (a. u)
time step (a. u.)
0
5
10
15
20
0
200
400
600
800
1000
1200
1400

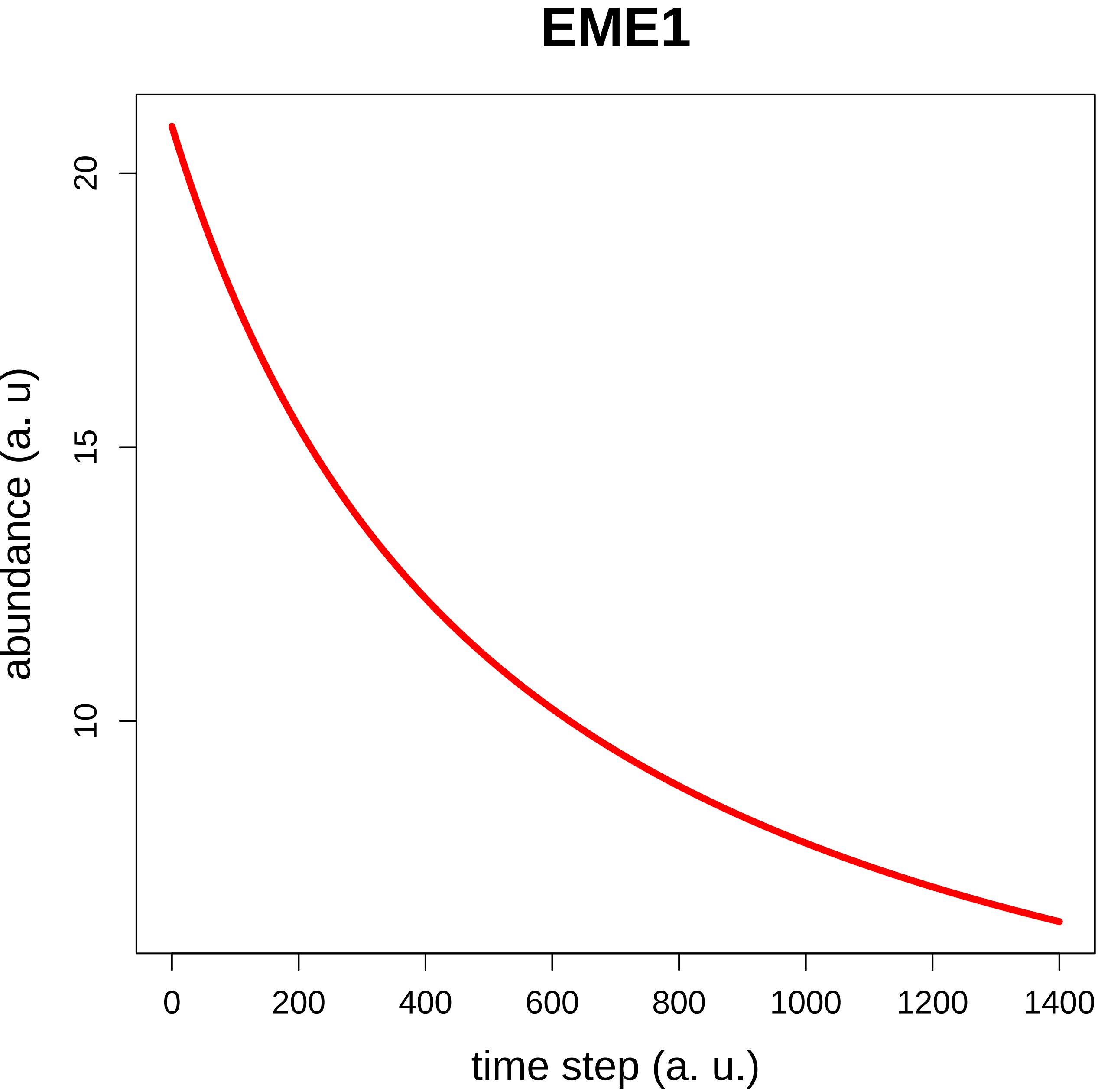

EME1
20
15
10
abundance (a. u)
0
200
400
600
800
1000
1200
1400
time step (a. u.)

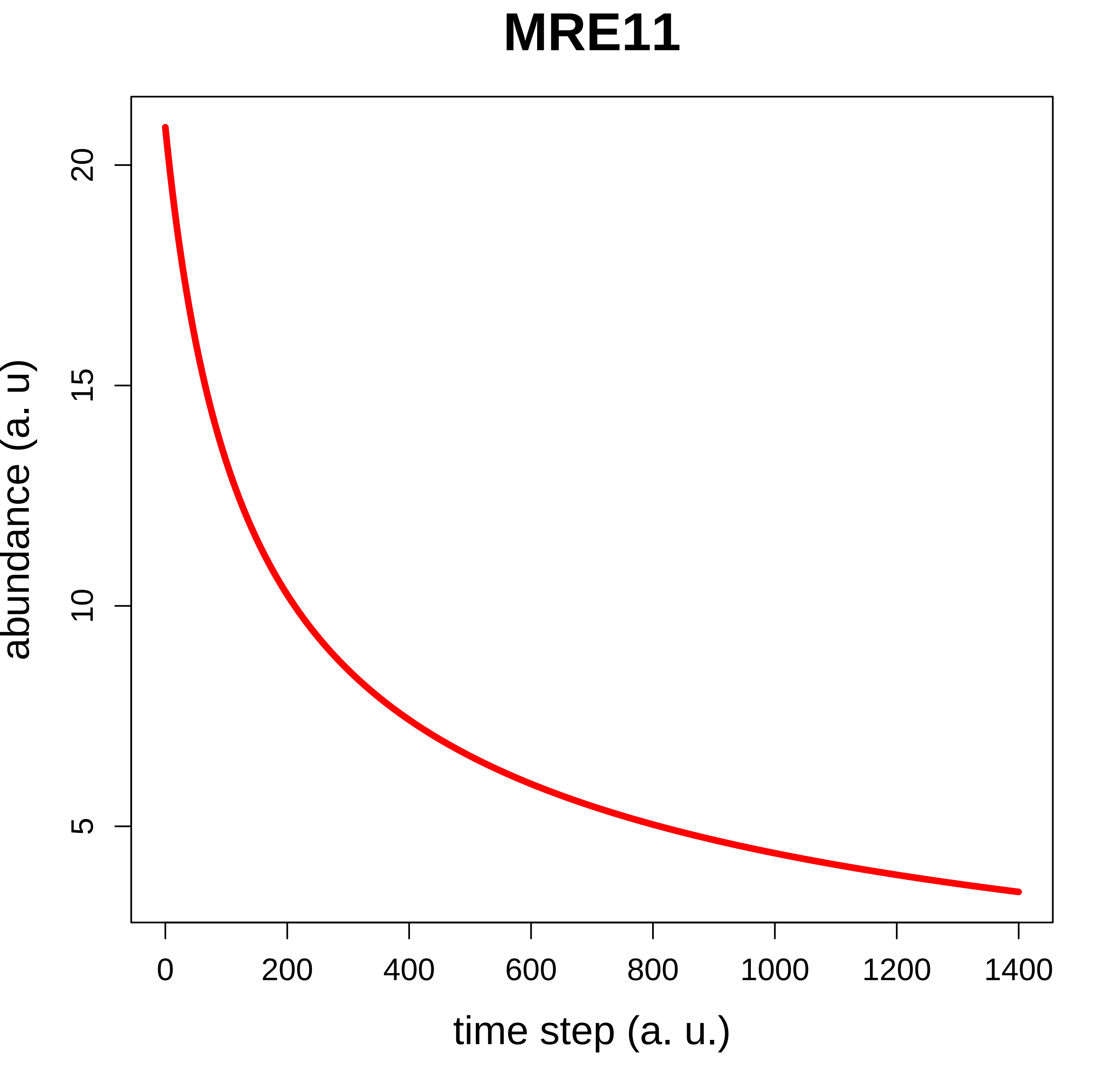

MRE11
abundance (a. u)
20
15
10
5
0
200
400
600
800
1000
1200
1400
time step (a. u.)

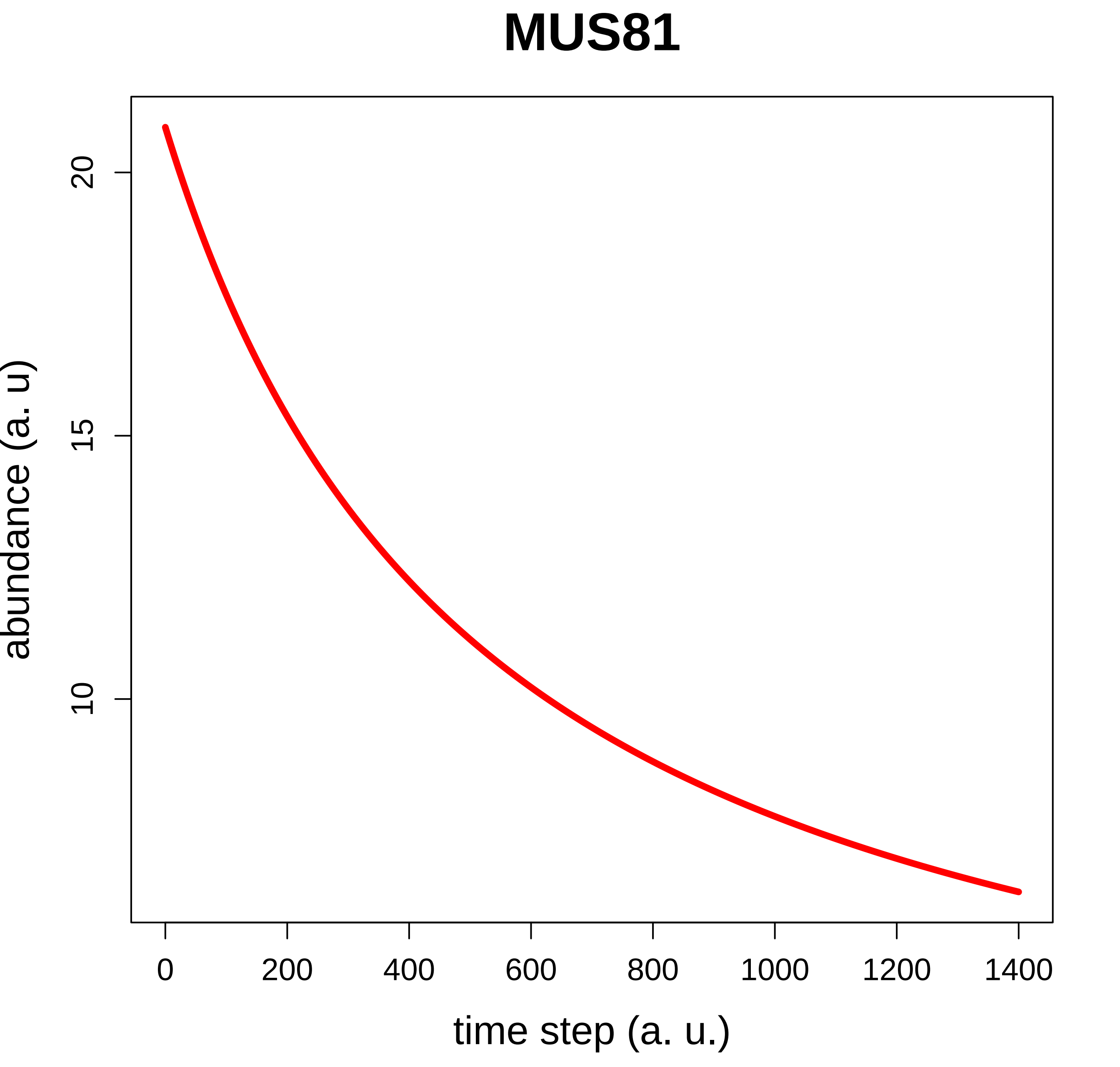

MUS81
abundance (a. u)
20
15
10
0
200
400
600
800
1000
1200
1400
time step (a. u.)

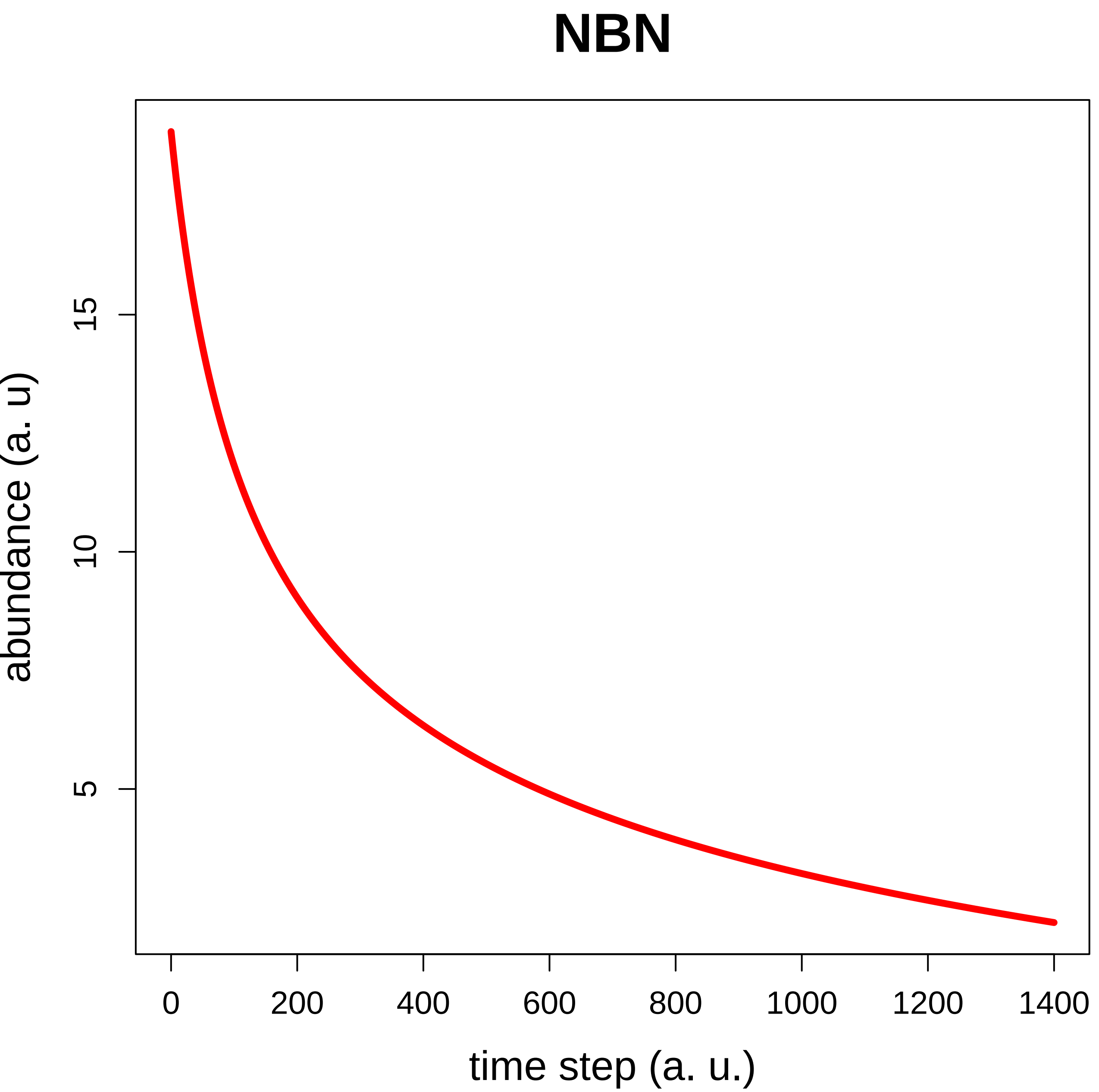

NBN
15
10
5
abundance (a. u)
0
200
400
600
800
1000
1200
1400
time step (a. u.)

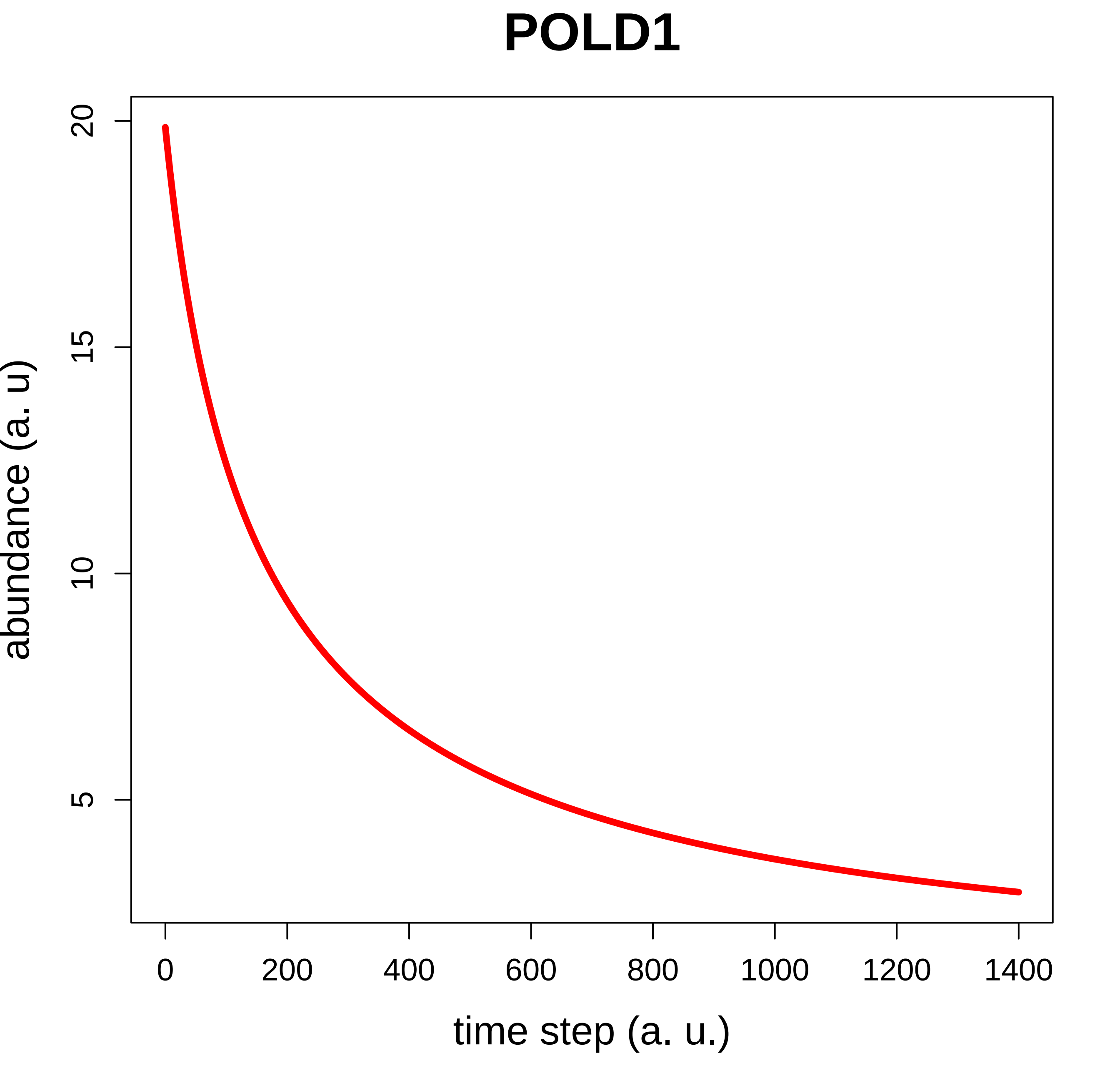
POLD1
abundance (a. u)
20
15
10
5
0
200
400
600
800
1000
1200
1400
time step (a. u.)

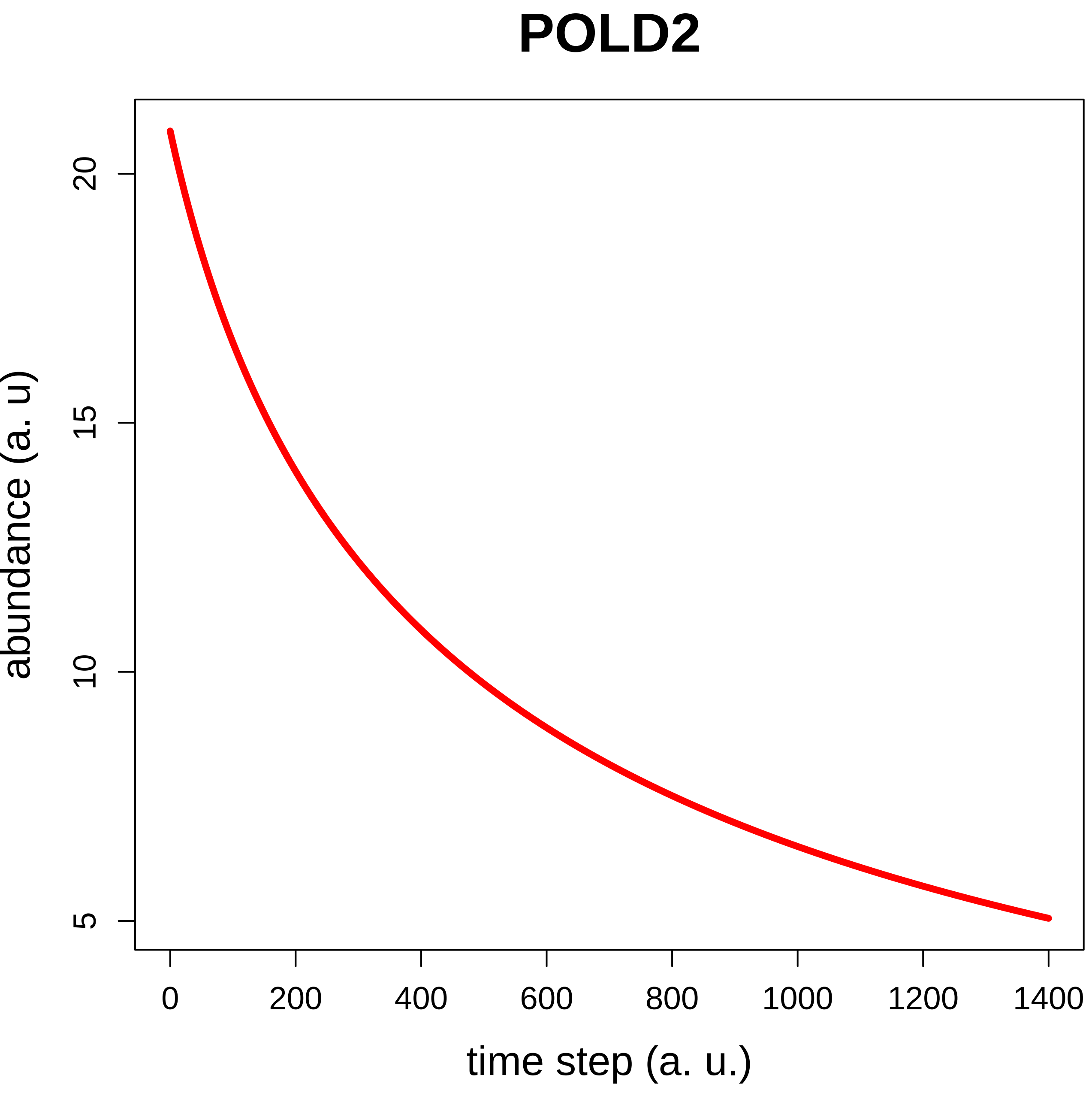
POLD2
abundance (a. u)
20
15
10
5
0
200
400
600
800
1000
1200
1400
time step (a. u.)

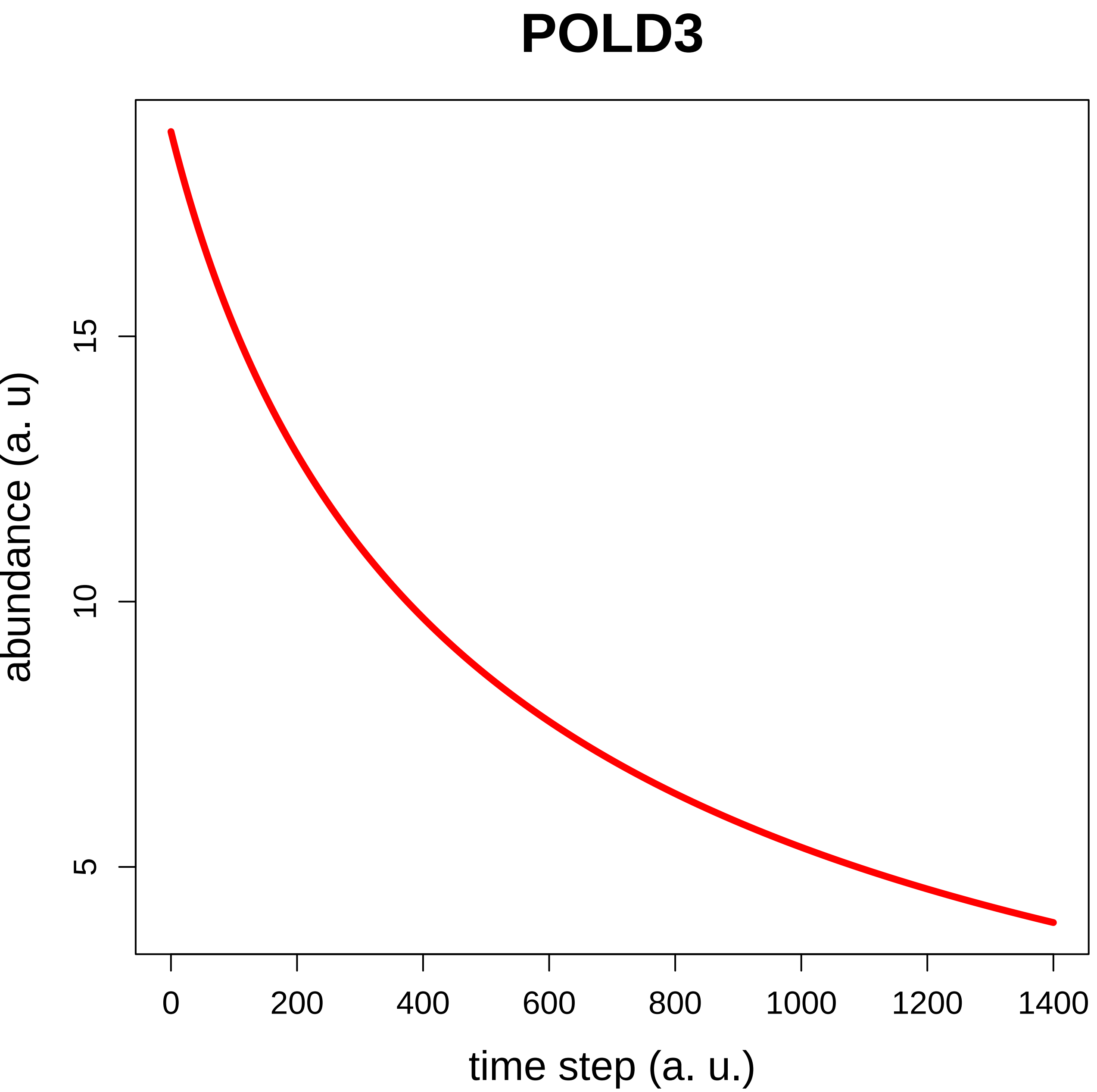

POLD3
abundance (a. u)
time step (a. u.)
5
10
15
0
200
400
600
800
1000
1200
1400

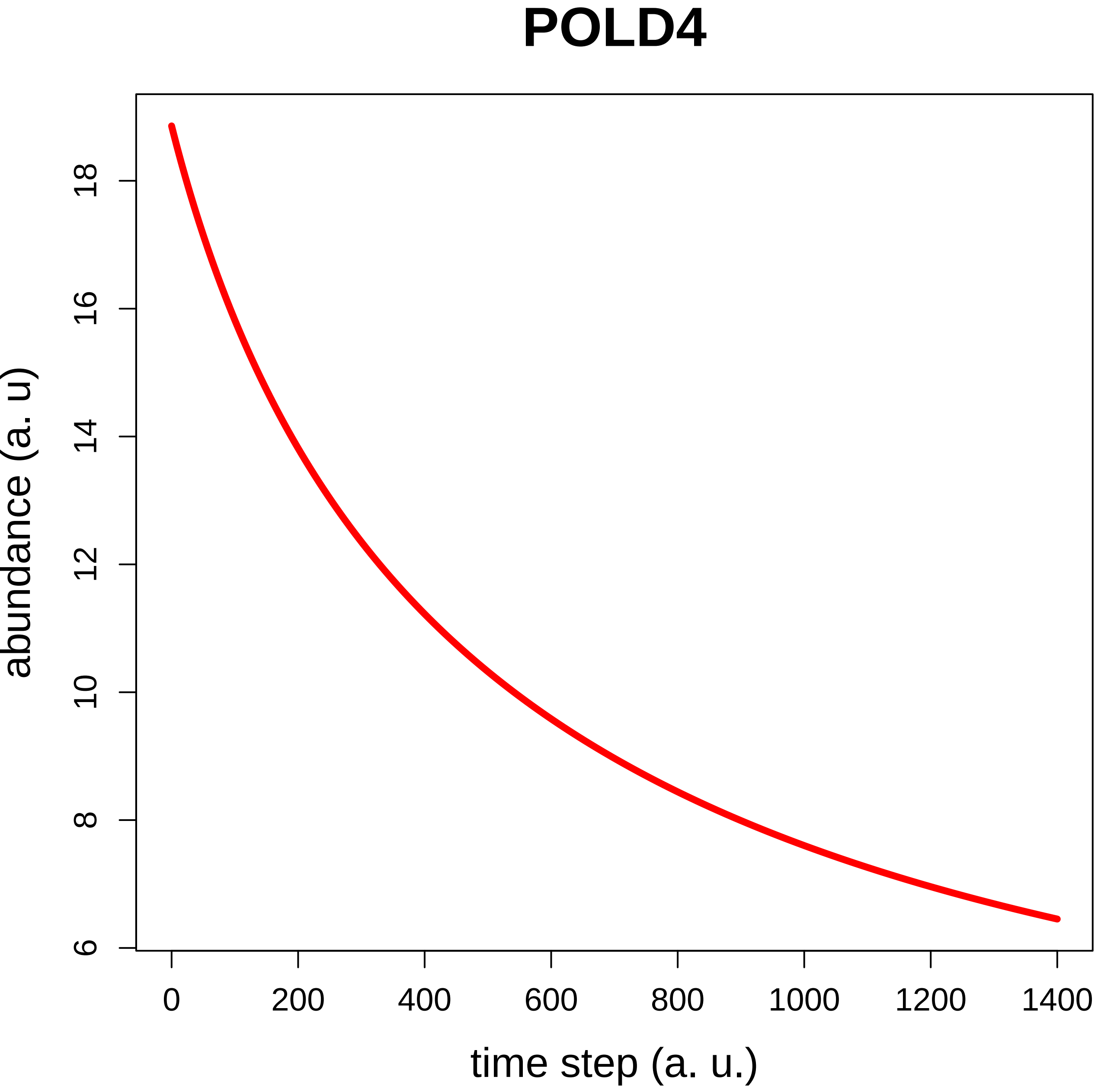

POLD4
abundance (a. u)
time step (a. u.)
6
8
10
12
14
16
18
0
200
400
600
800
1000
1200
1400

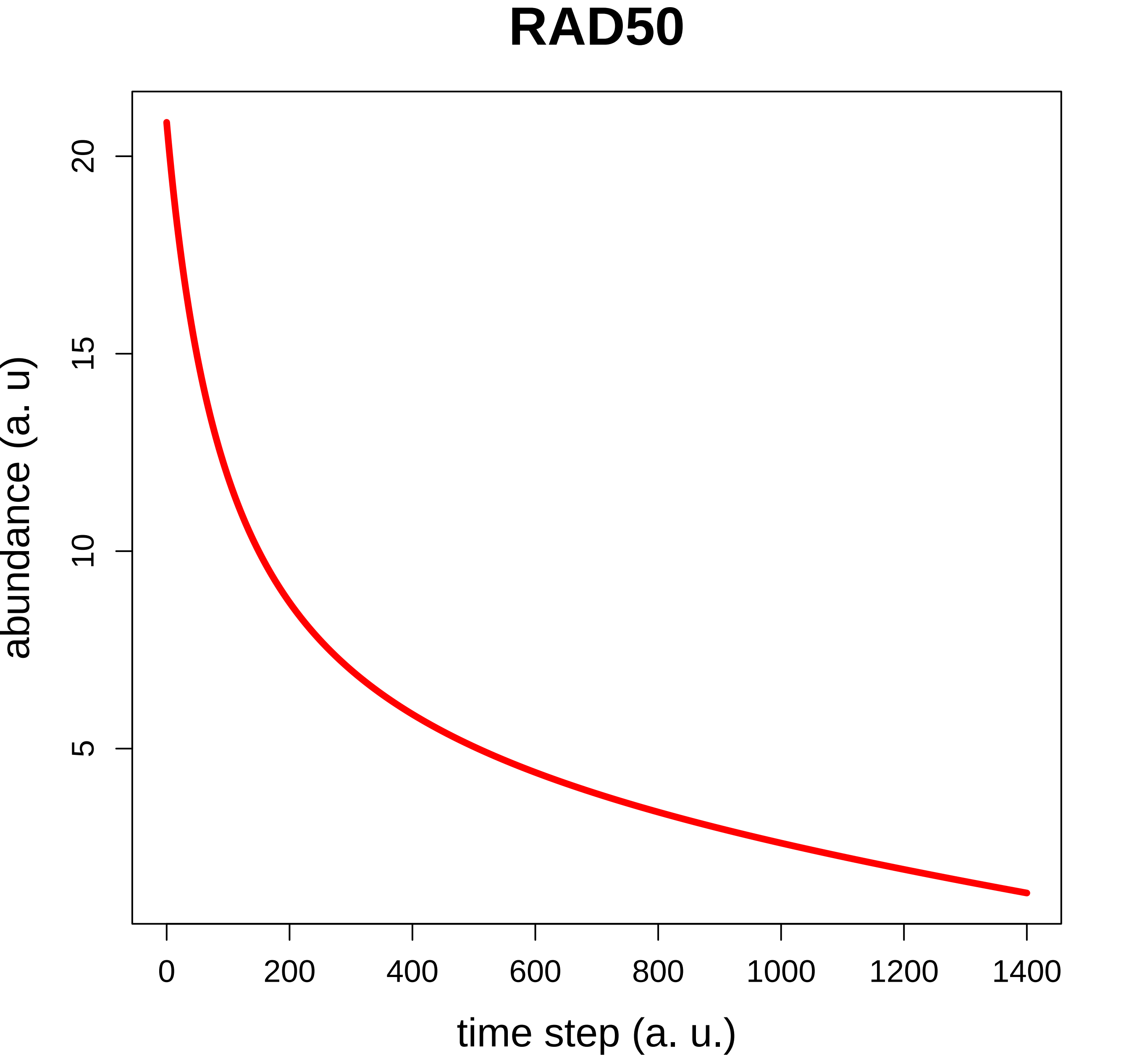
RAD50
abundance (a. u)
5
10
15
20
0
200
400
600
800
1000
1200
1400
time step (a. u.)

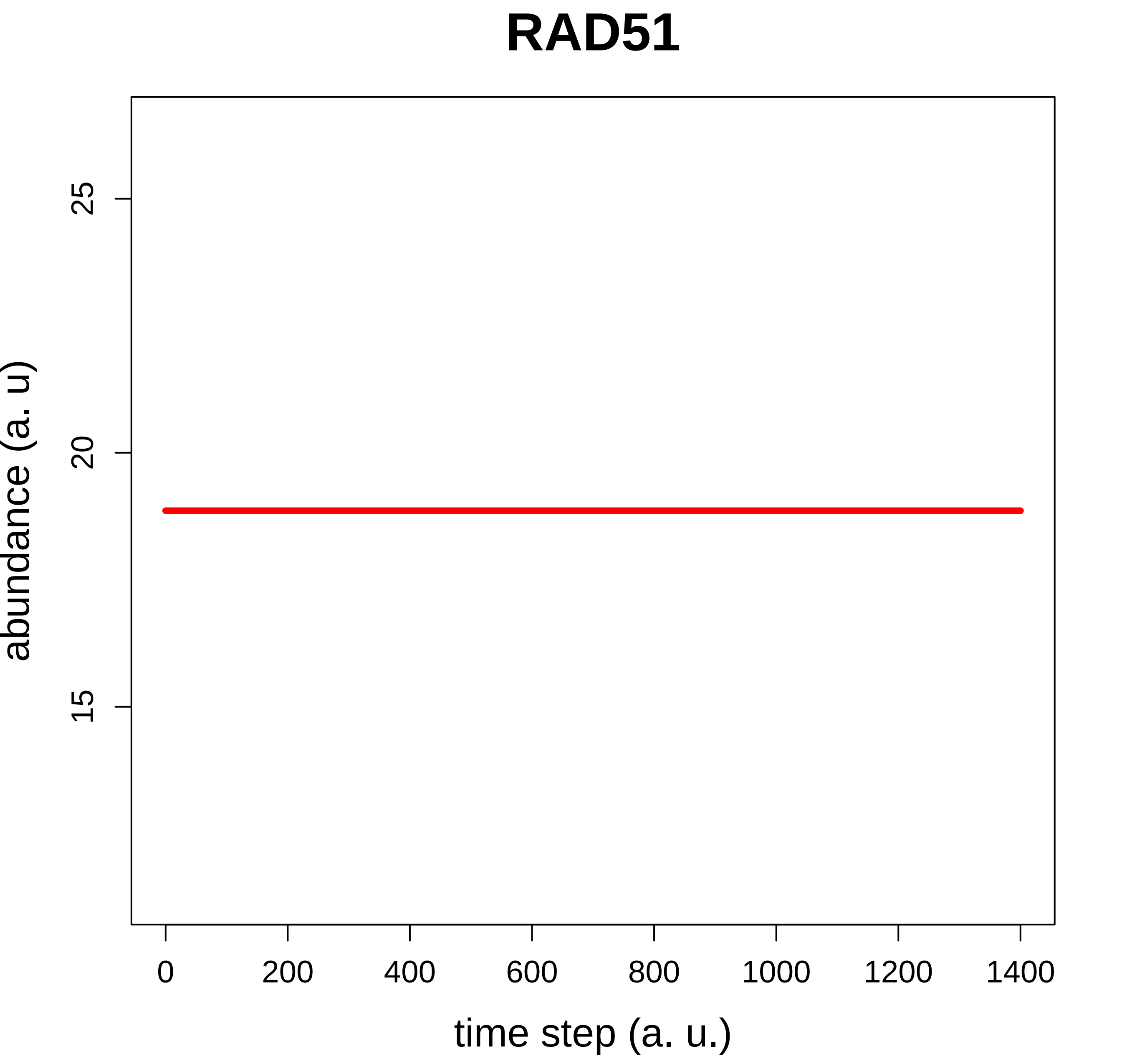

RAD51
abundance (a. u)
25
20
15
0
200
400
600
800
1000
1200
1400
time step (a. u.)

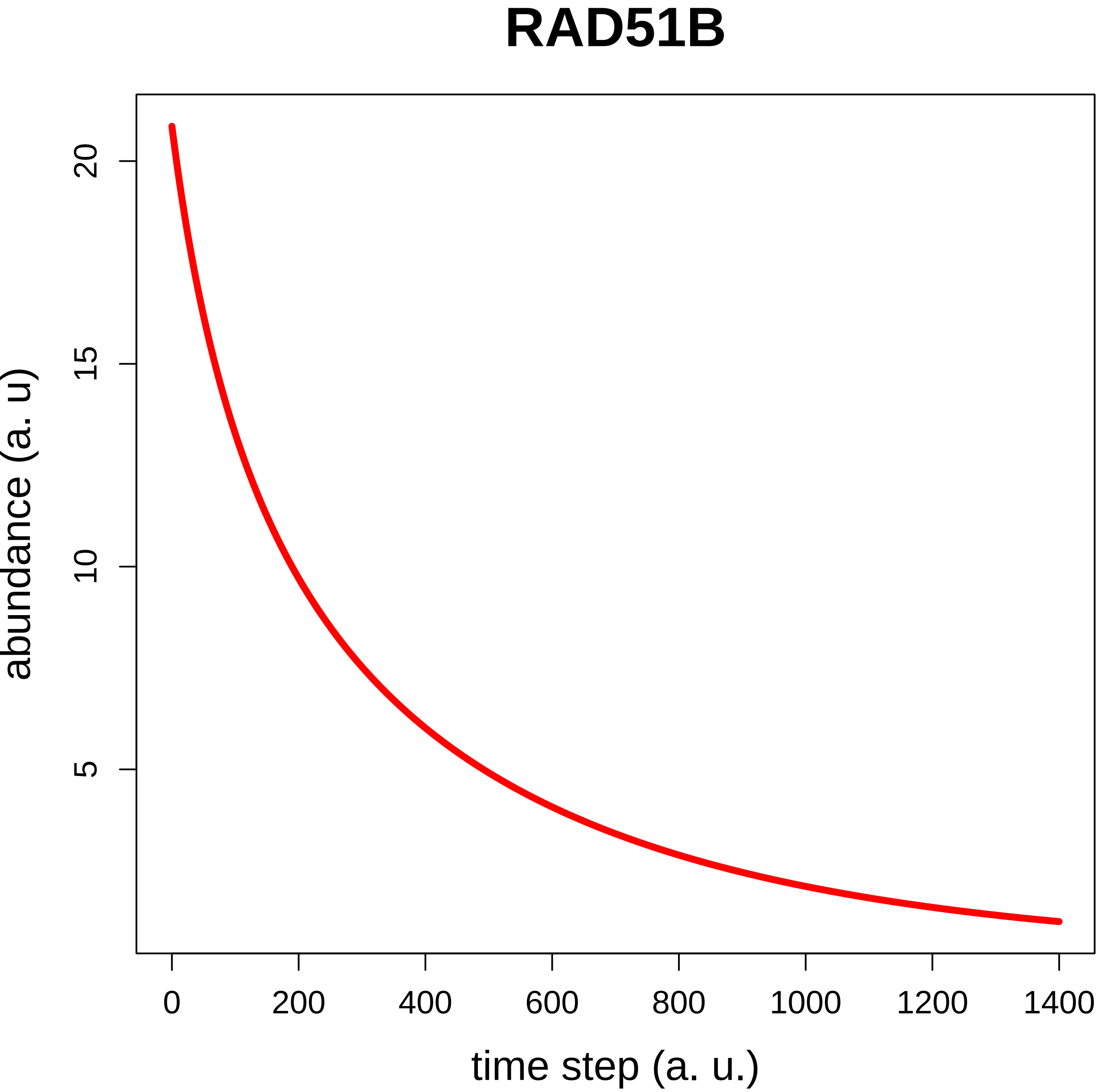

RAD51B
abundance (a. u)
20
15
10
5
0
200
400
600
800
1000
1200
1400
time step (a. u.)

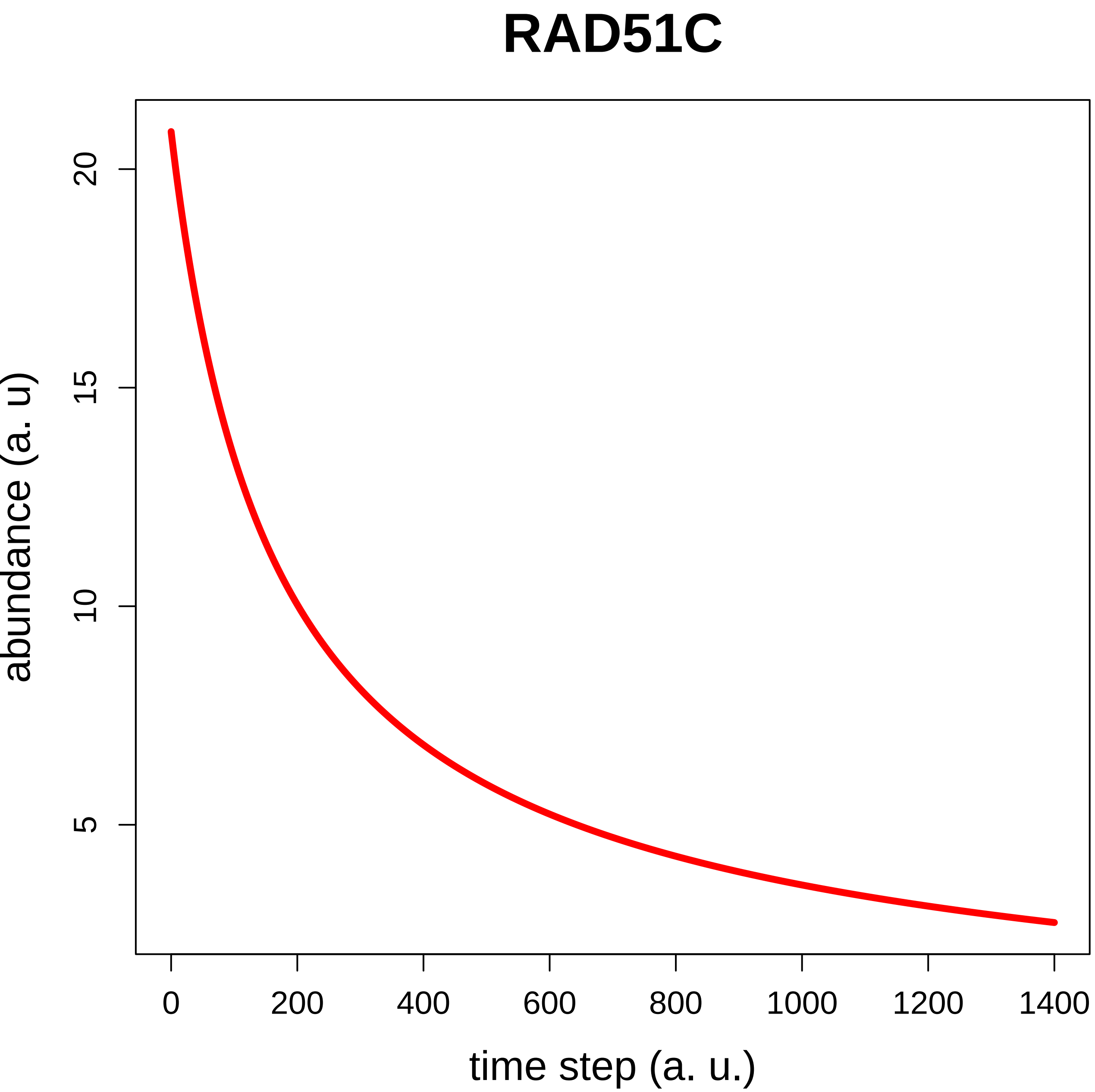
RAD51C
abundance (a. u)
time step (a. u.)
0
200
400
600
800
1000
1200
1400
5
10
15
20

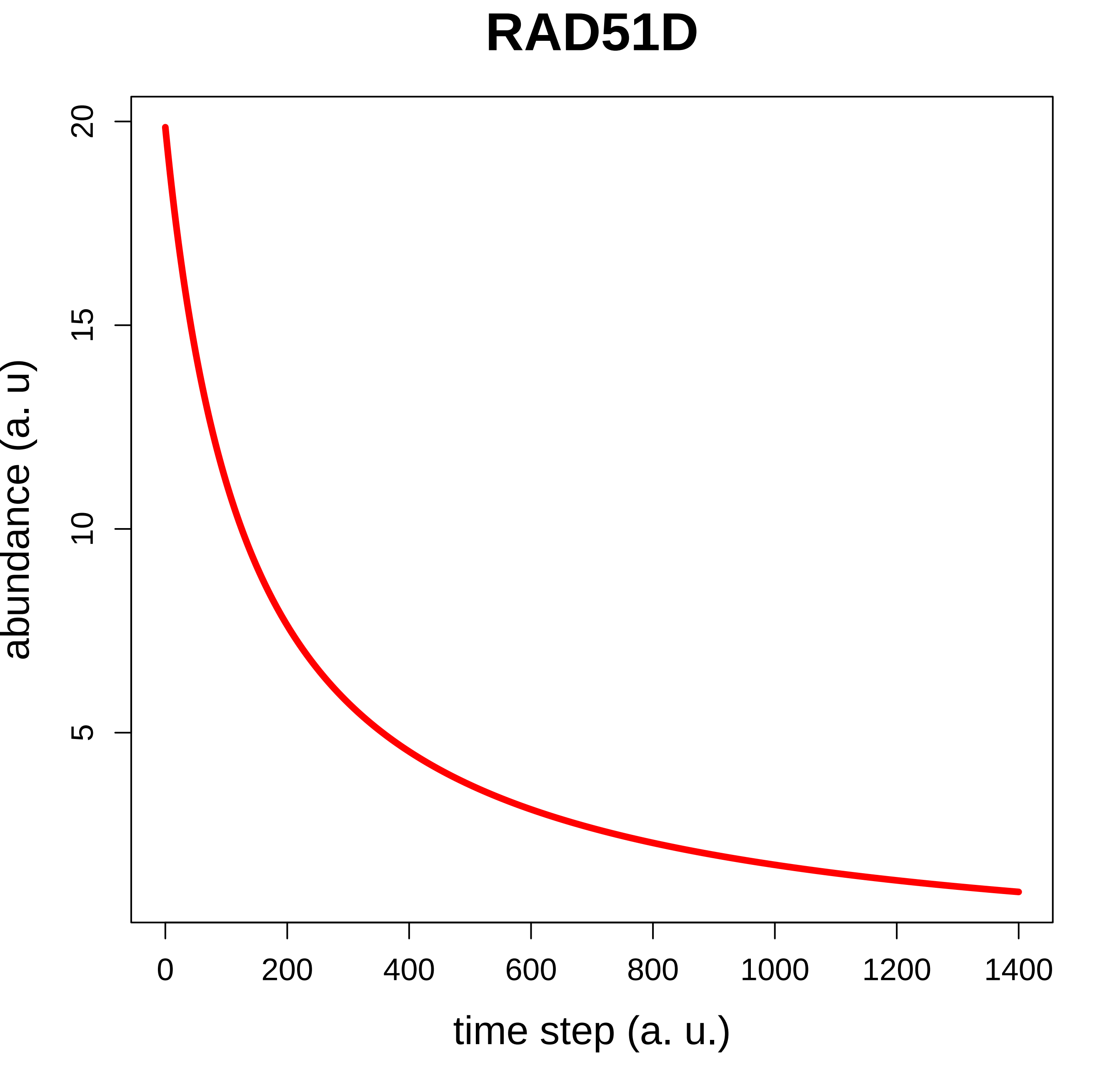

RAD51D
abundance (a. u)
20
15
10
5
0
200
400
600
800
1000
1200
1400
time step (a. u.)

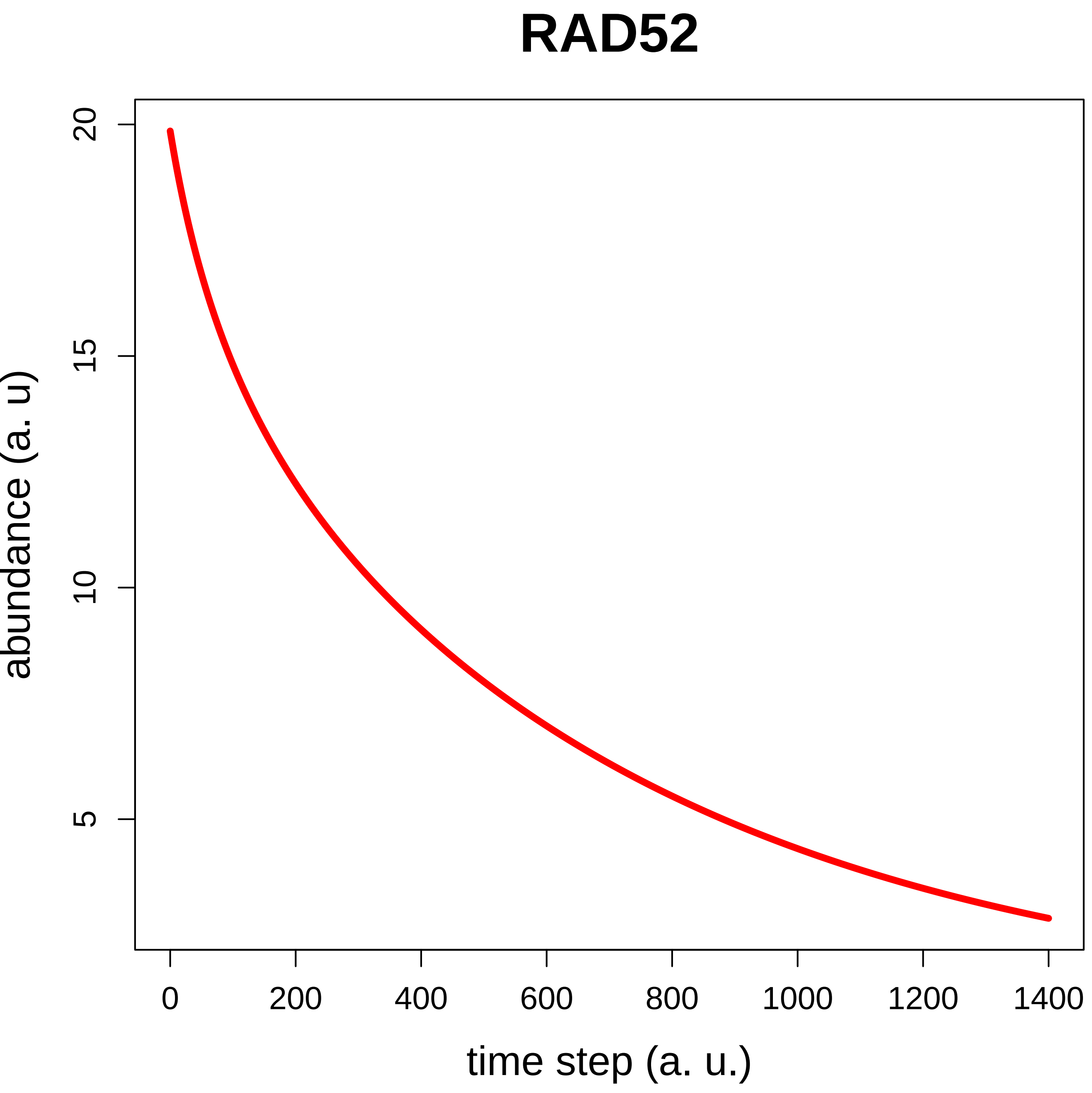
RAD52
abundance (a. u)
20
15
10
5
0
200
400
600
800
1000
1200
1400
time step (a. u.)

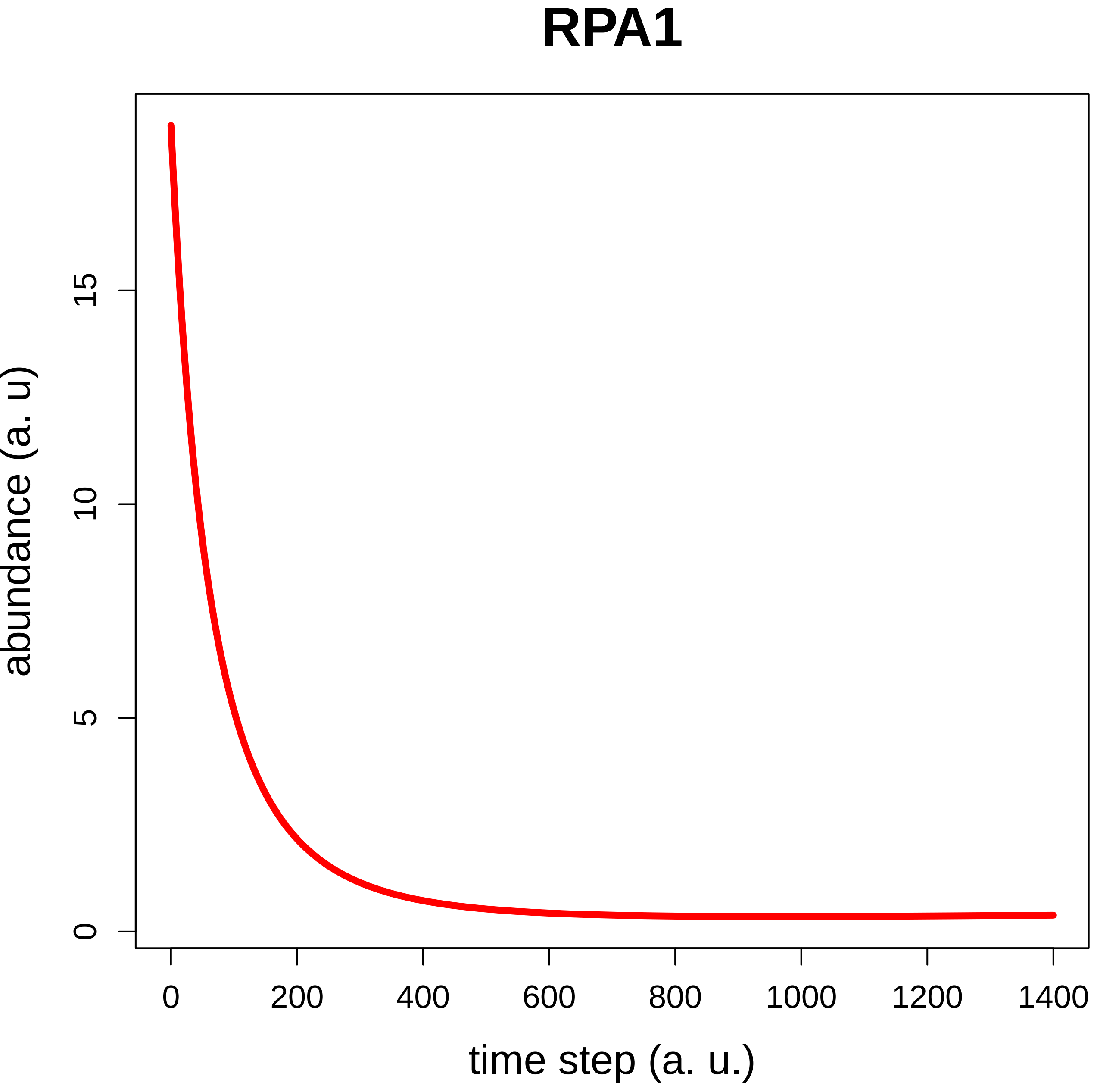

RPA1
abundance (a. u)
time step (a. u.)
0
5
10
15
0
200
400
600
800
1000
1200
1400

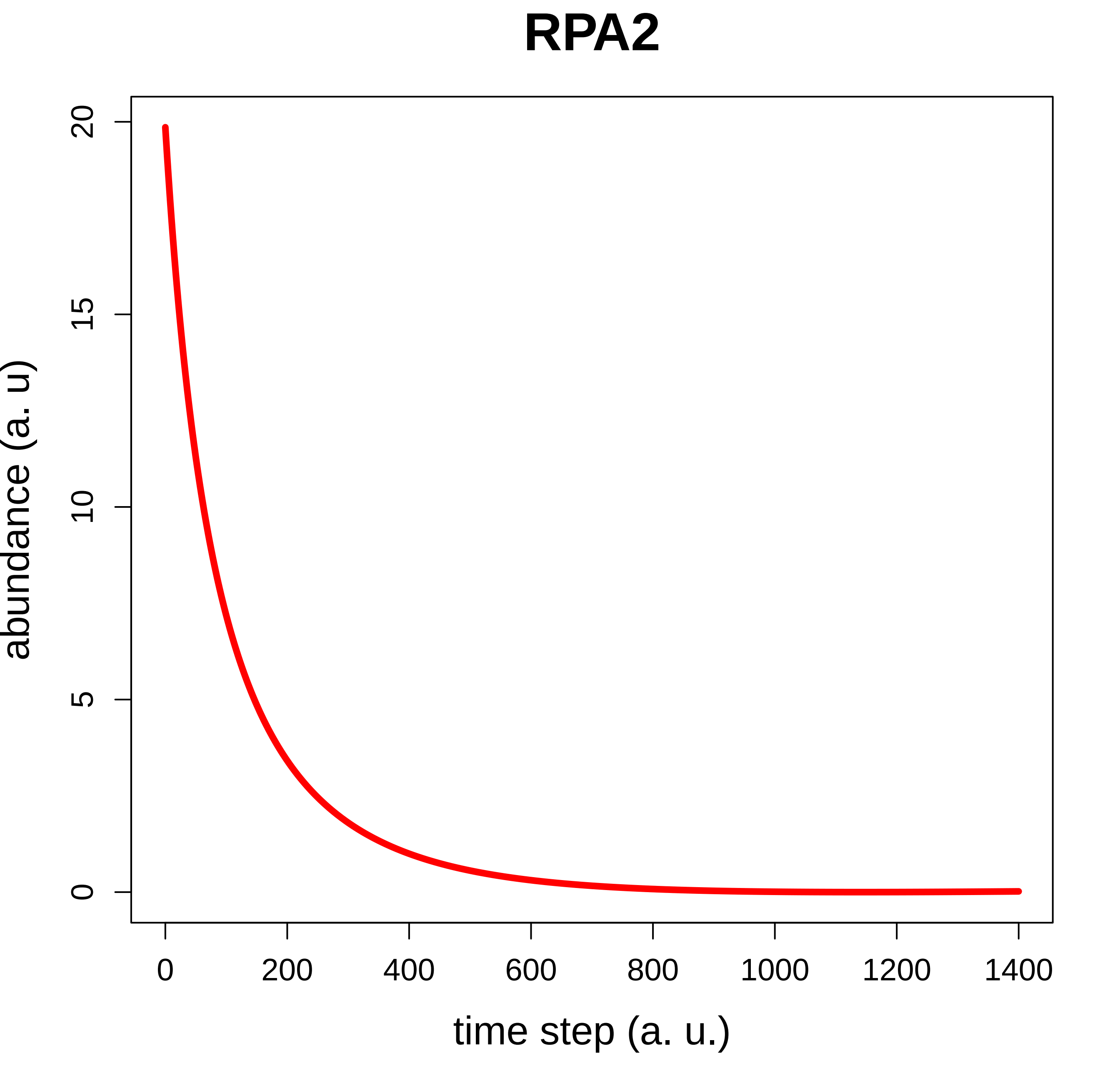
RPA2
abundance (a. u)
0
5
10
15
20
0
200
400
600
800
1000
1200
1400
time step (a. u.)

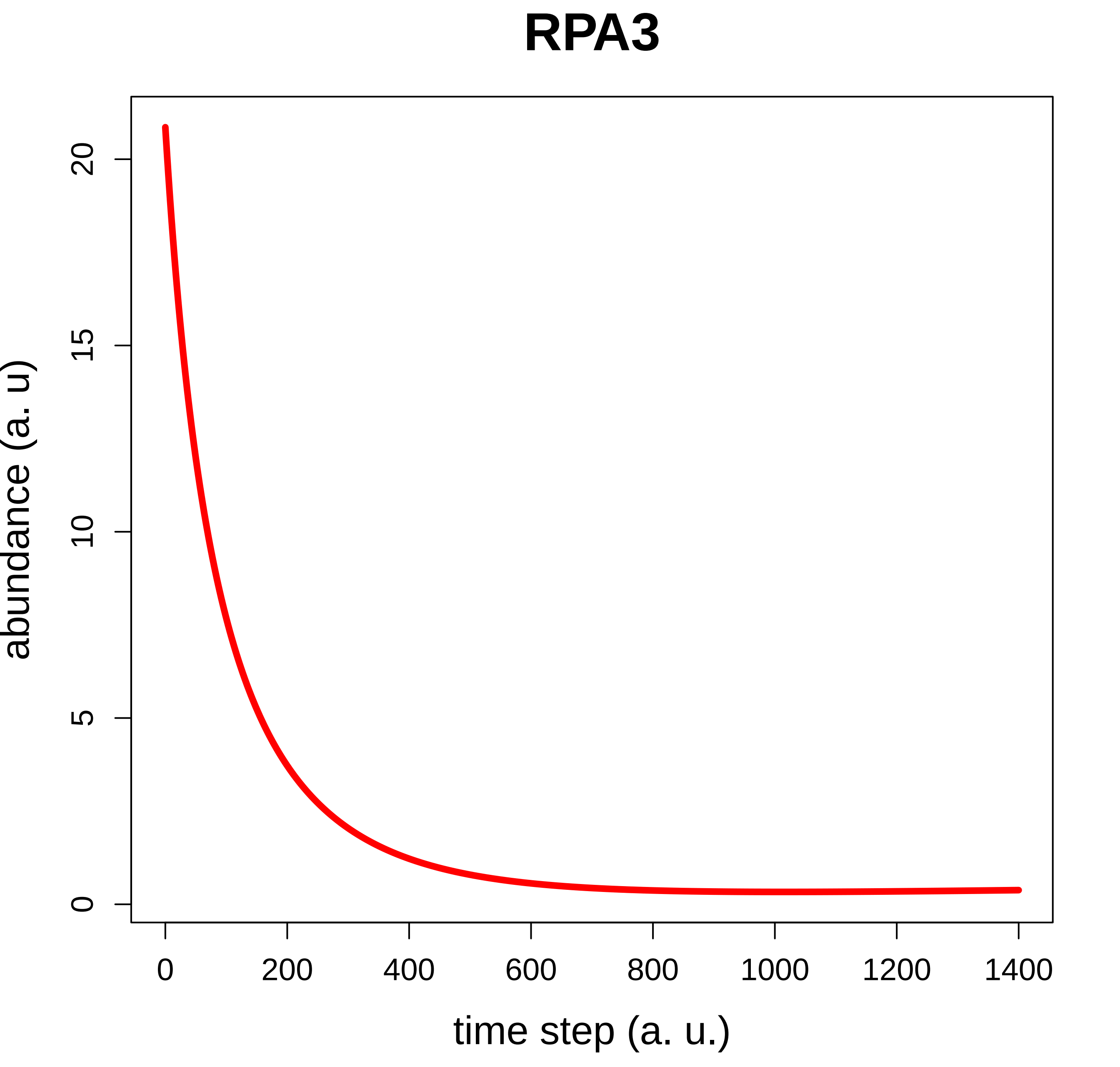
RPA3
abundance (a. u)
time step (a. u.)
0
5
10
15
20
0
200
400
600
800
1000
1200
1400

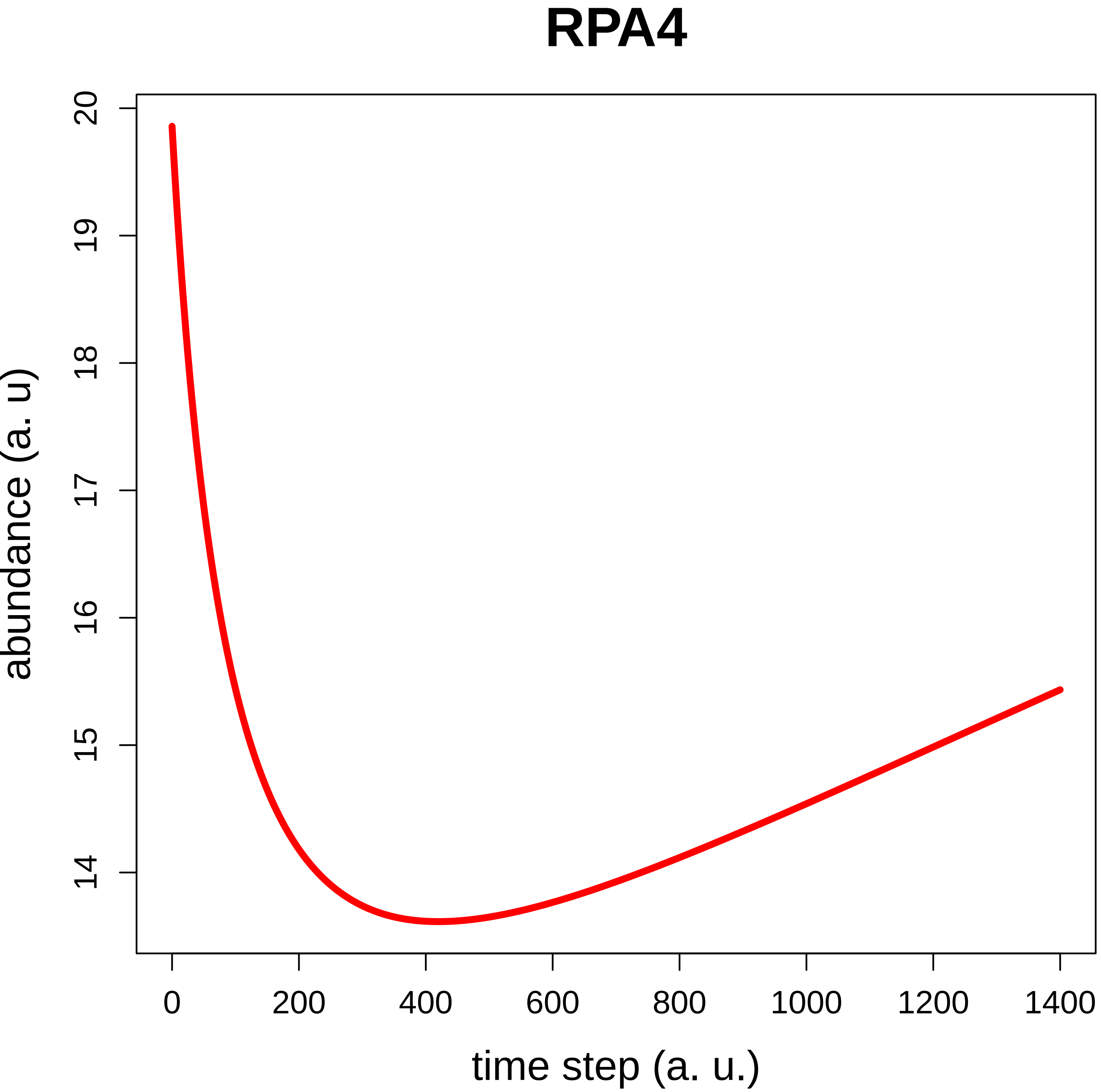

RPA4
abundance (a. u)
20
19
18
17
16
15
14
0
200
400
600
800
1000
1200
1400
time step (a. u.)

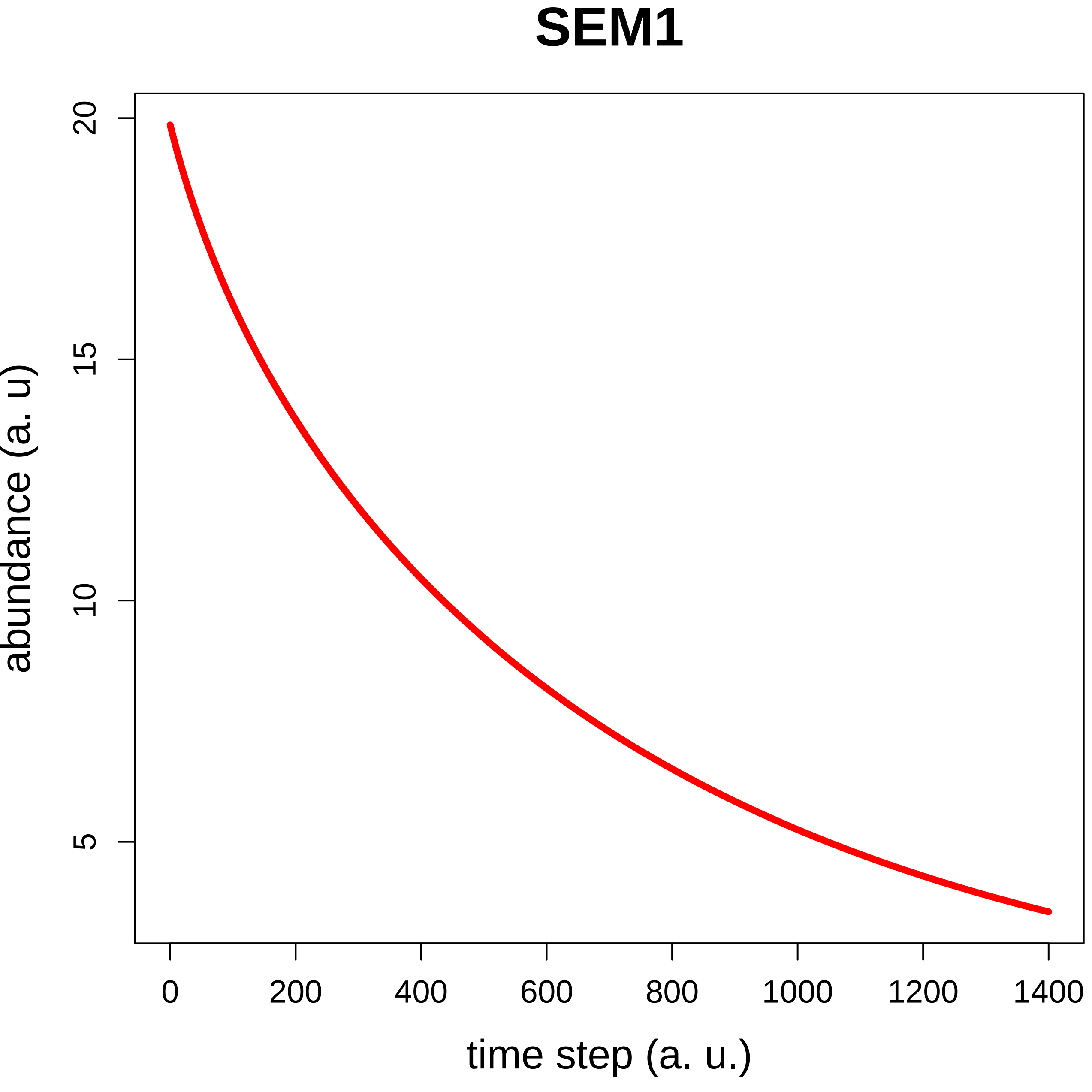

SEM1
abundance (a. u)
time step (a. u.)
5
10
15
20
0
200
400
600
800
1000
1200
1400

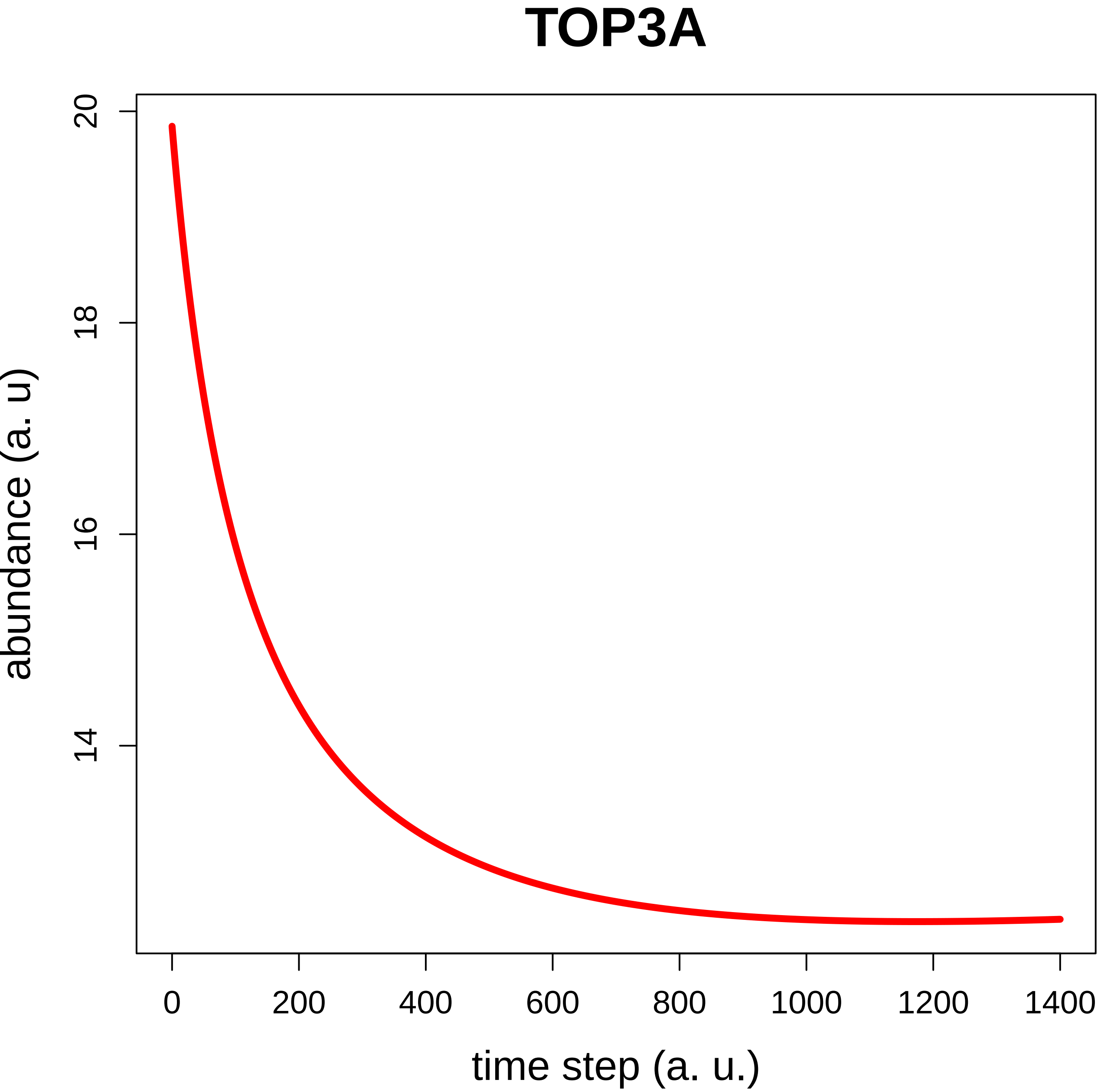

TOP3A
20
18
16
14
abundance (a. u)
0
200
400
600
800
1000
1200
1400
time step (a. u.)

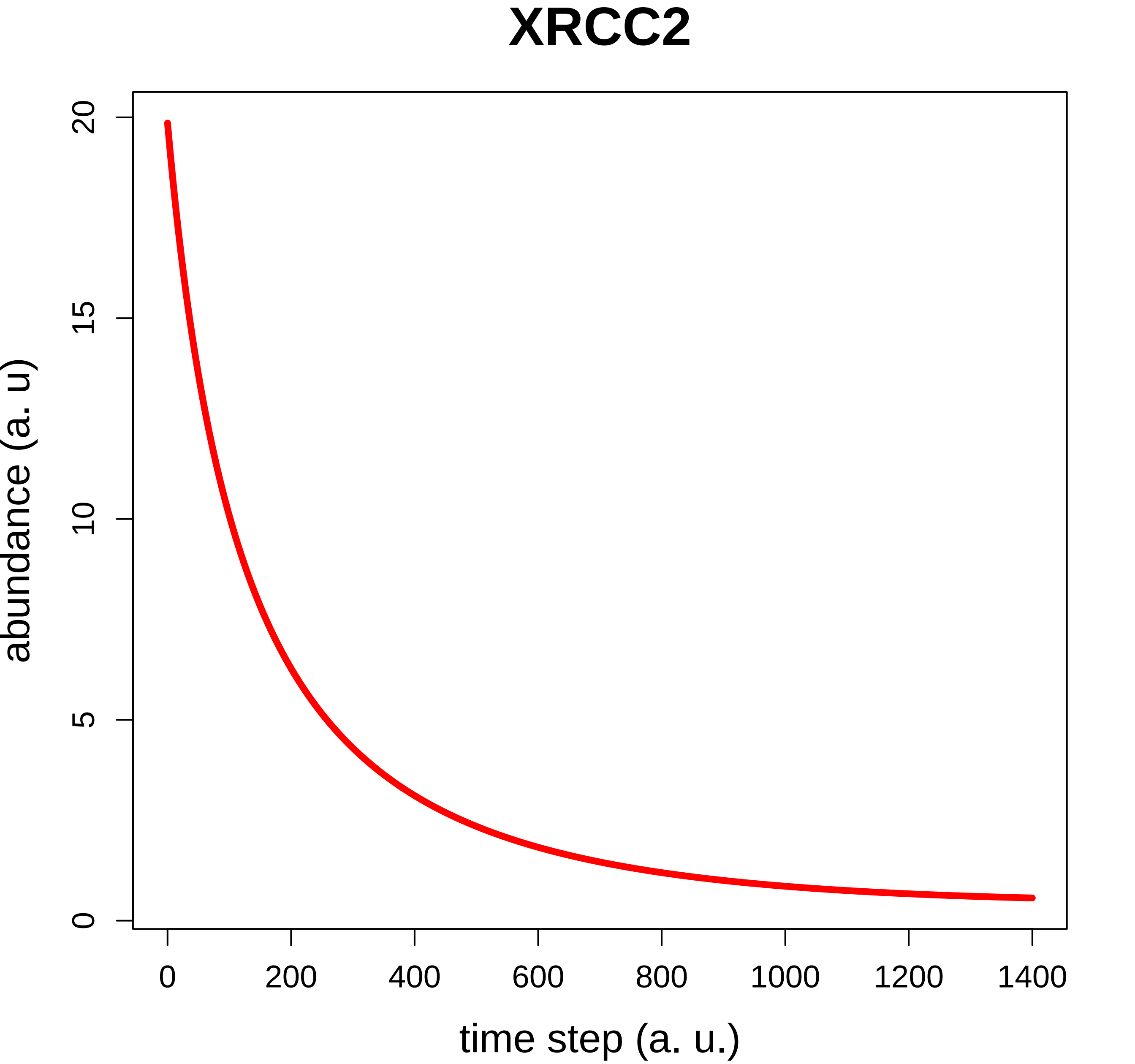
XRCC2
abundance (a. u)
0
5
10
15
20
0
200
400
600
800
1000
1200
1400
time step (a. u.)

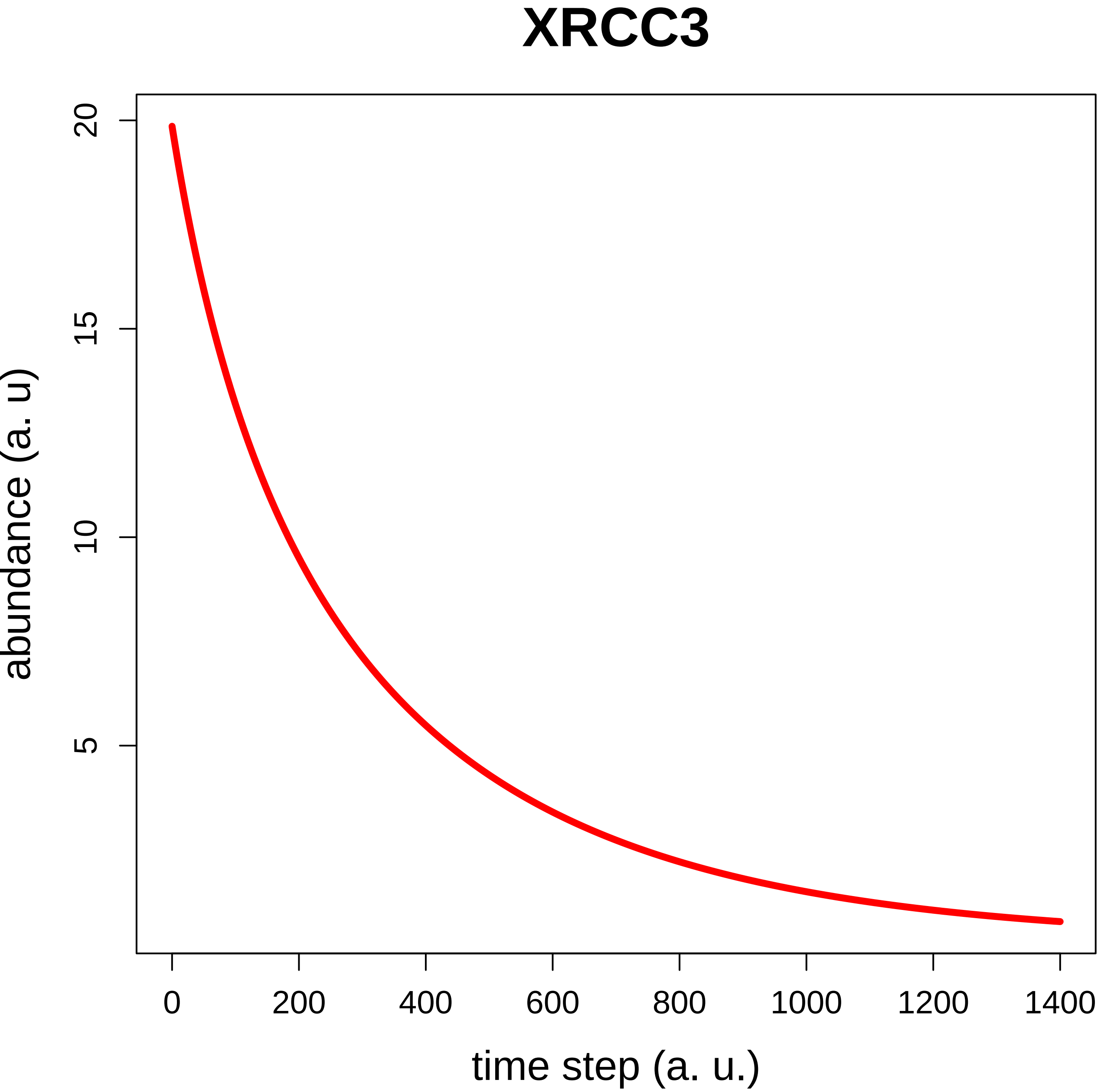
XRCC3
abundance (a. u)
20
15
10
5
0
200
400
600
800
1000
1200
1400
time step (a. u.)

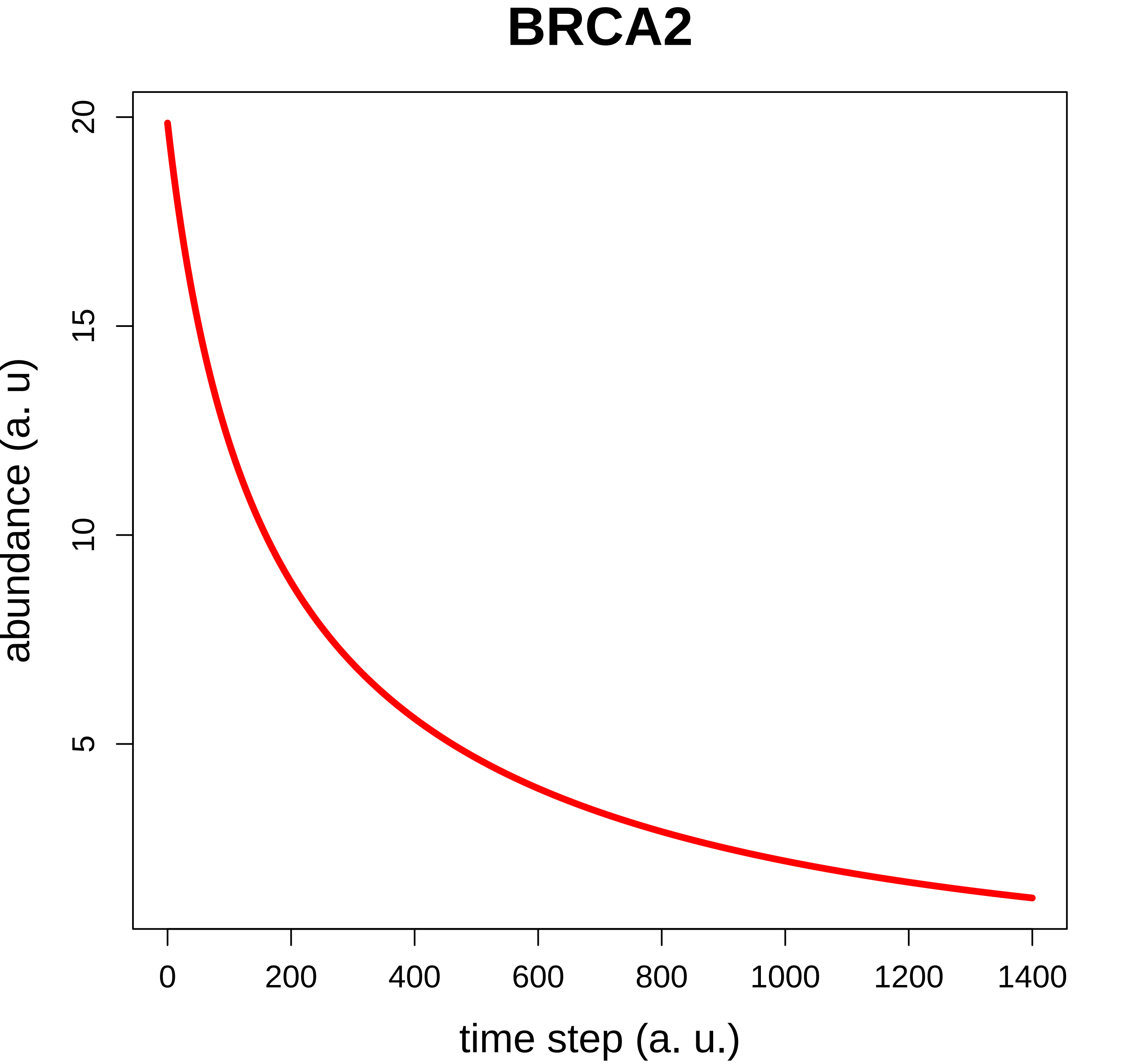
BRCA2
abundance (a. u)
time step (a. u.)
20
15
10
5
0
200
400
600
800
1000
1200
1400

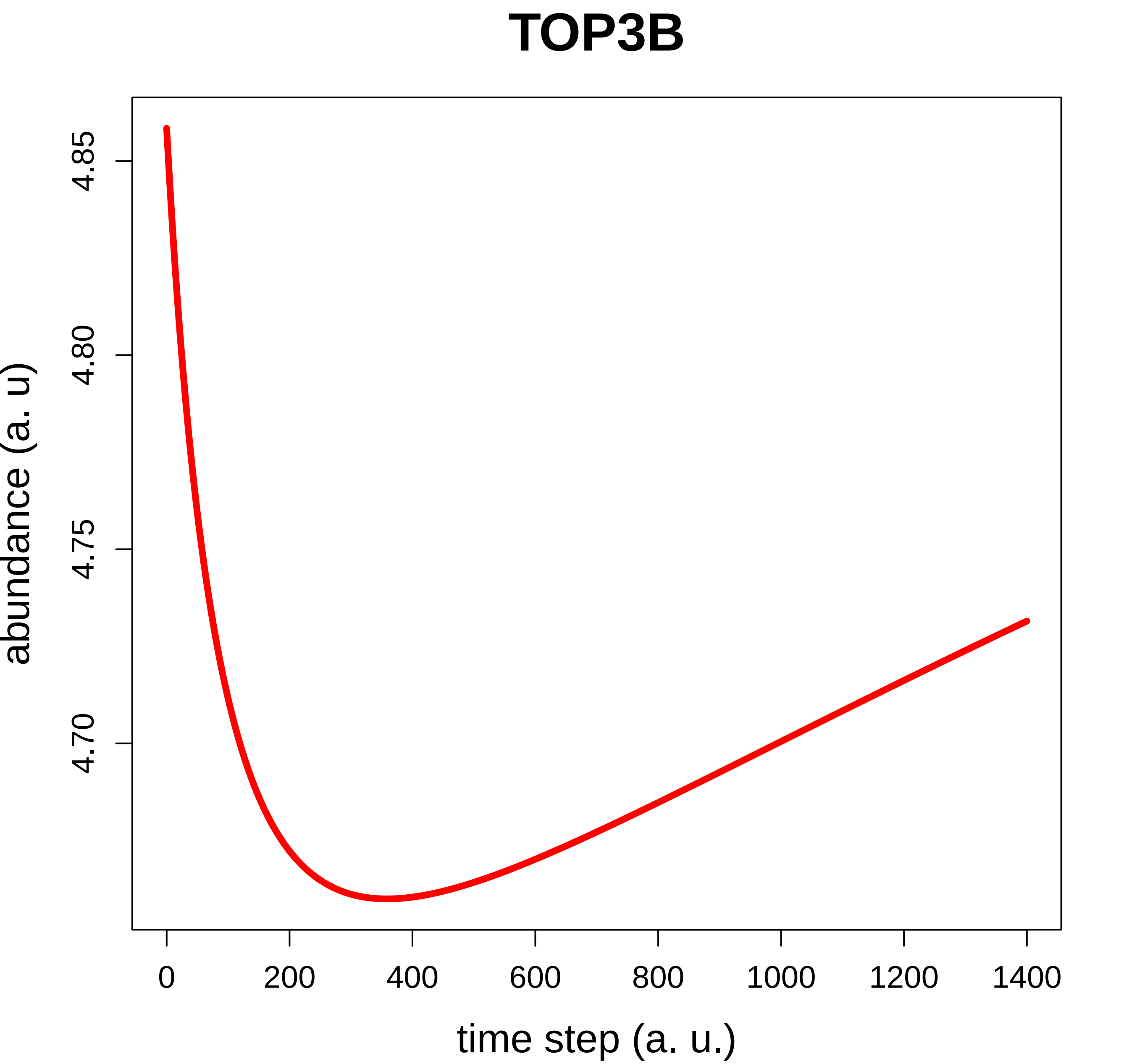
TOP3B
abundance (a. u)
time step (a. u.)
4.70
4.75
4.80
4.85
0
200
400
600
800
1000
1200
1400
